# Targeting gut microbiota–derived kynurenine to predict and protect the remodeling of the pressure-overloaded young heart

**DOI:** 10.1126/sciadv.adg7417

**Published:** 2023-07-14

**Authors:** Bozhong Shi, Xiaoyang Zhang, Zhiying Song, Zihao Dai, Kai Luo, Bo Chen, Zijie Zhou, Yue Cui, Bei Feng, Zhongqun Zhu, Jinghao Zheng, Hao Zhang, Xiaomin He

**Affiliations:** ^1^ Department of Cardiothoracic Surgery, Shanghai Children’s Medical Center Affiliated to Shanghai Jiao Tong University School of Medicine, 1678 Dongfang Road, Shanghai 200127, China.; ^2^ Department of Pediatric Surgery, Children’s Hospital of Fudan University, 399 Wanyuan Road, Shanghai 201102, China.; ^3^ Heart Center and Shanghai Institute of Pediatric Congenital Heart Disease, Shanghai Children’s Medical Center, National Children’s Medical Center, Shanghai Jiaotong University School of Medicine; 1678 Dongfang Road, Shanghai 200127, China.

## Abstract

Pressure-overloaded left ventricular remodeling in young population is progressive and readily degenerate into heart failure. The aims of this study were to identify a plasma metabolite that predicts and is mechanistically linked to the disease. Untargeted metabolomics determined elevated plasma kynurenine (Kyn) in both the patient cohorts and the mice model, which was correlated with remodeling parameters. In vitro and in vivo evidence, combined with single-nucleus RNA sequencing (snRNA-seq), demonstrated that Kyn affected both cardiomyocytes and cardiac fibroblasts by activating aryl hydrocarbon receptors (AHR) to up-regulate hypertrophy- and fibrosis-related genes. Shotgun metagenomics and fecal microbiota transplantation revealed the existence of the altered gut microbiota-Kyn relationship. Supplementation of selected microbes reconstructed the gut microbiota, reduced plasma Kyn, and alleviated ventricular remodeling. Our data collectively discovered a gut microbiota–derived metabolite to activate AHR and its gene targets in remodeling young heart, a process that could be prevented by specific gut microbiota modulation.

## Teaser

Gut microbiota-derived plasma kynurenine predicts and promotes remodeling in young heart.

## INTRODUCTION

Ventricular remodeling is a type of cardiac maladaptation that occurs as a consequence of various cardiovascular etiologies. In the absence of timely intervention, ventricular remodeling gradually leads to compromised cardiac function and eventual heart failure (*1*). In pressure-overloaded left ventricular (poLV) diseases, especially in congenital heart malformations, ventricular remodeling may present prenatally and becomes persistent, burdensome, and progressive until surgical correction (*2*). Late intervention results in irreversible myocardial hypertrophy and interstitial fibrosis, which greatly increase the risks of heart failure and surgical mortality and worsen long-term outcomes (*3*–*6*). However, the optimal timing for intervention is often difficult to determine because currently available indicators depend mainly on gradient progression and/or the onset of symptoms, which do not indicate the degree of internal damage and functional deterioration at the early stages. Therefore, sensitive biomarkers and effective therapeutic modalities are urgently required to detect and prevent heart failure and improve prognosis.

Numerous pathological, metabolic, and molecular changes associated with ventricular remodeling have been elucidated at heart level (*7*), while the recent emergence of multi-omics methods has enabled us to revisit cardiac disease in a more systematic way. Heart failure is now recognized as a systemic process involving coordinated networks of different organs and multifactorial mechanisms. In addition to the circulatory system, heart failure affects other organ systems through distant signaling. The latter may itself exacerbate the disease. Metabolomics has disclosed a vast repertoire of plasma metabolites related to cardiovascular diseases (CVDs) (*8*–*11*). These metabolites may either serve as biomarkers or elicit their functions through cell receptors and signaling cascades. This systemic disease model may be especially important in children, as their organs are comparatively more sensitive to circulating stimuli than those of adults (*12*). Prior research has demonstrated gut hypoperfusion in multiple CVDs (*13*–*15*), followed by the alterations of the gut microbiota and its metabolite leakage to promote the disease progression (*16*, *17*). However, the interkingdom interaction between gut microbiota and poLV remodeling is less defined.

In the present study, a multi-omics study was conducted on pediatric human samples and a mouse model to discover a plasma metabolite linked to ventricular remodeling in young heart and shed light on its underlying mechanism via gut microbiota–heart communication. Furthermore, the cardio-protective effect of supplementation of specific microbes, targeting the altered gut microbiota, was evaluated.

## RESULTS

### Plasma kynurenine is elevated and associated with remodeling in poLV children and mice

**Fig. 1.**
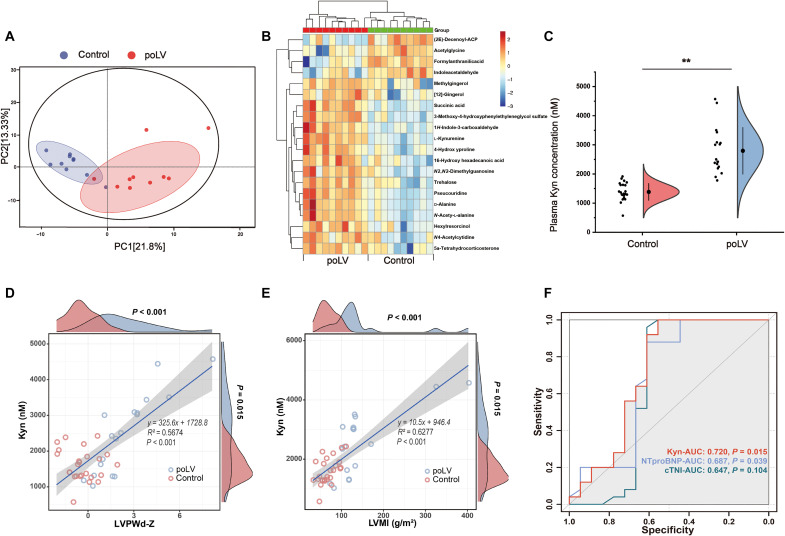
Untargeted metabolomics identifies elevation of plasma Kyn in poLV children. (**A**) Untargeted metabolomics was performed in a pilot discovery cohort (*n* = 20). PCA showed a clear separation of metabolite component between the two groups. (**B**) The heatmap of differential plasma metabolites with top significance. (**C**) UHPC-MS/MS was performed to quantify plasma Kyn concentration in a nonoverlapping validation cohort (*n* = 19 in poLV group and *n* = 25 in control group). Unpaired Student’s *t* test was used. (**D** and **E**) Linear regression showed correlation between Kyn and LVPWd *Z* score (D) or LVMI (E). (**F**) AUC showed diagnosis efficacy of Kyn to discriminate two groups, comparing to traditional blood biomarkers NT-proBNP and cTNI. Each data point represents the mean of an individual patient. **P < 0.01.

To seek plasma biomarkers of poLV remodeling in children, we recruited an age-matched pilot discovery cohort enrolling pediatric patients diagnosed with LV outflow tract obstruction (LVOTO) diseases (poLV group; *n* = 10). Those with small atrial/ventricular septum defect served as controls (*n* = 10; table S1). We then conducted untargeted metabolomics between their blood plasma. Principal components analysis (PCA) disclosed substantial difference in plasma metabolites between groups (Fig. 1A). Numerous metabolites significantly differed (adjusted *P <* 0.05; variable importance in projection > 1; Fig. 1B and table S2). A primary analysis of the top metabolites with clinical data demonstrated that kynurenine (Kyn) was the most correlated with cardiac remodeling parameters, including LV posterior wall thickness at end-diastole (LVPWd) *Z* score, LV mass index (LVMI), relative wall thickness (RWT), and ejection fraction (EF%; fig. S1A). To quantify the plasma Kyn concentrations, we conducted ultrahigh performance liquid chromatography–tandem mass spectrometry (UHPC-MS/MS) using an independent nonoverlapping validation cohort (*n* = 19 in poLV group versus 25 in control group; table S3). The Kyn levels were comparatively higher in the poLV group (Fig. 1C). There were also significant correlations between the Kyn level and LVPWd *Z* score (*R*^2^ = 0.5674, *P <* 0.001), LVMI (*R*^2^ = 0.6277, *P <* 0.001), RWT (*R*^2^ = 0.3121, *P <* 0.001), and EF% (*R*^2^ = 0.5726, *P <* 0.001; Fig. 1, D and E, and fig. S1, B and C). The cutoff value for Kyn is 2421.39 nM. Comparing to traditional blood indices, including classic cardiac function parameter N-terminal pro–B-type natriuretic peptide (NT-proBNP), and myocardium injury parameter cardiac troponin I (cTNI), the receiver operating characteristic curve of Kyn in was higher [area under the curve (AUC) = 0.720; Fig. 1F]. Hence, Kyn might more effectively discriminate remodeling patients not presenting with declining cardiac function or severe injury, showing great potential in the early prediction of poLV remodeling and might have a role in disease progression.

To assess whether Kyn is mechanistically linked to pressure-overload induced remodeling, we established a neonatal mouse ascending aorta constriction (nAAC) model and simulated poLV in young mice (movie S1). Echocardiography was first used to measure the diameters of the ascending aortae in newborn mice. It revealed that 28-, 30-, and 32-gauge needles induced 50 to 70% constriction within 1 to 3 days after birth (fig. S2A). The 28-gauge needle constriction was selected to increase survival, prolong lifespan, and study chronic remodeling from birth to pre-adult. The ascending aorta was surgically constricted in newborn mice within 24 hours after birth, and the model was evaluated at different time points (fig. S2B). The efficacy of nAAC model was assessed by echocardiology at postnatal day 28 (P28) and gross evaluation and multiple histological staining at P7 to P28 (figs. S2, C to E, and S3 to S5). Myocardium hypertrophy could be obvious at P14, and fibrosis became significant at P21. To verify the results for the patient cohort, untargeted metabolomics was again conducted between nAAC and sham mice (*n* = 10, respectively) at 4 weeks of age. This time point represents both the young stage and the chronic pathology covering the age range of the patient cohort. PCA results indicated notable differences between sham and nAAC mice plasma metabolites (fig. S6A). Kyn was among the most highly elevated metabolites in nAAC plasma (fig. S6B and table S4). Kyn is a major intermediate bioactive catabolite of tryptophan metabolism, which was among the most enriched pathways (fig. S6C). Phenylalanine, tyrosine, and tryptophan biosynthesis and the phenylalanine metabolism pathway were also enriched, while other Kyn relating pathways and their metabolites were less or not significant (table S4). UHPC-MS/MS quantified excessive Kyn in 51 nAAC mice versus 29 sham mice (fig. S7A). There were also significant correlations between the mice Kyn level and LVEF% (*R*^2^ = 0.7676, *P* < 0.001), left ventricular diameter at end-diastole (LVDd; *R*^2^ = 0.4623, *P* 0.001), and LV mass (*R*^2^ = 0.5114, *P* 0.001; fig. S7, B to D), which substantiated the findings in human cohorts.

### Excessive plasma Kyn activates AHR in cardiomyocytes and cardiac fibroblasts

**Fig. 2.**
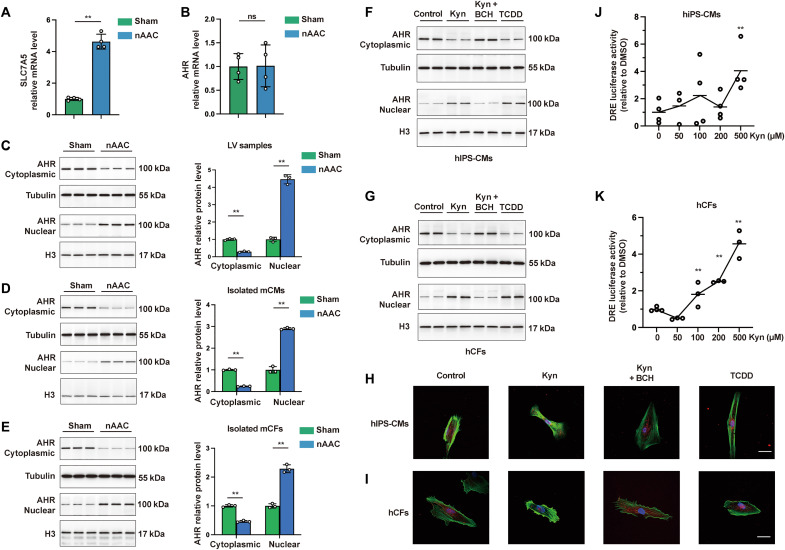
Excessive plasma Kyn activates AHR in CMs and CFs in poLV. (**A**) Quantitative polymerase chain reaction (qPCR) quantification of the Kyn transporter SLC7A5 between sham and nAAC LV samples. *n* = 4 per group. (**B**) mRNA level of *AHR* between sham and nAAC LV samples. *n* = 4 per group. (**C** to **E**) Western blotting (left) and quantification (right) of AHR protein in the cytoplasm and nucleus of LV samples (C), isolated mCMs (D), and isolated mice mCFs (E) from sham and nAAC mice. *n* = 3 per group. Each lane represented one animal. (**F** and **G**) Representative Western blotting of AHR in the cytoplasm and nucleus of hIPS-CMs (F) and hCFs (G), after 48 hours of treatment with 500 μM Kyn, 500 μM Kyn and 500 μM Kyn transporter inhibitor BCH, 500 μM AHR activator TCDD as positive control, or the same amount of dimethyl sulfoxide (DMSO) as negative control. Each lane represented individual experiment. (**H** and **I**) Representative immunofluorescence staining of AHR (red) in hIPS-CMs (H) and hCFs (I) treated by indicated compounds for 48 hours, with phalloidin staining (green) the F-actin in cytoplasm and 4′,6-diamidino-2-phenylindole (DAPI; blue) staining the nucleus. Scale bars, 25 μm. (**J** and **K**) DRE luciferase activity in hIPS-CMs (J) and hCF (K) treated with different dose of Kyn after 24 hours, each comparing to the baseline. *n* = 3 to 4 per group. Representative images from individual experiment in vitro [(F) to (I); *n* = 3 in each group]. Unpaired *t* test was used in (A) to (E). One-way analysis of variance (ANOVA) followed by the Bonferroni post hoc analysis was used in (J) and (K). ns, not significant; **P < 0.01.

Kyn is a strong endogenous ligand that activates aryl hydrocarbon receptors (AHRs) in tumor and immune cells (*18*–*20*). However, its mechanisms and functions in heart were diverse. We found that the Kyn membrane transporter SLC7A5 was transcriptionally up-regulated in LV samples of 4-week-old nAAC mice (Fig. 2A), suggesting a higher uptake of Kyn. Consistent with other studies (*19*, *21*, *22*), we detected no transcriptional differences in *AHR* between the sham and nAAC LV tissues (Fig. 2B), indicating that its activation was not in the mode of up/down-regulation in nAAC heart. However, AHR protein expression was decreased in the cytoplasm and increased in the nucleus in nAAC LV (Fig. 2C). Thus, AHR was here activated via nuclear translocation.

CM hypertrophy and interstitial fibrosis are principal poLV remodeling processes, which were reported to act independently (*23*). To this end, we isolated mouse cardiac fibroblasts (mCFs) and cardiomyocytes (mCMs) in 4-week mice models to identify which cell types dominate this process. AHR nuclear translocation was verified in both nAAC mCMs and mCFs (Fig. 2, D and E). To further confirm this result in vitro, we cultured human CFs (hCFs) and human induced pluripotent stem cell–derived CMs (hIPS-CMs) that allows us to study human CMs at young, immature stages. Adding Kyn induced decreased cytoplasmic and increased nuclear AHR in both cell types, which was inhibited by adding the Kyn transporter inhibitor 2-aminobicyclo [2.2.1] heptane-2-carboxylic acid (BCH). The synthetic xenobiotic 2,3,7,8-tetrachlorodibenzo-*p*-dioxin (TCDD) directly bound AHR was used as a positive control (Fig. 2, F and G). A paralleled immunofluorescence of AHR protein also confirmed the results (Fig. 2, H and I). Nuclear AHR action was initiated by interacting with the core-binding motifs of the dioxin-responsive elements (DREs) in the target gene regulatory regions (*24*). Compared with the baseline, the DRE luciferase activity was higher in the hIPS-CMs and hCFs exposed to Kyn with stable significance at 500 μM (Fig. 2, J and K), suggesting that the nuclear AHR could regulate its target genes after Kyn activates it.

### Kyn-AHR aggravates hypertrophy and fibrosis in poLV

**Fig. 3.**
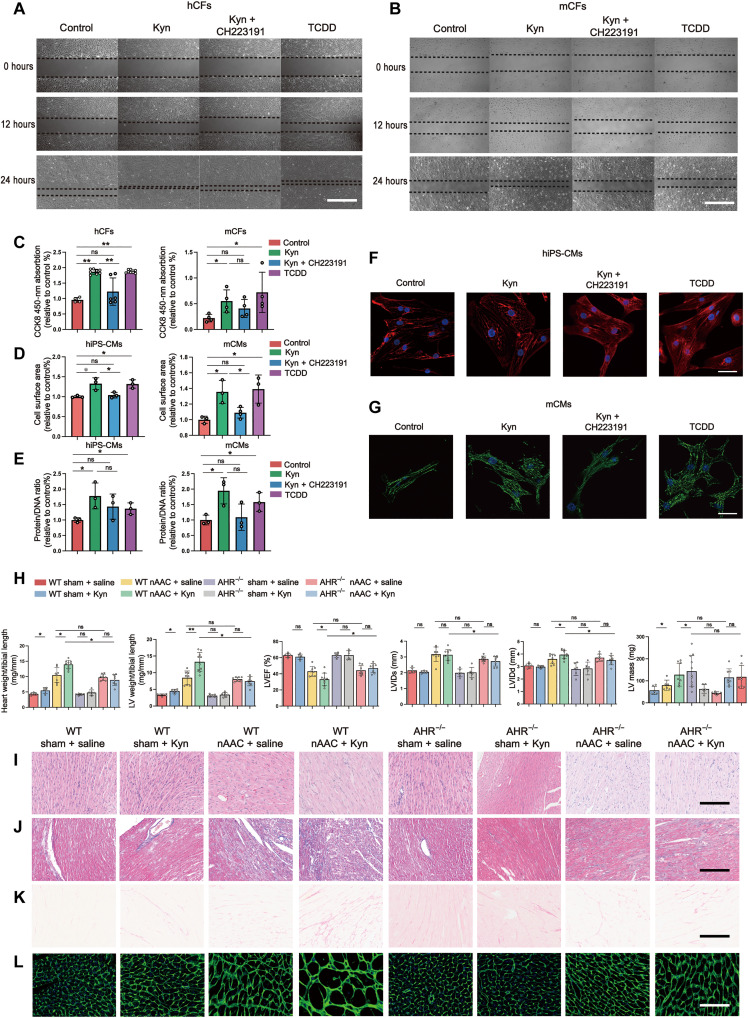
Increased Kyn and AHR activation aggravate cardiac remodeling. (**A** and **B**) Representative scratch assay of hCFs (A) and mCFs (B) treated with 500 μM Kyn, 500 μM Kyn and 500 μM AHR inhibitor CH223191, 500 μM AHR activator TCDD as positive control, or the same amount of DMSO as negative control. Scale bars, 200 μm. (**C**) CCK8 activity to detect cell viability in hCFs and mCFs after 48 hours of above treatment. *n* = 4 to 8 per group. (**D**) Cell surface area of hIPS-CMs and mCMs measured under bright fields after 48 hours of treatment with the indicated compounds. *n* = 3 to 4 per group. (**E**) Protein/DNA ratio of hIPS-CMs and mCMs after 48 hours of treatment with the indicated compounds. *n* = 3 per group. (**F** and **G**) Representative immunofluorescence staining of hIPS-CMs (F) and mCMs (G) to illustrate the size of cells. cTNI in hIPS-CMs (red) and actinin in mCMs (green) were used to stain the cell skeleton, while DAPI (blue) was used to show nucleus. Scale bars, 10 μm. (**H**) Evaluation of cardiac remodeling and cardiac function in WT and *AHR*^−/−^ mice 4 weeks after sham or nAAC surgery, with Kyn peritoneal injection (50 mg/kg daily) or saline as control. The assessments included heart weight/tibial length and LV weight/tibial length at gross level and LVEF%, left ventricular diameter at end-diastole (LVIDd), left ventricular diameter at end-systole (LVIDs), and estimated LV mass by echocardiography (from left to right). *n* = 6 to 10 per group. (**I** to **K**) Representative histological staining of LV samples in the corresponding groups. H&E (I) staining illustrated the cell size, while Masson staining (J) and Sirius red staining (K) showed the degree of fibrosis. Scale bars, 100 μm. (**L**) WGA fluorescence staining showed the cell boundaries of CMs, indicating the degree of hypertrophy. Scale bars, 50 μm. Representative images from individual experiment in vitro [(A), (B), (F), and (G), *n* = 3 in each group] and individual animal [(I) to (L), *n* = 5 in each group]. Kruskal-Wallis test and post hoc Dunn test were used in (C) to (E) and (H). *P < 0.05 and **P < 0.01.

We conducted an in vitro study to verify the phenotypes induced by Kyn and AHR activation. Scratch and Cell counting kit-8 (CCK8) assays revealed that Kyn treatment promoted hCF and mCF proliferation and viability. These effects were ameliorated by adding the AHR antagonist CH223191 (Fig. 3, A to C). Kyn induced hypertrophy in hIPS-CMs and mCMs by increasing their cell surface areas (Fig. 3, D, F, and G) and protein/DNA ratios (Fig. 3E). Both of these were rescued by CH223191 treatment.

For in vivo study, we developed and bred *AHR* knockout (*AHR*^−/−^) mice. The sham and nAAC mice were administered daily peritoneal injections of Kyn (50 mg/kg) for 4 weeks. In the wild-type (WT) sham mice, the Kyn injections only increased the heart and LV weights but did not alter cardiac function (Fig. 3H) or histological structures (Fig. 3, I to L, and fig. S9, A to C). This may represent physiological hypertrophy because markers associated with cardiac stress and pathological hypertrophy (*ANP*, *BNP*, and *b-MHC*) and fibrosis (CTGF) did not increase (fig. S9, D to G). Kyn failed to elicit detectable changes in the *AHR*^−/−^ sham mice. In the WT nAAC mice, Kyn supplementation significantly accelerated remodeling in gross evaluation, echocardiography (Fig. 3H), and histological stainings, including hematoxylin and eosin (H&E), Masson’s trichrome, and Sirius Red staining and wheat germ agglutinin (WGA) fluorescence staining (Fig. 3, I to L, and fig. S9, A to C). However, *AHR*^−/−^ knockout aborted the aggravative effect of Kyn in the nAAC mice. Therefore, the AHR receptor is essential for Kyn to elicit its function.

### Kyn-AHR axis targets the downstream hypertrophy and fibrosis genes in poLV

**Fig. 4.**
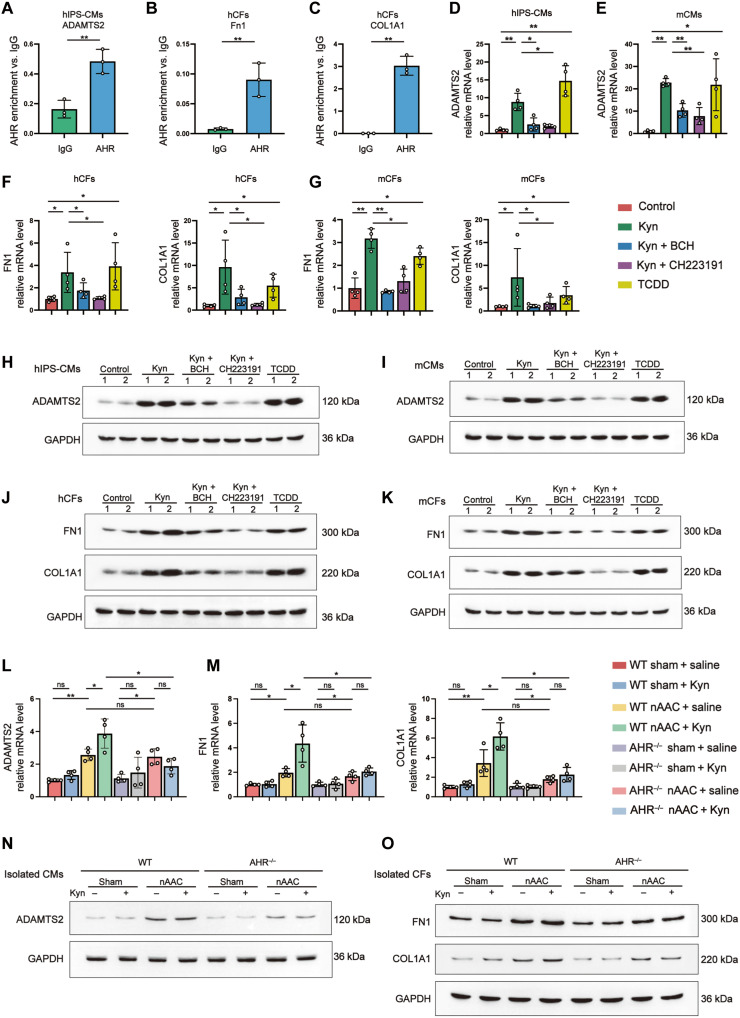
Kyn-AHR activation initiated the expression of hypertrophy and fibrosis genes. (**A** to **C**) AHR enrichment in comparing to immunoglobulin G in chromatin immunoprecipitation of *ADAMTS2* in hIPS-CMs (A), *FN1* (B), and *COL1A1* (C) in hCFs. *n* = 3 per group. (**D** and **E**) qPCR quantified mRNA level of *ADAMTS2* in hIPS-CMs (D) and mCMs (E), treated for 48 hours with 500 μM Kyn, 500 μM Kyn and 500 μM BCH, 500 μM Kyn and 500 μM AHR inhibitor CH223191, 500 μM TCDD as positive control, or the same amount of DMSO as negative control. (**F** and **G**) mRNA level of *FN1* and *COL1A1* in hCFs (F) and mCFs (G) treated with indicated compounds. (**H** and **I**) Representative Western blotting of the protein expression ADAMTS2 in hIPS-CMs (H) and mCMs (I) treated with indicated compounds. Each lane represented individual experiment in vitro. (**J** and **K**) Representative Western blotting of FN1 and COL1A1 in hCFs (J) and mCFs (K) treated with indicated compounds. Each lane represented individual experiment in vitro. (**L** and **M**) qPCR quantified mRNA level of *ADAMTS2* in isolated mCMs (L) and *FN1* and *COL1A1* in isolated mCFs (M) from WT and *AHR*^−/−^ mice 4 weeks after sham or nAAC surgery, with Kyn peritoneal injection (50 mg/kg) or saline as control. (**N** and **O**) Representative Western blotting of the protein expression of ADAMTS2 in isolated mCMs (N) and FN1 and COL1A1 in isolated mCFs (O) from indicated mice. Each lane represented individual animal. Two-tailed Mann-Whitney *U* test was used in (A) to (C). Kruskal-Wallis test and post hoc Dunn test were used in (D) to (G), (L), and (M). *P < 0.05 and **P < 0.01.

To identify the downstream AHR targets in poLV, whole-transcriptome sequencing was performed on LV samples from 4-week-old sham (*n* = 5) and nAAC (*n* = 5) mice (fig. S10A). Consistent with the preceding findings, transcriptive AHR was unaffected and was not detected among the differentially expressed genes (DEGs) [adjusted *P <* 0.05 and log_2_ fold change (FC) > 1]. Ingenuity pathway analysis (IPA) was used to screen DEGs regulated by AHR (fig. S10B). Most DEGs were associated with fibrosis and included *COL1A1*, *COL1A2*, *COL3A1*, and *FN1*. The roles of *COL1A1* and *FN1* in heart failure were previously well explored (*25*, *26*). We also detected the DEG *ADAMTS2*, which was previously found to participate in cardiac hypertrophy (*27*, *28*). Plasma untargeted metabolomics and RNA sequencing of the same mice revealed strong correlations between the Kyn and *COL1A1*, *FN1*, and *ADAMTS2* expression levels (fig. S10C). Chromatin immunoprecipitation was used to verify the interactions between AHR and *ADAMTS2* in Kyn-preconditioned hIPS-CMs (Fig. 4A), as well those between AHR and *FN1* and between AHR and *COL1A1* in Kyn-preconditioned hCFs (Fig. 4, B and C).

We subjected hIPS-CMs, mCMs, hCFs, and mCFs with Kyn and the aforementioned compounds to illustrate the Kyn-AHR remodeling axis in vitro. *ADAMTS2* in hIPS-CMs (Fig. 4, D and H, and fig. S11, A and E) and mCMs (Fig. 4, E and I, and fig. S11, B and F) and *COL1A1* and *FN1* in hCFs (Fig. 4, F and J, and fig. S11, C, G, and I) and mCFs (Fig. 4, G and K, and fig. S11, D, H, and J) were up-regulated at the transcription and translation levels after treatment with Kyn or TCDD. These effects were rescued with the uptake inhibitor BCH or the AHR inhibitor CH223191.

In vivo, AHR-targeted genes were evaluated in mCMs or mCFs isolated from LV tissues of 4-week-old WT or *AHR*^−/−^ mice exposed to aforementioned treatments. *ADAMTS2* was up-regulated in the mCMs isolated from nAAC mice. Kyn injection significantly up-regulated *ADAMTS2* in WT nAAC mice, whereas this effect was abolished in *AHR*^−/−^ mice (Fig. 4, L and N, and fig. S12, A and C). Similar results were obtained for *COL1A1* and *FN1* in isolated CFs (Fig. 4, M and O, and fig. S12, D to G). The results of the foregoing in vitro and in vivo experiments indicated that the Kyn-AHR axis up-regulated the fibrosis- and hypertrophy-related genes in murine poLV remodeling.

### Single-nucleus RNA sequencing in pediatric poLV samples confirmed the Kyn-AHR axis

**Fig. 5.**
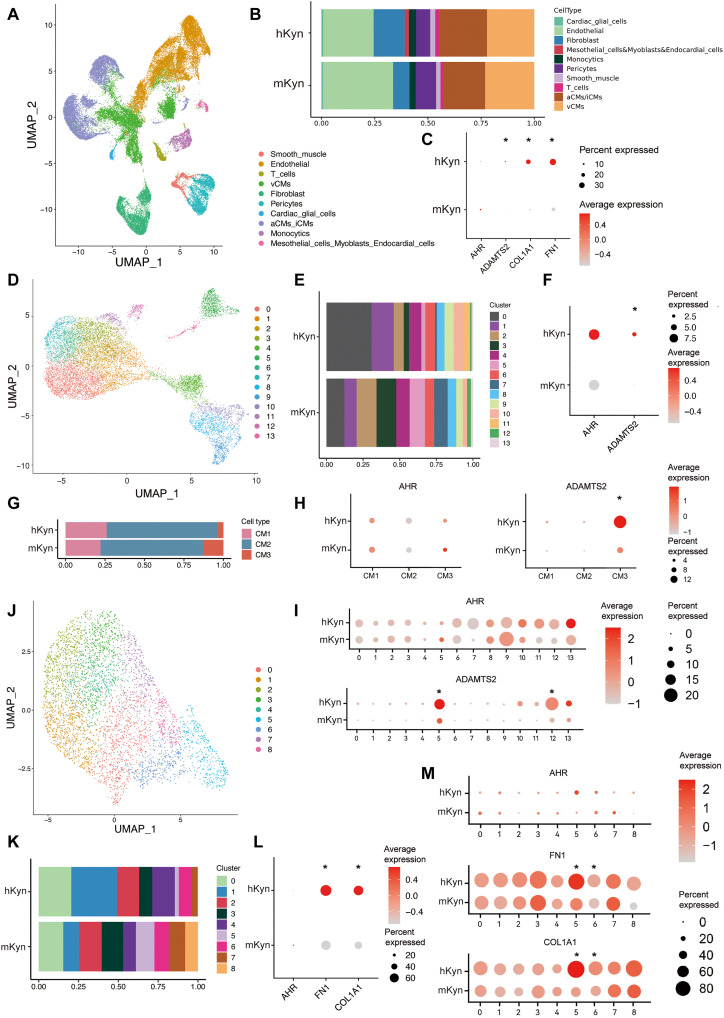
Single-nucleus RNA sequencing of LV samples from pediatric poLV patients confirms Kyn-AHR effect. (**A**) Uniform manifold approximation and projection (UMAP) of the four LV samples in single-nucleus RNA sequencing. Cells were marked by cell types. (**B**) Cell proportion of different types in two groups. (**C**) *AHR*, *ADAMTS2*, *COLA1*, and *FN1* expression of the LV samples between the two groups. (**D**) UMAP of the subcluster of CMs. (**E**) Cell proportion of CM subclusters between groups. (**F**) *AHR* and *ADAMTS2* expression in total CMs between groups. (**G**) Cell proportion of CM types between groups. (**H** and **I**) *AHR* and *ADAMTS2* expression in different CM types (H) and clusters (I) between groups. (**J**) UMAP of the subcluster of CFs. (**K**) Cell proportion of CF subclusters between groups. (**L** and **M**) *AHR*, *FN1*, and *COL1A1* expression in total CFs (L) and subclusters (M) between groups. Significant differential expression was marked with “*” and under criteria of (i) lnFC > 0.25, (ii) *P* < 0.05, and (iii) min.pct > 0.1.

Single-cell RNA sequencing is ideal for the analysis of heart diseases attributed to various types of cells. Hence, we used this tool to validate our findings in the human poLV heart. LV tissues were collected from four patients with LVOTO requiring surgical resection. On the basis of their plasma Kyn levels, they were classified into mild elevated Kyn group (mKyn, *n* = 2; Kyn < cutoff value) and the highly elevated Kyn (hKyn, *n* = 2; Kyn > cutoff value) groups (table S5). Transcriptomes were obtained for 40,986 cells, filtered, and converged into a single data pool comprising 39,308 cells (table S5). A Seurat cluster analysis defined 10 different cell types (Fig. 5A and fig. S13A), where the proportion of CFs was substantially greater in the hKyn group (Fig. 5B). Therefore, Kyn might contribute to fibrotic tissue overgrowth in humans. *AHR* distributed in the majority of the cell types and most highly expressed in cardiac glial cells (fig. S13B). *AHR* did not markedly differ between groups. However, its target genes *ADAMTS2*, *FN1*, and *COL1A1* were up-regulated in the hKyn group (Fig. 5C).

We partitioned the CMs into 14 subclusters and defined three types (CM1 to CM3) based on their transcriptomes (Fig. 5D and fig. S13C). CM1 included canonical ventricular CMs, and CM2 presented characteristic of immature or atrial CMs. CM3 displayed endothelial-derived fibroblast properties that participate in remodeling processes such as angiogenesis and extracellular matrix (ECM) organization (fig. S13D). The hKyn group was abundant in subclusters 3, 5, 7, and 12, mainly classified into CM2 and CM3 (Fig. 5, E and G). For this reason, high Kyn may contribute to CM immaturity and remodeling process. *AHR* did not differ among total CMs, subclusters, or types. The AHR downstream remodeling gene *ADAMTS2* was up-regulated in the total CMs, subclusters 5 and 12, and CM3 (Fig. 5, F, H, and I). Subclusters 5 and 12 participated in multiple remodeling processes (fig. S13, E and F). These finding were consistent with the Kyn-AHR mechanism.

The CFs were categorized into nine subclusters (0 to 8; Fig. 5J and fig. S14A). A proportion analysis revealed that subclusters 1 and 2 predominated in the mKyn group, whereas the hKyn group was characterized by subclusters 5, 7, and 8 (Fig. 5K). These observations were corroborated by a pseudo-time analysis illustrating the cell trace of transition, where the predominance of subclusters 1 and 2 gradually transitioned to subclusters 5, 7, and 8 in response to high Kyn stimuli (fig. S14, B and C). *AHR* expression did not change in the total CFs or any subclusters. *FN1* and *COL1A1* were up-regulated in the total CFs and subclusters 5 and 6 in the hKyn group (Fig. 5, L and M). Subclusters 5 and 6 participated mainly in remodeling processes such as cell adhesion and ECM organization and angiogenesis, as well as muscle contraction processes closely related to cardiac function (fig. S14, D and E). Therefore, the results of the small nuclear RNA (snRNA) assays on the human samples refined and validated our experimental findings.

To verify the above snRNA sequencing (snRNA-seq) results of poLV Kyn-AHR on cardiac immaturity (proliferation and regeneration) and angiogenesis, we performed immunofluorescence staining of Ki67 (proliferation), Aurora B (cytokinesis), PH3 (mitosis) with cardiac Troponin T (cTNT), and vascular endothelial growth factor and CD31 with WGA of P7 mice LV samples (fig. S15). nAAC mice presented with higher rates of myocyte regeneration and angiogenesis than those of the sham mice. The injection of Kyn significantly promoted regeneration and angiogenesis, while knockout of *AHR* abolished those effects. Moreover, because Kyn had a classic role in immune modulation (*18*, *20*) and none of the above subclusters of CMs or CFs were enriched in immune process, we further performed CD4 immunostaining and quantification of T cell in 4-week-old nAAC mice. Kyn injection slightly increased T cell without statistical significance (fig. S16A). The snRNA-seq result also showed no significant difference in the composition of naive T cell (T_N_) and recently activated effector memory or effector T cells (T_EMRA_/T_EFF_; fig. S16B). The above findings validated the snRNA-seq results in mice models.

### Gut microbiota alteration in poLV disease contributes to plasma Kyn accumulation

**Fig. 6.**
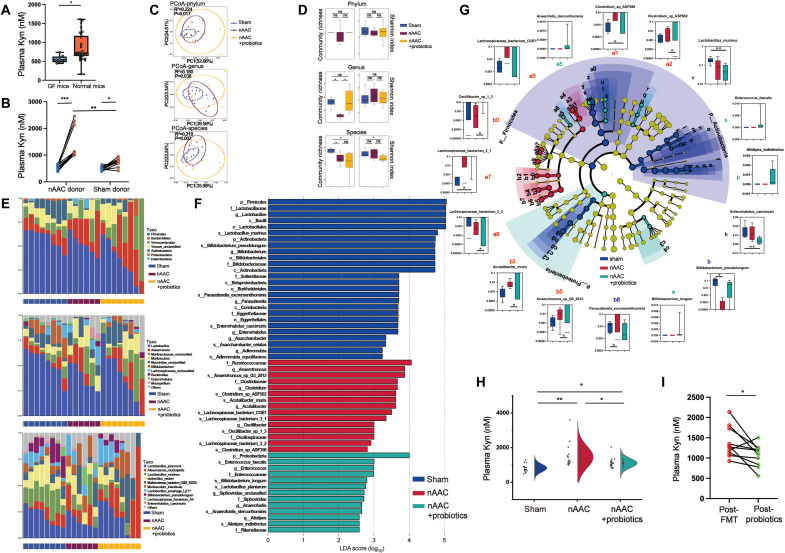
Probiotics reconstructs gut microbiota of nAAC mice and reduces plasma Kyn. (**A**) UHPC-MS/MS quantification of baseline Kyn concentration between the GF mice (*n* = 10) and C57BL/6 mice (*n* = 39). (**B**) UHPC-MS/MS quantification of plasma Kyn concentration in GF mice before (pre-FMT) and after (post-FMT) 2 weeks of FMT from nAAC or sham donor mice (*n* = 10). (**C**) PCoA by Bray-Curtis distance at phylum (*R*^2^ = 0.224, *P* = 0.017), genus (*R*^2^ = 0.180, *P* = 0.038), and species level (*R*^2^ = 0.218, *P* = 0.002) in metagenomics sequencing among the feces of sham (*n* = 8), nAAC (*n* = 6), and nAAC mice with probiotics (*n* = 8). Permutational multivariate ANOVA test was used. (**D**) Community richness and homogeneity (Shannon index) at genus, phylum, and species level among the three groups. (**E**) The relative abundance of top 10 microbes at phylum (top), genus (middle), and species (bottom). (**F**) Linear discriminate analysis effect size (LEfSe) to illustrate the specific microbes with top abundance that characterized each group. (**G**) Cladogram of the characterized microbes in each group, showing their evolutional and familial relationship (middle). Kruskal-Wallis rank sum tests (outer circle) were also conducted to demonstrate differential abundance of the specific microbes. (**H**) UHPC-MS/MS quantification of plasma Kyn concentration among 4-week-old mice with sham (*n* = 15), nAAC surgery (*n* = 19), and nAAC and probiotics supplement (*n* = 20). (**I**) UHPC-MS/MS quantification of plasma Kyn between GF mice with 2 weeks of FMT from nAAC donor mice (post-FMT) and after another 2 weeks of probiotics gavage (post-probiotics), *n* = 10. Two-tailed Mann-Whitney *U* test was used in (A), (B), and (I). Kruskal-Wallis test and post hoc Dunn test were used in (G). One-way ANOVA followed by the Bonferroni post hoc analysis were used for (H). *P < 0.05 and **P < 0.01.

Both Kyn and tryptophan metabolism are closely associated with the gut microbiota (*29*). Therefore, we suspected that the latter explained the excessively high plasma Kyn levels in poLV. Clinical cohorts and mouse models already demonstrated that heart failure induces hypoperfusion and structural interruption (*14*, *15*), followed by alteration of the gut microbiota. Nevertheless, this mechanism is poorly defined in pediatric poLV. Ultrasonography detected a decrease blood flow in both abdominal aortae and mesentery artery in nAAC mice, compared to those of sham mice (fig. S17). H&E staining disclosed the structural changes in the colons of nAAC mice including mucosal atrophy, interrupted epithelium, and sparse intestinal villi (fig. S18A). We applied 16*S* RNA sequencing to identify gut dysbiosis in 4-week-old nAAC mice (*n* = 6 versus 6). Beta-diversity and a principal coordinate analysis (PCoA) by Bray-Curtis distance revealed significant alterations in the nAAC gut microbiota (fig. S18B). Shannon and Simpson indices showed that the alpha diversity of the nAAC gut microbiota had significantly decreased (fig. S18, C and D). A lower Firmicutes/Bacteroidetes ratio, associated with inflammation, was observed in the nAAC group (fig. S18E). The mRNA expression of *IDO*/*TDO* ratio, the key enzymes in the degradation of tryptophan to produce Kyn, was higher in nAAC colon (fig. S18F), suggesting the overproduction of Kyn in nAAC gut. The integrity of intestinal barrier was also tested by *ZO-1*, *Occludin*, and *Cdh1* level, where *Occludin* was significantly lower in nAAC colon (fig. S18G). We then used a germ-free (GF) mouse model to validate Kyn-gut microbiota hypothesis and exclude any other variation. The baseline plasma Kyn was lower in the GF mice than the C57BL/6 mice (age, 4 weeks, *n* = 10 versus 39), suggesting that gut microbiota were involved in physiological production of Kyn (Fig. 6A). We administered nAAC and sham fecal microbiota transplantation (FMT) to age- and gender-matched GF mice and elucidated the relationships between gut microbiota and Kyn (fig. S19). While there was also an increase of Kyn after FMT of the sham donors, the Kyn levels were significantly higher in the plasma of GF mice transplanted with nAAC gut microbiota (Fig. 6B). The above data indicated the existence of the relationship between nAAC gut microbiota and excessive plasma Kyn.

### Supplementation of selected probiotics reconstructs the gut microbiota and reduces plasma Kyn in nAAC mice

We identified plasma Kyn as a pathological signal between the gut microbiota and poLV remodeling and then sought an efficacious potential treatment to block this process. Reshaping the gut microbiota has demonstrated efficacy in ameliorating diabetes and hypertension in adult humans (*30*, *31*). The preceding 16*S* results revealed a decreased abundance of several microbes at genus level in nAAC mice (fig. S20A), where three microbes *Bifidobacterium*, *Lactobacillus*, and *Enterococcus* have aroused our interests because specific species under these genus have been reported to have potential protective role in CVDs (*32*). Given that, we constructed three stains (*Bifidobacterium longum 913*, *Lactobacillus acidophilus 145*, and *Enterococcus faecalis ATCC19433*) into a triple-probiotics suspension with more than 1.0 × 10^7^ colony-forming units of each microbes and initiated daily gavage administration to nAAC mice. The efficacy was firstly confirmed by a pilot quantitative reverse transcription polymerase chain reaction study of the colonic contents, where all three bacterial species reached stable colonization by day 10 (fig. S20, B to D).

To characterize the effects of the probiotics suspension, we performed shotgun metagenomics analysis on the 4-week-old sham mice (*n* = 8), nAAC mice (*n* = 6), and nAAC mice administered probiotics (*n* = 8). A PCoA by Bray-Curtis distance distinguished the gut microbiota composition between the sham and nAAC mice groups at the phylum, genus, and species levels (fig. S21A) and was reshaped by the probiotics (Fig. 6C). Genus- and species-level community richness was lower in the nAAC than the sham mice and was restored at genus level to the normal range by the probiotics (Fig. 6D). The Shannon index at genus and species level showed a slightly higher trend in the nAAC mice but without statistical significance (*P* > 0.05). However, it was significantly mitigated by the probiotics treatment (*P* = 0.038; Fig. 6D). The relative abundances of the top 10 microbes are shown in Fig. 6E. Linear discriminate analysis effect size analysis identified the specific microbial taxa characterizing each treatment group (Fig. 6F). A detailed evolutionary cladogram was plotted, and a Kruskal-Wallis rank sum test was run to demonstrate differential microbial abundances (Fig. 6G). The sham group had high abundances of the bacterial genera *Lactobacillus*, *Bifidobacterium*, *Parasutterella*, and *Enterorhabdus*, consistent with 16*S* results. By contrast, *Clostridium* and *Acutalibacter* predominated in the nAAC group. The phyla Firmicutes, Proteobacteria, and Actinobacteria were abundant in the sham group but virtually absent in the nAAC group. The probiotics treatment restored Proteobacteria. The sham group had high abundances of *Lactobacillus murinus*, *Enterorhabdus caecimuris*, *Parasutterella excrementihominis*, and *Bifidobacterium pseudolongum*. In the nAAC group, the foregoing taxa were nearly absent and were replaced by *Anaerotruncus_sp_G3_2012*, *Clostridium_sp_ASF502/356*, *Acutalibacter muris*, *Lachnospiraceae_bacterium_COE1*/*3_1*/*3_2*, and *Oscillibacter_sp_1_3.* Nevertheless, probiotics supplementation suppressed these taxa to varying degrees. The oral probiotics also introduced the other species *Anaerofutis stercorihiminis* and *Alistipes indistinctus* into the gut microbiota. The above metagenomics results illustrated how specifically the probiotics reshaped the gut microbiota of nAAC. To preliminarily explain why probiotics could reshape the microbiota, the bioinformatic interaction between the microbes was analyzed. The nAAC abundant microbial genera and species were strongly correlated, and their growth was mutually promoted (fig. S21B). Introducing probiotics, especially *Enterorhabdus caecimuris and Lactobacillus murinus*, into this environment might attenuate the interactions among the nAAC microbial taxa.

Probiotics supplementation significantly reduced Kyn in the nAAC mice (Fig. 6H). An additional 2 weeks of probiotics gavage reduced plasma Kyn in the GF mice transplanted with nAAC feces (Fig. 6I). Metagenomics and untargeted metabolomics joint analyses disclosed strong positive correlations among plasma Kyn and *Acutalibacter muris*, *Clostridium*, and Firmicutes. These microbial taxa were abundant in the nAAC mice. *A. indistinctus*, *Anaerofustis stercorihominis*, and *E. faecalis* were abundant in the probiotics treatment group and were negatively correlated with Kyn (fig. S22). Thus, these bacterial species might possibly play a role in reducing plasma Kyn and are worth further mechanism research. The aforementioned omics study demonstrated that probiotics effectively reconstruct the gut microbiota and reduce its Kyn metabolite content.

### Reshaping gut microbiota protects the heart from poLV remodeling by modulating Kyn-AHR–targeted genes

**Fig. 7.**
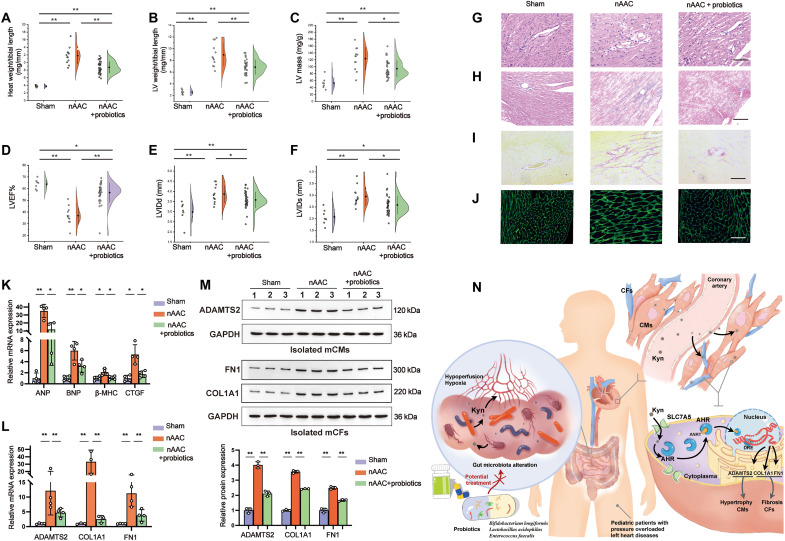
Evaluation of the protective effect of probiotics on poLV remodeling. (**A** and **B**) Gross evaluation of heart weight/tibial length (A) and LV weight/tibial length (B) in 4-week-old sham mice (*n* = 5), nAAC mice (*n* = 10), and nAAC mice supplied with probiotics (*n* = 34). (**C** to **F**) Echocardiography of the cardiac function and structural changes in 4-week-old mice of each group (*n* = 5, 10, and 34 as above), including estimated LV mass (C), LVEF% (D), LVIDd (E), and LVIDs (F). (**G**) Representative H&E staining of LV samples in corresponding groups, indicating the degree of myocardial hypertrophy. (**H** and **I**) Representative Masson staining (H) and Sirius Red staining (I) of LV samples in three groups, showing the degree of fibrosis. (**J**) Representative WGA fluorescence indicating CM surface areas. Scale bars, 50 μm. (**K**) qPCR analysis of the relative mRNA expression of cardiac function indicators (*ANP* and *BNP*), hypertrophy marker (*b-MHC*), and fibrosis marker (*CTGF*) of LV tissues in three groups. *n* = 4 per group. (**L** and **M**) qPCR [(L), *n* = 4], representative Western blotting and quantification [(M), *n* = 3] of *ADAMTS2* in isolated CMs from the LV tissues of indicated mice, and *FN1* and *COL1A1* in isolated CFs from the LV tissues of three groups. (**N**) Graphic illustration of the main findings in this study. Kruskal-Wallis test and post hoc Dunn test were used in (A) to (F), (K), and (L). One-way ANOVA followed by the Bonferroni post hoc analysis were used in (M). *P < 0.05 and **P < 0.01. Each point represents the mean of an individual animal.

We measured the efficacy of probiotics at ameliorating cardiac remodeling at P7, P14, P21, and P28. Daily probiotics administration was associated with a more favorable gross evaluation (Fig. 7, A and B and figs. S16 and S23, A and B). Echocardiology at P28 demonstrated that probiotics administration was associated with decreased wall thickness, better cardiac function (LVEF%), and lower estimated mass of LV (Fig. 7, D to F). Probiotics administration significantly alleviated CM hypertrophy in H&E staining from P21 to P28 (Fig. 7G and figs. S23, C to F, and S24A). The degree of fibrosis was also mitigated by Masson and Sirius Red staining since P21 (Fig. 7, H and I, and figs. S23, C to F, and S24, B and C). WGA fluorescence represented relatively smaller CM areas in probiotics nAAC mice (Fig. 7J and fig. S24D). The mRNA expression of the heart failure markers *BNP* and *ANP*, the hypertrophy marker β*-MHC*, and the fibrosis marker *CTGF* were all reduced in the LV samples of the nAAC mice treated with probiotics (Fig. 7K). The mRNA expression level and the protein content of the AHR-targeted hypertrophy gene *ADAMTS2* were significantly reduced in CMs isolated from the LV of the probiotics nAAC mice (Fig. 7, L and M). *FN1* and *COL1A1* were down-regulated in CFs isolated from the LV of the nAAC mice treated with probiotics as well (Fig. 7, L and M). The foregoing results indicated that probiotics administration is potentially an efficacious treatment for poLV remodeling.

## DISCUSSION

In children with poLV, uncontrolled cardiac remodeling is associated with long-term exercise intolerance and poor quality of life (*4*–*6*). Therefore, it is important to recognize and prevent cardiac remodeling at early stages. In the present study, we identified the circulating plasma metabolite Kyn and established that it is clinically and mechanically linked to poLV remodeling in children. This gut microbiota metabolite promotes AHR nuclear translocation and activates hypertrophy/fibrosis genes in CMs and CFs. These processes are alleviated by reshaping the altered gut microbiota. The findings of the present study might provide insight into the mechanisms of poLV remodeling and could help improve the diagnosis and treatment efficacy of this condition. Moreover, the discoveries herein offer an accessible intervention and emphasized the importance of therapeutic modulation, rather than, exclusively, the surgical correction, of preventing cardiac remodeling and heart failure in children with LV obstruction.

In recent years, researchers strove to identify the functional metabolites in various diseases through unbiased metabolomics (*33*). Kyn is a major intermediate in tryptophan metabolism (*34*) and is found up-regulated in tumorigenesis (*35*). It was subsequently determined that Kyn is also vasoactive and proinflammatory (*36*) and thus recently became promising in the cardiovascular entities (*37*). However, its disease specificity still warrants investigations with different CVD models. The present study, using patient cohorts and corresponding mouse model, established Kyn as a potential biomarker of poLV remodeling in children. Here, Kyn correlated with multiple LV remodeling parameters. Kyn is superior as a biomarker (AUC = 0.720; *P* = 0.015), comparing to traditional heart failure indicators NT-BNP and cTNI, for early recognition of the remodeling in children with intact heart function or preclinical heart failure. Some other metabolites of Kyn and tryptophan metabolism may have different effects in cardiovascular landscape (*38*, *39*), yet none of these metabolites was found significant in our metabolomics. Hence, Kyn appears also a more important metabolite among the plasma tryptophan profile in poLV patients.

Intracellular Kyn is an endogenous AHR ligand. Nuclear AHR translocation was identified in embryogenesis, transformation, tumorigenesis, and inflammation (*24*). Previous studies on oncology (*19*, *21*, *22*) and the results of our animal model and human sample revealed no transcriptional change in total *AHR* content. By contrast, in poLV tissues exposed to excessive Kyn from circulation, nuclear translocated AHR initiates the transcription of the remodeling genes *ADAMTS2* (hypertrophy), *FN1*, and *COL1A1* (fibrosis). Hence, these conditions favored hypertrophic and fibrotic under pressure-induced, chronic remodeling. However, the effects of Kyn-AHR are diverse in different conditions. First reported in the late 1990s, AHR^−/−^ mice spontaneously developed mild cardiomyopathy and slight fibrosis as they aged (*40*). However, in the present study of young mice, neither fibrosis nor hypertrophy manifested in the AHR^−/−^ sham mice. A very recent literature has shown regenerative effects of the cardiac self-derived Kyn including angiogenesis and CMs proliferation in the newborn mice with apical heart resection (*41*). CM proliferation and angiogenesis are prominent processes in apical resection or ischemic events that help reduce fibrosis and preserve function. It is well consistent with our single-nucleus sequencing results of human poLV samples that high-plasma Kyn promoted angiogenesis and CM immaturity (as proliferation is one of the characteristics) in specific cell subclusters. However, in pressure-induced chronic remodeling, the above two processes might not be as prominent as that in direct injury. The protective effect of cardiac Kyn might be overwhelmed or blocked by hypertrophy and fibrosis induced by systemic circulating Kyn. Moreover, the impact of different cardiac diseases (acute or chronic, ischemic, or pre/afterload changes) on distant organs, especially gut microbiota, is divergent. This systemic influence might be more prominent in chronic poLV model. Therefore, we hypothesized that the effect of Kyn-AHR might have been age, ligand, and disease dependent. The use of a cell-specific knockout for *AHR* in CMs and CF might provide further information. These discoveries provided mechanistic evidence to the clinical findings why Kyn is related to poLV remodeling parameters and mechanistically supported Kyn to be potentially used for early recognition in the future.

Determining how Kyn is elevated in plasma stands, as the key question in developing a target therapy to block it, which is also one of the interesting findings of our research. As tryptophan is an essential amino acid obtained exclusively from diet, it was suspected that the gut microbiota was implicated in the poLV process. Several studies have elucidated the relationships between the metabolites produced by gut microbiota and cardiovascular health (*17*). Gut dysbiosis–associated trimethylamine/trimethylamine *N*-oxide (TMAO) and phenylacetylglutamine were closely associated with morbidity in cardiovascular events (*9*–*11*). Our young poLV mouse model also presented this heart-gut interaction. This study provided evidence for the relationships among poLV, gut microbiota, and Kyn. Metagenomics depicted the gut dysbiosis in nAAC mice. We also subjected GF mice with minimal bias from other variants to nAAC or sham feces. nAAC mice had significantly higher Kyn levels than the baseline and sham FMT mice, indicating nAAC gut microbiota as the potential source of excessive Kyn. Our findings were consistent with those of the previous work, where the altered gut microbiota tended to drive tryptophan in the direction of Kyn biosynthesis (*29*, *42*, *43*). It is worthwhile in the future study to investigate the Kyn synthesis pathways in the microbiota of nAAC mice and determine which specific microbes contribute to this process.

In our human and mouse metabolomics studies, none of the classic metabolites including TMAO was detected at a high level. Thus, there are wide differences between adult and children in terms of their gut microbiota metabolite profiles. Dietary differences between these two population groups might account for this discrepancy. The essential amino acid tryptophan and its metabolites are abundant in milk and vital in early life (*44*). Evidence had shown that the breast feeding shaped the composition of the gut microbiota in infants and caused it to convert tryptophan to its derivatives and activate AHR (*45*). It would be interesting to study what influence of a tryptophan-excessive diet/milk might have on heart failure and remodeling in children, which might provide evidence for the feeding advice to these pediatric patients.

In translational perspectives, several attempts were made to modulate tryptophan metabolism (*18*, *46*). However, synthetic tryptophan catabolism inhibitors (*47*) and Kyn-degrading enzymes (*48*) either have limited efficacy, induce side effects, or are cytotoxic. It is especially hard and restricted in its clinical application to children. Here, reshaping gut microbiota by oral probiotics provided therapeutic efficacy and minimally triggered intolerance or rejection in the mouse models. After treatment, the mice presented with healthier heart physiology and diminished expression of the remodeling genes downstream from Kyn-AHR. WGS analysis showed that probiotic supplementation introduced beneficial microorganisms into the gut microbiome and suppressed the growth of various pathogenic bacteria predominating in nAAC mice. In GF mice subjected to nAAC FMT, probiotic supplementation reduced excessive Kyn. In the joint analysis of metagenomics and metabolomics, probiotics treatment lowered the Kyn level possibly through *A. indistinctus*, *A. stercorihominis*, and *E. faecalis*. Therefore, specific microbes supplement is an accessible, convenient, safe, and effective method in treating poLV remodeling in our study, having promising translational value.

In conclusion, we observed that altered gut microbiota promoted poLV remodeling through excessive Kyn production. These discoveries may help clarify the pathogenesis of this disease in a systemic perspective. However, this study has some limitations. The patient sample size in validation cohort was relatively small, and future studies could use a larger cohort and a more age-matched or age-specific study. The dosage of Kyn and other modulators was relatively high in vitro studies. Investigation of the role of other cardiac cells expressing AHR would be also worthwhile in future studies. Cardiac- or cell-specific knockout would provide more information on Kyn-AHR axis. To accelerate clinical translation of probiotics, its safety and efficacy should be validated in future clinic trails. Together with the Kyn as a biomarker, our study may help predict poLV remodeling at early stage, when therapeutic intervention could be timely applied to prevent heart failure and improve prognosis.

## MATERIALS AND METHODS

### Study design

The overall objective of this study was to determine a plasma metabolite that is clinically linked to cardiac remodeling and heart failure in children. We focused on understanding the mechanism how this metabolite, Kyn, promotes remodeling and where it is derived and further developed a targeted treatment that blocks its action. We designed a pilot age-matched discovery cohort for untargeted metabolomics study (*n* = 10 versus 10; table S1) and a nonoverlapping validation cohort for quantification (*n* = 19 versus 25; table S3). The sample size was calculated with Power Analysis and Sample Size Software (PASS). For mechanism study, a corresponding nAAC young mice model and *AHR*^−/−^ mice were constructed in vivo, and using the CMs and CFs from both human and mice in vitro, we identified the Kyn-AHR–remodeling axis in poLV. Next, snRNA-seq was used to verify this mechanism in human heart. 16*S* and WGS metagenomics sequencing, GF mice, and fecal transplantation were used to identify that the excessive plasma Kyn was associated with altered gut microbiota in nAAC mice. On the basis of these findings, selected probiotics were then used as a potential treatment to reshape gut microbiota, reduce plasma Kyn, and relieve remodeling. Please refer to Supplementary Methods for further details on experimental procedures and detailed metabolomics data.

### Patients

Patient informed consent was obtained from all patients and their families. All human projects were conducted under the principle outlined in the Declaration of Helsinki and were approved by the Ethics Committee at Shanghai Children’s Medical Center. All experiments performed with patient samples were blinded. We designed a pilot age-matched discovery study (*n* = 20) and a validation study (*n* = 44) to evaluate the blood level of Kyn in pediatric patients with poLV diseases. The sample size was calculated with PASS. The enrollment criteria of poLV group were as follows: (i) patients’ age under 18 years old and (ii) preoperational diagnosed as poLV diseases by echocardiography and CT, including coarctation of aorta, aortic valve stenosis, LVOTO due to hypertrophic cardiomyopathy, or endocardial fibroelastosis. The pressure gradient of the stenosis site reached at least 45 mmHg in echocardiography; (iii) sign of LV hypertrophy in echocardiography, computed tomography (CT), or magnetic resonance imaging with either preserved or reduced LVEF%; and (iv) no evidence of other complex congenital heart disease, such as transposition of the great arteries, tetralogy of Fallot, or other genetic syndromes and extracardiac defects. The enrollment criteria of control group were as follows: (i) patients’ age under 18 years old; (ii) preoperational diagnosed as small (diameter less than 2 mm) atrial septum defect or ventricular septum defect measured by echocardiography and CT; (iii) no evidence of heart hypertrophy and remodeling with relatively intact LVEF%; and (iv) no evidence of other congenital heart disease, genetic syndromes, or extracardiac defects. The patients’ characteristics were shown in tables S1 and S3. Blood samples were obtained through central vein before operation or any other medical treatment. For single-nucleus RNA sequencing, LV tissues were obtained during operation in four patients with LVOTO, who needed surgical removal of the hypertrophied myocardium. The patients’ characteristics were shown in table S5.

### Mice

All animal experiments were conducted under National Institutes of Health guidelines (NIH publication no. 85-23, revised 1996), and the protocols were approved by the Animal Care and Use Committee at Shanghai Children’s Medical Center. The experimenters were blinded to each allocation in mice experiments at final evaluation. C57BL/6 mice (8 to 10 weeks old) were purchased from Shanghai Jihui Laboratory Animal Care Co. Ltd., and global *AHR* knock out (*AHR*^−/−^) mice lacking exon 2 on a C57BL/6 background (6 to 8 weeks old) were purchased from Shanghai Model Organisms Center Inc. The WT and *AHR*^−/−^ mice were then mated in breeding pairs to have *AHR*^+/−^ mice. The in vivo experiments used WT and *AHR*^−/−^ mice bred from *AHR*^+/−^ mice of the same litters. All nAAC and sham mice were cohoused with their mothers and breast fed until P27; the same standard sterilized water and irradiated fodder were provided after to diminish the diet variation and its impact on gut microbiota. Female and male mice were distributed in each group at random because gender features were not obvious in neonatal surgery; the sex of the P21 and P28 mice was determined at evaluation time points and was provided in table S7. All animals were kept in a 12-hour light and dark circle at room temperature. GF C57BL/6 mice (4 weeks old) were purchased from GemPharmatech Co. Ltd. and were housed in plastic flexible film gnotobiotic isolators with high efficiency particulate air filter (HEPA) filters and with access to sterilized water and food ad libitum. All experiments containing GF mice were conducted in GemPharmatech Gnotobiotic Facility.

### Statistical analysis

Each point in the patient study represents the mean from individual patient data or individual mice data. For in vivo and in vitro study, at least three biological repetitive experiments were performed.

SPSS 16.0 software (SPSS Inc.) was used for statistical analysis. The normality of the data was first assessed with Kolmogorov-Smirnov test. Differences between two normally distributed groups were examined using an unpaired, two-tailed Student’s *t* test. Differences among three more groups were analyzed with one-way analysis of variance (ANOVA) followed by the Bonferroni post hoc analysis. In the data not normally distributed, the unpaired two-tailed Mann-Whitney *U* test (two groups) or Kruskal-Wallis test (three or more groups), followed by Dunn post hoc test, was used. Linear regression was used to calculate the correlations. The statistical analysis in each multi-omics sequencing was provided in Supplementary Methods under each section. Continuous variables were presented as means ± SD. All *P* values were two sided, and *P* < 0.05 was considered statistically significant.

## Supplementary Materials

### This PDF file includes:

Supplementary Methods Figs. S1 to S24 Tables S1 to S9 Legend for movie S1 References

## Notes

View/request a protocol for this paper from *Bio-protocol*.

## References

[1] G. H. Kim, N. Uriel, D. Burkhoff, Reverse remodelling and myocardial recovery in heart failure. Nat. Rev. Cardiol. 15, 83–96 (2018). 28933783 10.1038/nrcardio.2017.139

[2] R. B. Hinton, S. M. Ware, Heart failure in pediatric patients with congenital heart disease. Circ. Res. 120, 978–994 (2017). 28302743 10.1161/CIRCRESAHA.116.308996 PMC5391045

[3] C. M. Otto, Aortic stenosis: Even mild disease is significant. Eur. Heart J. 25, 185–187 (2004). 14972416 10.1016/j.ehj.2003.12.010

[4] P. A. Pellikka, M. E. Sarano, R. A. Nishimura, J. F. Malouf, K. R. Bailey, C. G. Scott, M. E. Barnes, A. J. Tajik, Outcome of 622 adults with asymptomatic, hemodynamically significant aortic stenosis during prolonged follow-up. Circulation 111, 3290–3295 (2005). 15956131 10.1161/CIRCULATIONAHA.104.495903

[5] C. F. Azevedo, M. Nigri, M. L. Higuchi, P. M. Pomerantzeff, G. S. Spina, R. O. Sampaio, F. Tarasoutchi, M. Grinberg, C. E. Rochitte, Prognostic significance of myocardial fibrosis quantification by histopathology and magnetic resonance imaging in patients with severe aortic valve disease. J. Am. Coll. Cardiol. 56, 278–287 (2010). 20633819 10.1016/j.jacc.2009.12.074

[6] M. G. Del Buono, R. Arena, B. A. Borlaug, S. Carbone, J. M. Canada, D. L. Kirkman, R. Garten, P. Rodriguez-Miguelez, M. Guazzi, C. J. Lavie, A. Abbate, Exercise intolerance in patients with heart failure. J. Am. Coll. Cardiol. 73, 2209–2225 (2019). 31047010 10.1016/j.jacc.2019.01.072

[7] J. A. Hill, E. N. Olson, Cardiac plasticity. N. Engl. J. Med. 358, 1370–1380 (2008). 18367740 10.1056/NEJMra072139

[8] C. Andersson, C. Liu, S. Cheng, T. J. Wang, R. E. Gerszten, M. G. Larson, R. S. Vasan, Metabolomic signatures of cardiac remodelling and heart failure risk in the community. ESC Heart Fail. 7, 3707–3715 (2020). 32909388 10.1002/ehf2.12923 PMC7754777

[9] Z. Wang, E. Klipfell, B. J. Bennett, R. Koeth, B. S. Levison, B. Dugar, A. E. Feldstein, E. B. Britt, X. Fu, Y. M. Chung, Y. Wu, P. Schauer, J. D. Smith, H. Allayee, W. H. Tang, J. A. DiDonato, A. J. Lusis, S. L. Hazen, Gut flora metabolism of phosphatidylcholine promotes cardiovascular disease. Nature 472, 57–63 (2011). 21475195 10.1038/nature09922 PMC3086762

[10] W. H. Tang, Z. Wang, B. S. Levison, R. A. Koeth, E. B. Britt, X. Fu, Y. Wu, S. L. Hazen, Intestinal microbial metabolism of phosphatidylcholine and cardiovascular risk. N. Engl. J. Med. 368, 1575–1584 (2013). 23614584 10.1056/NEJMoa1109400 PMC3701945

[11] I. Nemet, P. P. Saha, N. Gupta, W. Zhu, K. A. Romano, S. M. Skye, T. Cajka, M. L. Mohan, L. Li, Y. Wu, M. Funabashi, A. E. Ramer-Tait, S. V. Naga Prasad, O. Fiehn, F. E. Rey, W. H. W. Tang, M. A. Fischbach, J. A. DiDonato, S. L. Hazen, A cardiovascular disease-linked gut microbial metabolite acts via adrenergic receptors. Cell 180, 862–877.e22 (2020). 32142679 10.1016/j.cell.2020.02.016 PMC7402401

[12] M. Schwenk, U. Gundert-Remy, G. Heinemeyer, K. Olejniczak, R. Stahlmann, W. Kaufmann, H. M. Bolt, H. Greim, E. von Keutz, H. P. Gelbke, Children as a sensitive subgroup and their role in regulatory toxicology: DGPT workshop report. Arch. Toxicol. 77, 2–6 (2003). 12491033 10.1007/s00204-002-0416-9

[13] A. Sandek, J. Bauditz, A. Swidsinski, S. Buhner, J. Weber-Eibel, S. von Haehling, W. Schroedl, T. Karhausen, W. Doehner, M. Rauchhaus, P. Poole-Wilson, H. D. Volk, H. Lochs, S. D. Anker, Altered intestinal function in patients with chronic heart failure. J. Am. Coll. Cardiol. 50, 1561–1569 (2007). 17936155 10.1016/j.jacc.2007.07.016

[14] A. Sandek, A. Swidsinski, W. Schroedl, A. Watson, M. Valentova, R. Herrmann, N. Scherbakov, L. Cramer, M. Rauchhaus, A. Grosse-Herrenthey, M. Krueger, S. von Haehling, W. Doehner, S. D. Anker, J. Bauditz, Intestinal blood flow in patients with chronic heart failure: A link with bacterial growth, gastrointestinal symptoms, and cachexia. J. Am. Coll. Cardiol. 64, 1092–1102 (2014). 25212642 10.1016/j.jacc.2014.06.1179

[15] N. Boccella, R. Paolillo, L. Coretti, S. D'Apice, A. Lama, G. Giugliano, G. G. Schiattarella, M. Cuomo, I. d'Aquino, G. Cavaliere, O. Paciello, M. P. Mollica, G. Mattace Raso, G. Esposito, F. Lembo, C. Perrino, Transverse aortic constriction induces gut barrier alterations, microbiota remodeling and systemic inflammation. Sci. Rep. 11, 7404 (2021). 33795775 10.1038/s41598-021-86651-y PMC8016915

[16] B. O. Schroeder, F. Bäckhed, Signals from the gut microbiota to distant organs in physiology and disease. Nat. Med. 22, 1079–1089 (2016). 27711063 10.1038/nm.4185

[17] W. H. Tang, T. Kitai, S. L. Hazen, Gut microbiota in cardiovascular health and disease. Circ. Res. 120, 1183–1196 (2017). 28360349 10.1161/CIRCRESAHA.117.309715 PMC5390330

[18] J. E. Cheong, L. Sun, Targeting the IDO1/TDO2-KYN-AhR pathway for cancer immunotherapy—Challenges and opportunities. Trends Pharmacol. Sci. 39, 307–325 (2018). 29254698 10.1016/j.tips.2017.11.007

[19] M. C. Lafita-Navarro, M. Kim, N. Borenstein-Auerbach, N. Venkateswaran, Y. H. Hao, R. Ray, T. Brabletz, P. P. Scaglioni, J. W. Shay, M. Conacci-Sorrell, The aryl hydrocarbon receptor regulates nucleolar activity and protein synthesis in MYC-expressing cells. Genes Dev. 32, 1303–1308 (2018). 30254109 10.1101/gad.313007.118 PMC6169836

[20] Y. Liu, X. Liang, W. Dong, Y. Fang, J. Lv, T. Zhang, R. Fiskesund, J. Xie, J. Liu, X. Yin, X. Jin, D. Chen, K. Tang, J. Ma, H. Zhang, J. Yu, J. Yan, H. Liang, S. Mo, F. Cheng, Y. Zhou, H. Zhang, J. Wang, J. Li, Y. Chen, B. Cui, Z. W. Hu, X. Cao, F. Xiao-Feng Qin, B. Huang, Tumor-repopulating cells induce PD-1 expression in CD8(+) T cells by transferring kynurenine and AhR activation. Cancer Cell 33, 480–494.e7 (2018). 29533786 10.1016/j.ccell.2018.02.005

[21] K. Kawajiri, Y. Kobayashi, F. Ohtake, T. Ikuta, Y. Matsushima, J. Mimura, S. Pettersson, R. S. Pollenz, T. Sakaki, T. Hirokawa, T. Akiyama, M. Kurosumi, L. Poellinger, S. Kato, Y. Fujii-Kuriyama, Aryl hydrocarbon receptor suppresses intestinal carcinogenesis in ApcMin/+ mice with natural ligands. Proc. Natl. Acad. Sci. U.S.A. 106, 13481–13486 (2009). 19651607 10.1073/pnas.0902132106 PMC2726415

[22] T. Ikuta, Y. Kobayashi, M. Kitazawa, K. Shiizaki, N. Itano, T. Noda, S. Pettersson, L. Poellinger, Y. Fujii-Kuriyama, S. Taniguchi, K. Kawajiri, ASC-associated inflammation promotes cecal tumorigenesis in aryl hydrocarbon receptor-deficient mice. Carcinogenesis 34, 1620–1627 (2013). 23455376 10.1093/carcin/bgt083

[23] J. Yang, K. Savvatis, J. S. Kang, P. Fan, H. Zhong, K. Schwartz, V. Barry, A. Mikels-Vigdal, S. Karpinski, D. Kornyeyev, J. Adamkewicz, X. Feng, Q. Zhou, C. Shang, P. Kumar, D. Phan, M. Kasner, B. López, J. Diez, K. C. Wright, R. L. Kovacs, P. S. Chen, T. Quertermous, V. Smith, L. Yao, C. Tschöpe, C. P. Chang, Targeting LOXL2 for cardiac interstitial fibrosis and heart failure treatment. Nat. Commun. 7, 13710 (2016). 27966531 10.1038/ncomms13710 PMC5171850

[24] C. A. Opitz, U. M. Litzenburger, F. Sahm, M. Ott, I. Tritschler, S. Trump, T. Schumacher, L. Jestaedt, D. Schrenk, M. Weller, M. Jugold, G. J. Guillemin, C. L. Miller, C. Lutz, B. Radlwimmer, I. Lehmann, A. von Deimling, W. Wick, M. Platten, An endogenous tumour-promoting ligand of the human aryl hydrocarbon receptor. Nature 478, 197–203 (2011). 21976023 10.1038/nature10491

[25] C. Koentges, M. E. Pepin, C. Müsse, K. Pfeil, S. V. V. Alvarez, N. Hoppe, M. M. Hoffmann, K. E. Odening, S. Sossalla, A. Zirlik, L. Hein, C. Bode, A. R. Wende, H. Bugger, Gene expression analysis to identify mechanisms underlying heart failure susceptibility in mice and humans. Basic Res. Cardiol. 113, 8 (2018). 29288409 10.1007/s00395-017-0666-6 PMC5764079

[26] X. Hua, Y. Y. Wang, P. Jia, Q. Xiong, Y. Hu, Y. Chang, S. Lai, Y. Xu, Z. Zhao, J. Song, Multi-level transcriptome sequencing identifies COL1A1 as a candidate marker in human heart failure progression. BMC Med. 18, 2 (2020). 31902369 10.1186/s12916-019-1469-4 PMC6943904

[27] C. D. Rau, M. C. Romay, M. Tuteryan, J. J. Wang, M. Santolini, S. Ren, A. Karma, J. N. Weiss, Y. Wang, A. J. Lusis, Systems genetics approach identifies gene pathways and Adamts2 as drivers of isoproterenol-induced cardiac hypertrophy and cardiomyopathy in mice. Cell Syst. 4, 121–128.e4 (2017). 27866946 10.1016/j.cels.2016.10.016 PMC5338604

[28] X. Wang, W. Chen, J. Zhang, A. Khan, L. Li, F. Huang, Z. Qiu, L. Wang, X. Chen, Critical role of ADAMTS2 (a disintegrin and metalloproteinase with thrombospondin motifs 2) in cardiac hypertrophy induced by pressure overload. Hypertension 69, 1060–1069 (2017). 28373586 10.1161/HYPERTENSIONAHA.116.08581

[29] T. Zelante, R. G. Iannitti, C. Cunha, A. De Luca, G. Giovannini, G. Pieraccini, R. Zecchi, C. D'Angelo, C. Massi-Benedetti, F. Fallarino, A. Carvalho, P. Puccetti, L. Romani, Tryptophan catabolites from microbiota engage aryl hydrocarbon receptor and balance mucosal reactivity via interleukin-22. Immunity 39, 372–385 (2013). 23973224 10.1016/j.immuni.2013.08.003

[30] M. C. Simon, K. Strassburger, B. Nowotny, H. Kolb, P. Nowotny, V. Burkart, F. Zivehe, J. H. Hwang, P. Stehle, G. Pacini, B. Hartmann, J. J. Holst, C. MacKenzie, L. B. Bindels, I. Martinez, J. Walter, B. Henrich, N. C. Schloot, M. Roden, Intake of Lactobacillus reuteri improves incretin and insulin secretion in glucose-tolerant humans: a proof of concept. Diabetes Care 38, 1827–1834 (2015). 26084343 10.2337/dc14-2690

[31] S. Khalesi, J. Sun, N. Buys, R. Jayasinghe, Effect of probiotics on blood pressure. Hypertension 64, 897–903 (2014). 25047574 10.1161/HYPERTENSIONAHA.114.03469

[32] R. M. Thushara, S. Gangadaran, Z. Solati, M. H. Moghadasian, Cardiovascular benefits of probiotics: A review of experimental and clinical studies. Food Funct. 7, 632–642 (2016). 26786971 10.1039/c5fo01190f

[33] M. M. Rinschen, J. Ivanisevic, M. Giera, G. Siuzdak, Identification of bioactive metabolites using activity metabolomics. Nat. Rev. Mol. Cell Biol. 20, 353–367 (2019). 30814649 10.1038/s41580-019-0108-4 PMC6613555

[34] P. Song, T. Ramprasath, H. Wang, M. H. Zou, Abnormal kynurenine pathway of tryptophan catabolism in cardiovascular diseases. Cell. Mol. Life Sci. 74, 2899–2916 (2017). 28314892 10.1007/s00018-017-2504-2 PMC5501999

[35] S. Adams, N. Braidy, A. Bessede, B. J. Brew, R. Grant, C. Teo, G. J. Guillemin, The kynurenine pathway in brain tumor pathogenesis. Cancer Res. 72, 5649–5657 (2012). 23144293 10.1158/0008-5472.CAN-12-0549

[36] Y. Wang, H. Liu, G. McKenzie, P. K. Witting, J. P. Stasch, M. Hahn, D. Changsirivathanathamrong, B. J. Wu, H. J. Ball, S. R. Thomas, V. Kapoor, D. S. Celermajer, A. L. Mellor, J. F. Keaney Jr., N. H. Hunt, R. Stocker, Kynurenine is an endothelium-derived relaxing factor produced during inflammation. Nat. Med. 16, 279–285 (2010). 20190767 10.1038/nm.2092 PMC3556275

[37] T. B. Dschietzig, K. H. Kellner, K. Sasse, F. Boschann, R. Klüsener, J. Ruppert, F. P. Armbruster, D. Bankovic, A. Meinitzer, V. Mitrovic, C. Melzer, Plasma kynurenine predicts severity and complications of heart failure and associates with established biochemical and clinical markers of disease. Kidney Blood Press. Res. 44, 765–776 (2019). 31387104 10.1159/000501483

[38] M. Ala, S. P. Eftekhar, The footprint of kynurenine pathway in cardiovascular diseases. Int. J. Tryptophan. Res. 15, 117864692210966 (2022). 10.1177/11786469221096643 PMC9248048 35784899

[39] T. Bekfani, M. Bekhite, S. Neugebauer, S. Derlien, A. Hamadanchi, J. Nisser, M. S. Hilse, D. Haase, T. Kretzschmar, M. F. Wu, M. Lichtenauer, M. Kiehntopf, S. von Haehling, P. Schlattmann, G. Lehmann, M. Franz, S. Möbius-Winkler, C. Schulze, Metabolomic profiling in patients with heart failure and exercise intolerance: Kynurenine as a potential biomarker. Cell 11, (2022). 10.3390/cells11101674 PMC9139290 35626711

[40] P. M. Fernandez-Salguero, J. M. Ward, J. P. Sundberg, F. J. Gonzalez, Lesions of aryl-hydrocarbon receptor-deficient mice. Vet. Pathol. 34, 605–614 (1997). 9396142 10.1177/030098589703400609

[41] D. Zhang, J. Ning, T. Ramprasath, C. Yu, X. Zheng, P. Song, Z. Xie, M. H. Zou, Kynurenine promotes neonatal heart regeneration by stimulating cardiomyocyte proliferation and cardiac angiogenesis. Nat. Commun. 13, 6371 (2022s). 36289221 10.1038/s41467-022-33734-7 PMC9606021

[42] L. Laurans, N. Venteclef, Y. Haddad, M. Chajadine, F. Alzaid, S. Metghalchi, B. Sovran, R. G. P. Denis, J. Dairou, M. Cardellini, J. M. Moreno-Navarrete, M. Straub, S. Jegou, C. McQuitty, T. Viel, B. Esposito, B. Tavitian, J. Callebert, S. H. Luquet, M. Federici, J. M. Fernandez-Real, R. Burcelin, J. M. Launay, A. Tedgui, Z. Mallat, H. Sokol, S. Taleb, Genetic deficiency of indoleamine 2,3-dioxygenase promotes gut microbiota-mediated metabolic health. Nat. Med. 24, 1113–1120 (2018). 29942089 10.1038/s41591-018-0060-4

[43] B. Lamas, M. L. Richard, V. Leducq, H. P. Pham, M. L. Michel, G. Da Costa, C. Bridonneau, S. Jegou, T. W. Hoffmann, J. M. Natividad, L. Brot, S. Taleb, A. Couturier-Maillard, I. Nion-Larmurier, F. Merabtene, P. Seksik, A. Bourrier, J. Cosnes, B. Ryffel, L. Beaugerie, J. M. Launay, P. Langella, R. J. Xavier, H. Sokol, CARD9 impacts colitis by altering gut microbiota metabolism of tryptophan into aryl hydrocarbon receptor ligands. Nat. Med. 22, 598–605 (2016). 27158904 10.1038/nm.4102 PMC5087285

[44] L. O'Rourke, G. Clarke, A. Nolan, C. Watkins, T. G. Dinan, C. Stanton, R. P. Ross, C. A. Ryan, Tryptophan metabolic profile in term and preterm breast milk: Implications for health. J. Nutr. Sci. 7, e13 (2018). 29686862 10.1017/jns.2017.69 PMC5906556

[45] M. F. Laursen, M. Sakanaka, N. von Burg, U. Mörbe, D. Andersen, J. M. Moll, C. T. Pekmez, A. Rivollier, K. F. Michaelsen, C. Mølgaard, M. V. Lind, L. O. Dragsted, T. Katayama, H. L. Frandsen, A. M. Vinggaard, M. I. Bahl, S. Brix, W. Agace, T. R. Licht, H. M. Roager, Bifidobacterium species associated with breastfeeding produce aromatic lactic acids in the infant gut. Nat. Microbiol. 6, 1367–1382 (2021). 34675385 10.1038/s41564-021-00970-4 PMC8556157

[46] M. Platten, E. A. A. Nollen, U. F. Röhrig, F. Fallarino, C. A. Opitz, Tryptophan metabolism as a common therapeutic target in cancer, neurodegeneration and beyond. Nat. Rev. Drug Discov. 18, 379–401 (2019). 30760888 10.1038/s41573-019-0016-5

[47] G. C. Prendergast, W. P. Malachowski, J. B. DuHadaway, A. J. Muller, Discovery of IDO1 inhibitors: From bench to bedside. Cancer Res. 77, 6795–6811 (2017). 29247038 10.1158/0008-5472.CAN-17-2285 PMC6021761

[48] T. A. Triplett, K. C. Garrison, N. Marshall, M. Donkor, J. Blazeck, C. Lamb, A. Qerqez, J. D. Dekker, Y. Tanno, W. C. Lu, C. S. Karamitros, K. Ford, B. Tan, X. M. Zhang, K. McGovern, S. Coma, Y. Kumada, M. S. Yamany, E. Sentandreu, G. Fromm, S. Tiziani, T. H. Schreiber, M. Manfredi, L. I. R. Ehrlich, E. Stone, G. Georgiou, Reversal of indoleamine 2,3-dioxygenase-mediated cancer immune suppression by systemic kynurenine depletion with a therapeutic enzyme. Nat. Biotechnol. 36, 758–764 (2018). 30010674 10.1038/nbt.4180 PMC6078800

[49] P. G. Rusconi, D. A. Ludwig, C. Ratnasamy, R. Mas, W. G. Harmon, S. D. Colan, S. E. Lipshultz, Serial measurements of serum NT-proBNP as markers of left ventricular systolic function and remodeling in children with heart failure. Am. Heart J. 160, 776–783 (2010). 20934575 10.1016/j.ahj.2010.07.012 PMC2964279

[50] R. B. Devereux, D. R. Alonso, E. M. Lutas, G. J. Gottlieb, E. Campo, I. Sachs, N. Reichek, Echocardiographic assessment of left ventricular hypertrophy: Comparison to necropsy findings. Am. J. Cardiol. 57, 450–458 (1986). 2936235 10.1016/0002-9149(86)90771-x

[51] Y. Gong, Z. Chen, L. Yang, X. Ai, B. Yan, H. Wang, L. Qiu, Y. Tan, N. Witman, W. Wang, Y. Zhao, W. Fu, Intrinsic color sensing system allows for real-time observable functional changes on human induced pluripotent stem cell-derived cardiomyocytes. ACS Nano 14, 8232–8246 (2020). 32609489 10.1021/acsnano.0c01745

[52] Y. Liu, Q. Luo, Z. Su, J. Xing, J. Wu, L. Xiang, Y. Huang, H. Pan, X. Wu, X. Zhang, J. Li, F. Yan, H. Zhang, Suppression of myocardial hypoxia-inducible factor-1α compromises metabolic adaptation and impairs cardiac function in patients with cyanotic congenital heart disease during puberty. Circulation 143, 2254–2272 (2021). 33663226 10.1161/CIRCULATIONAHA.120.051937

