# Supplementary material for: Targeting gut microbiota–derived kynurenine to predict and protect the remodeling of the pressure-overloaded young heart
Source: Sci Adv. 2023 Jul 14;9(28):eadg7417. doi: 10.1126/sciadv.adg7417 (PMC10348671; doi:10.1126/sciadv.adg7417)
Supplement: Supplementary file 1 — Supplementary Methods Figs. S1 to S24 Tables S1 to S9 Legend for movie S1 References [file sciadv.adg7417_sm.pdf]

Science Advances
AAAS

Supplementary Materials for

Targeting gut microbiota–derived kynurenine to predict and protect the remodeling of the pressure-overloaded young heart

Bozhong Shi *et al.*

Corresponding author: Xiaomin He, mrxmhe@163.com; Hao Zhang, drzhanghao@126.com; Jinghao Zheng, zhengjh210@163.com

Sci. Adv. **9**, eadg7417 (2023)
DOI: 10.1126/sciadv.adg7417

The PDF file includes:

Supplementary Methods
Figs. S1 to S24
Tables S1 to S9
Legend for movie S1
References

Other Supplementary Material for this manuscript includes the following:

Movie S1

Materials and Methods

Evaluation of ventricular remodeling in patients

Echocardiograms were performed in all patients to evaluate ventricular remodeling by a single experienced cardiologist following standard institutional protocols. M-mode tracing was obtained in the parasternal short-axis view at the level of the papillary muscles of the LV, and LV end-systolic and end-diastolic diameters were measured. Boyd weight-based formula was used for body surface area (BSA) and regression equations were used to calculate Z-scores(*49*). Relative wall thickness (RWT) and left ventricular mass index (LVMI) was calculated as pervious reported(*50*), where RWT=(IVSd+LVPWd)/LVIDd, LVMI=1.04×[(LVIDd+LVPWTd+IVSTd)^3-LVIDd^3]/BSA. Under colored tissue velocity imaging (TVI), the dynamic images of apical two-chamber section were collected and stored in EchoPAC-PC (version 2.0) ultrasound workstation and analyzed using the Quantitative Analysis function of QTVI software. Peak systolic stress (PSS, g/cm^2) at the mid wall was obtained by analyzing the stress curves of two sampling points, which were placed in the anterior basal and middle subendometrial myocardium. Because CT and MRI were not be able to conduct in all patients, these data were not compared. Traditional blood indices, including classic cardiac function parameter NT-proBNP, and myocardium injury parameter cTNI, were tested in all patients before any treatment.

Untargeted metabolomics analysis

Human patients and mice were fasted from the night before blood collection. Blood samples were obtained and were centrifuged to collect plasma within an hour. Blood samples of 4-week age nAAC (n=10) or sham mice (n=10) were taken through orbital vein and centrifuged. The following untargeted metabolomics procedures were conducted by Biotree biotech Co., Ltd. (Shanghai, China). For metabolites extraction, 100 μl of sample was firstly added with 400 μl of extract solution (acetonitrile: methanol = 1: 1, containing isotopically-labelled internal standard mixture), the samples were sonicated in ice for 10 min and incubated for 1h at -40 ℃ to precipitate proteins, followed by a centrifuge at 12000 rpm for 15 min at 4 ℃. The supernatant was transferred to a fresh glass vial for analysis. An equal aliquot of the supernatants from all samples were mixed as the quality control (QC) sample. LC-MS/MS analyses were performed using an UHPLC system (Vanquish, Thermo Fisher Scientific) with a UPLC BEH Amide column (2.1 mm × 100 mm, 1.7 μm) coupled to Q Exactive HFX mass spectrometer (Orbitrap MS, Thermo). The mobile phase contained 25 mmol/L ammonium acetate and 25 ammonia hydroxide in water (pH = 9.75) (A) and acetonitrile (B). The auto-sampler temperature was set to 4 ℃, and the injection volume was 3 μl. The QE HFX mass spectrometer was employed to acquire MS/MS spectra on information-dependent acquisition (IDA) mode in the control of the acquisition software (Xcalibur, Thermo), which continuously evaluated the full scan MS spectrum. The raw data were converted to the mzXML format by ProteoWizard and processed with an in-house program based on R package XCMS (version 3.2)[48], for peak detection, extraction, alignment, and integration. Then an in-house MS2 database (BiotreeDB)

was applied in metabolite annotation. The cutoff for annotation was set at 0.3. Statistical significance was calculated between groups using multiple t-test. Adjusted P values were generated from P values corrected by false discovery rate (FDR) based on Benjamini-Hochberg procedure. Significantly different metabolites were selected through a criteria of: (1) variable importance in projection (VIP) >1; (2)Adjusted P value <0.05.

UHPLC-MS/MS analysis

To quantify Kyn level in plasma of patients and mice, UHPLC-MS/MS analysis was used and was conducted by Biotree biotech Co., Ltd. (Shanghai, China). 100 μl of each sample was added with 400 μl extract solvent (acetonitrile-methanol, 1:1, containing internal standard and 0.1% formic acid, precooled at -40 ℃). The samples were sonicated for 15 min in ice-water bath and incubated at -40 ℃ for one hour, then underwent a centrifugation at 12000 rpm and 4 ℃ for 15 min. A 400 μl aliquot of supernatant was dried by spin, and was reconstituted with 100 μl water with 0.1% formic acid. After vortexing for 15s, the samples were centrifuged again at 12000 rpm and 4 ℃ for 15 min. 90 μl aliquot of the clear supernatant was transferred to an auto-sampler vial for UHPLC-MS/MS analysis. Mixed standard solution was prepared by stepwise dilution of 10 mmol/L stock solution. The UHPLC separation was carried out using an EXIONLC System (SCIEX AB), equipped with a Waters ACQUITY UPLC HSS T3 (100 × 2.1 mm, 1.8 μm, Waters). The mobile phase A was 0.1% formic acid in water, and the mobile phase B was 0.1% formic acid in acetonitrile. The column

temperature was set at 40 °C. The auto-sampler temperature was set at 4 °C and the injection volume was 10 µl. A SCIEX 6500 QTRAP + triple quadrupole mass spectrometer (Sciex), equipped with an IonDrive Turbo V electrospray ionization (ESI) interface, was applied for assay development. The MRM parameters for each targeted analytes were optimized by injecting the standard solutions of the individual analytes into the API source of the mass spectrometer. Several most sensitive transitions were used in the MRM scan mode to optimize the collision energy for each Q1/Q3 pair. Among the optimized MRM transitions per analyte, the Q1/Q3 pairs that showed the highest sensitivity and selectivity were selected as 'quantifier' for quantitative monitoring. The additional transitions acted as 'qualifier' for the purpose of verifying the identity of the target analytes. SCIEX Analyst Work Station Software (Version 1.6.3) and Sciex MultiQuant™ 3.0.3 were employed for MRM data acquisition and processing. The final concentration (cF, nmol/L) equals the calculated concentration (cC, nmol/L) multiplied by the dilution factor (Dil). The metabolite concentration (CM, nmol/L) equals the final concentration (CF, nmol/L) multiplied by the sample experiment concentration factor (CF) and the final volume (VF, µL), and divided by the volume (VS, µL) of the sample.

Neonatal ascending aorta constriction (nAAC) mice model

Ascending aorta constriction surgery was performed in mice pup (WT or *AHR*$^{-/-}$) within 24 h after birth (Video 1, Data Supplement). The mice pup was firstly placed in an ice bath for 5 minutes to induce hypothermia and cardiac asystole. Then the pup was

removed to an ice pack covered with gauze and was fixed with tapes. Under the microscope, a median 5mm transverse skin incision and sternum transection were made between 1th-2nd ribs, followed by gently pulling aside the thymus and isolating ascending aorta with two blunt forceps. Gelatin sponge could be employed to control minor hemorrhage when necessary. Inserting a 10-0 suture under the ascending aorta with the aid of a curved 32G blunt needle. Then a 3 mm 28G needle was placed on and ligated with the ascending aorta, which was not performed in sham surgery. The needle was then gently removed in nAAC surgery to induce corresponding constriction. Closing the thorax by an 8-0 suture around the adjacent ribs, and closing the skin with three interrupted 8-0 suture. The mice pup was removed to a heat pad (37 °C) to restore its body temperature, allowing for recovery. Due to the absence of centralized pain reflexes at early postnatal age, no pain relief medications are required for P1 neonatal mice after surgery. The whole surgery could generally be performed within 10-15 minutes. All surgery in our experiments was conducted by the same surgeon with over one-year nAAC experience. Female and male mice were distributed in each group at random since gender features were not obvious in very early neonatal stage. The gender feature was determined at evaluation time points in mice after P21. The mice were harvest at indicated time points for different experiments.

Transcriptome sequencing

LV tissues were separated from 4 week-age nAAC (n=5) or mice (n=5) without septum. RNA extraction from mice LV tissues was performed by regular Trizol method. 1 μg

RNA per sample was used as input material for the RNA sample preparations. Sequencing libraries were generated using NEBNext® UltraTM RNA Library Prep Kit for Illumina® (NEB, USA) following manufacturer’s recommendations and index codes were added to attribute sequences to each sample.

The library fragments were purified with AMPure XP system (Beckman Coulter, Beverly, USA) to select cDNA fragments of preferentially 250~300 bp in length. The clustering of the index-coded samples was performed on a cBot Cluster Generation System using TruSeq PE Cluster Kit v3-cBot-HS (Illumia) according to the manufacturer’s instructions. After cluster generation, the library preparations were sequenced on an Illumina Novaseq platform and 150 bp paired-end reads were generated. The downstream analysis was based on clean, high quality data filtered through in-house Peri scripts. Differential expression analysis was peformed using the DESeq2 R package (1.16.1). Gene with adjusted P value <0.05 and fold change >0.5 were used for following IPA analysis. Ingenuity® Pathway Analysis software was used for IPA analysis to filter the genes that putatively regulated by AHR.

Single-nucleus RNA-sequencing

Single-nucleus RNA-seq experiment of 4 human LV tissues were performed in the laboratory of NovelBio Co., Ltd (Shanghai, China). The nucleus was isolated and purified as previously described with some modifications. Briefly, the liquid nitrogen frozen LV tissues were homogenized in NLB buffer which contain 250 mM Sucrose, 10 mM Tris-HCl, 3 mM MgAc2, 0.1% Triton X-100 (SigmaAldrich, USA), 0.1 mM

EDTA, 0.2U/μL RNase Inhibitor (Takara, Japan). The concentration of nucleus was adjusted to approximately 1000 nuclei/μl for snRNA-Seq. The snRNA-Seq libraries were generated using the 10X Genomics Chromium Controller Instrument and Chromium Single Cell 3' V3.1 Reagent Kits (10X Genomics, Pleasanton, USA). Briefly, cells nuclei were loaded into each channel to generate single-cell Gel Bead-In-Emulsions (GEMs). After the RT step, GEMs were broken and barcoded-cDNA was purified and amplified. The amplified barcoded cDNA was fragmented, A-tailed, ligated with adaptors and index PCR amplified. The final libraries were quantified using the Qubit High Sensitivity DNA assay (Thermo Fisher Scientific, USA) and the size distribution of the libraries were determined using a High Sensitivity DNA chip on a Bioanalyzer 2200 (Agilent, USA). All libraries were sequenced by Novaseq6000 (Illumina, USA) on a 150 bp paired-end run.

snRNA-seq data analysis was performed by NovelBio Bio-Pharm Technology Co.,Ltd. with NovelBrain Cloud Analysis Platform. We applied fastp with default parameter to filter the adaptor sequence and removed the low quality reads. Then the feature-barcode matrices were obtained by aligning reads to the human genome (GRCh38_Ensembl_Ensembl100) using CellRanger v5.0.1. We applied the down sample analysis among sequenced samples according to the mapped barcoded reads per cell of each sample and finally achieved the aggregated matrix. Cells contained over 200 expressed genes and mitochondria UMI rate below 10% passed the cell quality filtering and mitochondria genes were removed in the expression table.

Seurat package (version: 3.1.4, https://satijalab.org/seurat/) was used for cell normalization and regression based on the expression table to obtain the scaled data. The fastMNN function (k = 5, d = 50, approximate = TRUE) in the R package scran (v1.12.1) was used to apply the mutual nearest-neighbour method to correct for batch effect among samples. PCA was constructed according to the scaled data with high variable genes (top 2000), and top 10 principals were used for tSNE construction and UMAP construction. Utilizing graph-based cluster method, the unsupervised cell cluster result was acquired based on the PCA top 10 principal. The marker genes were calculated by FindAllMarkers function with wilcox rank sum test algorithm under following criteria:1) lnFC > 0.25; 2) p value<0.05; 3) min.pct>0.1. In order to identify the cell type in detail, the clusters of same cell type were selected for re-tSNE analysis, graph-based clustering and marker analysis.

16S ribosomal RNA sequencing

Total bacterial DNA were extracted from samples using the Power Soil DNA Isolation Kit(MO BIO Laboratories) according to the manufacturer's protocol. The V3-V4 region of the bacterial 16S rRNA gene was amplified with the common primer pair (Forward primer, 5'- ACTCCTACGGGAGGCAGCA-3'; reverse primer, 5'-GGACTACHVGGGTWTCTAAT-3') combined with adapter sequences and barcode sequences. High-throughput sequencing analysis of bacterial rRNA genes was performed on the purified, pooled sample using the Illumina NovaSeq 6000 platform (2×250 paired ends). The raw data was filtered using Trimmomatic (version 0.33), then

the primer sequences were identified and removed using Cutadapt (version 1.9. 1). USEARCH (version 10) was used to splice and remove the two-ended reads (UCHIME [4], version 8.1) and the high sequence was used for subsequent analysis. DADA2 method in QIIME2 (versoin 2020.6) was used for denoising the data after quality control, 0.005% of all sequences is used as the threshold to filter ASVs. Twelve samples were sequenced for a total of 959,881 pairs of reads. After quality control splicing, 957,739 clean reads were produced. Each sample produced at least 79,650 clean reads and an average of 79,812 clean reads. Refraction was used to evaluate whether the sequencing quantity is sufficient to cover all groups. Sequences with ≥97% similarity were assigned to the same operational taxonomic units (OTUs). Based on the normalized OTUs, alpha diversity was applied to analyse the complexity of species diversity for the samples. ANOISM analysis was used to evaluate differences among the samples in species complexity. The reference taxonomy was

Silva (Release138, http://www.arb-silva.de), Unite (Release 8.0, https://unite.ut.ee/), Greengenes (version 13.5, http://greengenes.secondgenome.com/), NCBI(ftp://ftp.ncbi.nlm.nih.gov/refseq/TargetedLoci/), fungene (http://fungene.cme.msu.edu/), MaarjAM (https://maarjam.ut.ee/).

Shotgun metagenomics analysis

Colon content was collected from 4-wks mice in a sterile tube and snap frozen. Whole genome shotgun sequencing (WGS) was performed by the Biotree biotech Co., Ltd. (Shanghai, China). Stool sample DNA was extracted using the SDS method. All

samples were sequenced on an Illumina platform in PE150 mode. Adapter was trimmed and low-quality reads were filtered using trimmomatic-0.39. Then, host sequences were removed by aligning sequencing reads back to host genome reference (GRCm39/mm39) using bowtie2 (version2.4.1). Functional profiling was performed by HUMAnN3. Sample reads are mapped against this database to quantify gene presence and abundance on a per-species basis. A translated search is then performed against a UniRef-based protein sequence catalogue for all reads that fail to map at the nucleotide level. The result are abundance profiles of gene families (UniRef90s), stratified by each species contributing those genes, and which can then be summarized to higher-level gene groupings such as ECs or KOs. Alpha diversity was measured by observed counts and Shannon index with an in-house Perl script. Bray-Curtis distance was calculated using python module scipy (1.5.1). Principal coordinates analysis (PCoA) was performed using the Bray-Curtis distance matrix data in R with ggplot2. LEfSe (Linear discriminant analysis Effect Size) was performed with LEfSe (version 1.0) software. Wilcoxon rank-sum test (two-tailed) was used for testing the difference between two groups and Kruskal-Wallis test was used for testing the difference between three groups. Multiple testing was adjusted using the Benjamin-Hochberg method. Permutation multivariate analysis of variance (PERMANOVA) test was used for the difference of β diversity between the compared groups.

Probiotics administration in nAAC mice

For oral probiotics administration, each probiotics capsule containing more than 1.0×10^7 colony-forming unit (CFU) viable *Bifidobacterium_longum* 913*, Lactobacillus_acidophilus* 145 and *Enterococcus_faecalis* ATCC19433 (BeNa Culture Collection, China)*,* was suspended into 1 ml sterile saline. After nAAC surgery, mice were gavaged daily with 0.015ml/g probiotics slurry until euthanize, or saline as control. To be noted, gavage in mice under 2-week age was operated with great care. Instead of using the regular steel gavage needle, 26G soft venous trocar tube was curved at tip and inserted gently beyond pharyngeal, and the gavage was controlled in a smooth and slow rate. The success of gavage was confirmed by the inflate of stomach that could be observable. Mice that died within 10 minutes after gavage procedure were attributed to gavage injury and were excluded. All mice were co-housed with their mothers and breast fed before P28, then were provided with standard fodder, in order to diminish the diet variation and its impact on gut microbiota.

Germ-free mice treatment

Germ-free (GF) mice (4-week age, 5 male and 5 female per group) were used to study the relationship of gut microbiota and blood Kyn (Supplementary Figure 11). Baseline blood samples were firstly collected through orbital vein before treatment. Then fecal microbiota transplantation (FMT) was conducted for 2 weeks in GF mice, where fecal pellets were collected from donor nAAC or sham C57BL/6 mice (4-week age) in an empty, sterile plastic cage. The gender of GF mice and nAAC/sham donor mice was also matched (5 male vs 5 female per group). The fecal supernatant processing was

conducted in germ-free working hood. 50 mg collected feces were suspended in 1 ml saline. The suspension then underwent centrifuge (100×g for 2 min) to pellet large particles and collect supernatant for subsequent FMT (0.015ml/g daily). The final products were used immediately for FMT via oral gavage. After 2 weeks of FMT, a second blood collection was performed. Then the mice with nAAC FMT underwent probiotics gavage for another 2 weeks and then the third blood collection was performed. All mice were fed with sterilized water and food ad libitum.

Kyn injection in mice

L-Kynurenine (K8625, CAS:2922-83-0, Sigma) was injected intra-peritoneally every day after surgery at the dose of 50 mg/kg to induce detectable phenotypes. Same amount of saline was injected in control group.

Isolation and culture of primary neonatal mouse cardiomyocytes and cardiac fibroblasts

Primary neonatal mouse cardiomyocytes (mCM) and cardiac fibroblasts (mCF) were obtained from neonatal C57BL/6 using Neomyt kit (no.nc-6031, Cellutron). Briefly, for each time experiment, LV tissues were harvest from 10 neonatal C57BL/6 mice within 24h after birth and firstly digested for 12 min in 10 ml enzyme buffer. The supernatant was collected in a new tube. 4 ml of new enzyme buffer was added to the remaining tissues and digested for 15min. This digestion steps were repeated for 7-9 times until fully digested. The supernatant collection from each round underwent 1200

rpm, 1 min centrifuge to obtain pellets of digested cells. The pellet was resuspended in DMEM/ F12 culture medium (no.11320033, Gibco) and plated onto 6 cm culture plates. mCF was obtained 2 hours after initial plating. The unattached cells in culture medium were transferred onto new plates to obtain mCM. Cells were cultured for 24h and then were ready for later treatment.

Generation and culture of hiPSC-CMs

HiPSCs, kindly offered by Professor Bei Feng (Shanghai Children's Medical Center, Shanghai), were cultured and differentiated into cardiomyocytes as previously reported(*51*). Breifly, the hiPSCs were dissociated and re-plated onto Matrigel (no.354234, Corning) coated 12-well plates before commencing differentiation at 100,000-200,000 cell/cm^2. Generally, Giwi protocol regulating Wnt signaling pathway was applied to induce hiPSC to hiPSC-CM. On day 0, 12 μM selective GSK3 inhibitor CHIR99021 (no.72054, Stemcell) was added into RPMI 1640/B27 medium without insulin (RPMI1640/B27-ins) (no.11875093/A1895601, Gibco) for 24h. On day 3, 5 μM Wnt inhibitor IWP2 (no.72124, Stemcell) was added into RPMI1640/B27-ins medium to lead hiPSC into cardiac lineage. The medium was changed till day 5. On day 7, the medium was replaced by RPMI1640/B27 with insulin (RPMI1640/B27+ins) (no.11875093/17504044, Gibco) and changed every other day until Day 15, when the well achieved hiPSC-CMs were identified and ready to be used for later treatment.

Cell treatment

For in vitro study, primary neonatal mouse CM and mouse CF, hiPSC-CM, adult human cardiac fibtoblasts (hCF) (306-5A, Sigma) were employed. Cells were seeded onto 12-well plates and were cultured for 24h to reach stable status before treatment. 500 μM Kyn (K8625, CAS:2922-83-0, Sigma), 500 μM L-amino acid transporter inhibitor BCH (A7902, CAS:20448-79-7, Sigma), 500 μM AHR inhibitor CH223191 (C8124, CAS:301326-22-7, Sigma), 500 μM AHR activator TCDD (CAS:1746-01-6, Sigma) were added into culture medium as indicated combination. Same amount of DMSO was added as control. The sequential analysis was carried out after 48h of treatment.

Isolation of cardiomyocytes and cardiac fibroblasts from 4w mice

Isolation and harvest of cardiomyocytes and cardiac fibroblasts from young mice were conducted according to a modified protocol as reported previously(*52*). 4-week age WT or *AHR*$^{-/-}$ mice after sham or nAAC surgery, with or without Kyn injection or probiotics administration, were used for CM and CF isolation. Briefly, the mice was firstly anesthetized and fixed, the chest was then opened in a U-shaped incision. The descending aorta and inferior vena cava were transected at diaphragm level, and 5 ml EDTA buffer was quickly injected into the right ventricle with 30G needle. Then the ascending aorta was ligated with 6-0 thread above the origin of coronary artery. 5 ml EDTA buffer was injected at the apex of left ventricle, followed by 3 ml perfusion buffer and 40 ml collagenase buffer. The heart was cut off and gently pulled and pipetted into cellular debris in a 6 mm plate with 5 ml stop buffer. The cell suspension was passed through 100 μm filter and divided into two 15 ml tube. Then the suspension

was allowed for gravity settling for 20 min and another three rounds of 10 min gravity settling in 4 ml perfusion buffer. CM were obtained at the bottom of the tube after final round of gravity settling. Supernatant of each round were collected together and underwent 1000 rpm, 5 min centrifuge to obtain CF pellet. The isolated CM and CF were used for further qPCR and Western blot analysis.

Chromatin Immunoprecipitation followed by qPCR (ChIP-qPCR)

For ChIP-qPCR analysis, hCFs and hiPSC-CMs were seeded onto a 10-cm dish respectively and pre-treated with Kyn (500 μM) for 48 h. ChIP-qPCR was performed using SimpleChIP Enzymatic Chromatin IP Kit (no. 9002S, Cell Signaling Technology) according to the manufacturer's instructions. Briefly, approximately eight million cells were immunoprecipitated with 5 μg anti-AHR antibody (no.83200, Cell Signaling Technology). Anti-rabbit IgG (no.2729, Cell Signaling Technology) was used as control. The percentage input method was used to normalize chromatin immunoprecipitation data.

DRE luciferase reporter gene activity

DRE-driven reporter gene activity in hCFs and hiPSC-CMs were conducted similar to previous reported[24]. Briefly, pGL4.27-hDRE plasmid and the control plasmid pRL-SV40 (no. E2231, Promega), expressing renilla luciferase were used. hCFs and hiPSC-CMs were cultured in 24-well plates and transiently transfected with 1.2 μg pGL4.27-hDRE reporter gene construct, 10 ng pRL-SV40 control plasmid, using Lipofactamine

2000 kit (no.11668500, Thermo Fisher Scientific) according to the manufacturer's instructions. Cells were then incubated with 500µM Kyn or DMSO for 48 h. Then cells were harvested by cell reporter gene lysates (no.RG126, Beyotime). Firefly luciferase reporter gene assay kit (no.RG006, Beyotime) and renilla luciferase reporter gene assay kit (no.RG062, Beyotime) were used according to the manufacturer's instructions. The luciferase activity was measured by luminescence detector (30IOC, Promega).

In vitro immunofluorescence of AHR translocation

HiPSC-CMs and hCFs were seeded in 12-well glass bottom plates (no.P24-1.5H-N, Cellvis) at the density of 10,000 cells/cm^2. Cells were exposed to 500 µM Kyn, or DMSO as control. After 48h treatment, cells were fixed with pre-warmed 4% formaldehyde for 30 min. Then 0.1% Triton X-100 was used for 30 min permeabilisation, and washed by PBS three times. At room temperature, cells were incubated with 1:100 diluted AHR antibody conjugated with Alexa® Fluor 647 (no.ab225358, Abcam) for 30 min, then incubated with 1:100 diluted phalloidin conjugated with iFluor 488 (no.ab176753, Abcam) for 30 min to shown cell skeleton. Finally, 1 µg/ml DAPI (no.D9542, Sigma) was added for 5 min and washed by PBS three times. The plates were sealed and protected from light. The imaging was acquired by a laser confocal microscope system (TCC SP8, Leica, Germany).

Scratch assay

mCFs and hCFs were evenly seeded in 12-well plates and were allowed for growth to reach a density of 1000,000 cells/cm^2. A linear scratch was performed in the middle of each well by the tip of a 100 μl pipette. Different reagents with indicated dosage were added into the culture medium. Cells were allowed to migrate into the defined gap. After indicated time, microscope photographs were taken.

CCK-8 assay

mCF and hCF cells were evenly seeded in 96-well plates and growth to reach a density of 1000,000 cells/cm^2. Cells were cultured with indicated reagents for 48h. Wells without treatment were used as negative control and wells without cells were used as blank control. 10 μl CCK-8 solution (A311, Vazyme) was added to each well and incubated in 37 °C for 4h. Absorbance was measured by a microplate reader (Multiskan MK3, Thermo Electron Corporation, USA) at 450 nm.

Cell surface area measurement

Unstained mCM and hiPSC-CM were pictured 48h after indicated treatment under bright field, and was the cell surface area was measured by ImageJ (National Institute of Health, Bethesda, Maryland, USA; Laboratory for Optical and Computational Instrumentation, University of Wisconsin, Madison, Wisconsin, USA). Approximately 4,000-5,000 cardiomyocytes in different fields were examined under each treatment.

Protein/DNA ratio analysis

For protein/DNA ratio analysis to evaluate cardiomyocytes hypertrophy, after 48h indicated treatment, mCM and hiPSC-CM were harvested, evenly resuspended, and were equally separated into two EP tubes. Total protein in one tube was extracted by RIPA buffer with protease inhibitor (Roche) and quantified by BCA method (no.E112, Vazyme) according to the manufacture's instruction. Another half of the cells were extracted for total DNA using DNA extraction kit (no. 4413021, Thermo Fisher Scientific) and quantified by Nanodrop (Thermo Fisher Scientific).

Histological analysis

Histological analysis of the LV tissues was carried out as published. Briefly, hearts were firstly excised and the vessels, atriums and the right ventricle were separated form LV. The LV tissues were fixed in 10% formalin, embedded in paraffin and sectioned into 7 μm slices, and stained with hematoxylin-eosin (HE) (no.G1120, Solarbio), or standard Masson trichrome staining (no.G1340, Solarbio), or Sirius red staining (no.S8060, Solarbio) according to manufacturer's instructions. To measure the cross-sectional area of the cardiomyocytes, the sections were stained with conjugated wheat germ agglutinin (no.178444, Abcam) according to the manufacturer's instructions.

qRT-PCR analysis

Total RNA was extracted from cells or LV tissues by Trizol method (no.15596018, Thermo Fisher Scientific), as previously reported(*52*). Reverse transcription was performed by HiScript III RT Super Mix Kit (no. R323, Vazyme) according to

manufactures instruction. The final qRT-PCR was performed by life Technology ABI 7500 System using ChamQ Universal SYBR qPCR Master Mix (no. Q711, Vazyme). The relative mRNA expression was calculated based on delta-delta CT method as previously reported(*52*).

Protein extraction and Western blot analysis

Total protein was extracted by RIPA buffer with protease inhibitor (Roche). The protein content from nuclear and cytoplasmic fraction was isolated using a Nuclear and Cytoplasmic Protein Extraction Kit (abs9346, Absin). Extraction was performed according to the manufacture's instruction. Briefly, 200 µl of cytoplasmic protein extraction buffer was added into 2000,000 cells or 100mg tissues, and was vortexed for 20 min in 4 °C. The slurry was pipetted for 90 times and underwent 15000g centrifuge for 2 min. The supernatant was collected as cytoplasmic protein. Another 400 µl of cytoplasmic protein extraction buffer was added into the pellet and underwent 15000g centrifuge for 10 min, then the supernatant was discarded. 100 µl nuclear protein extraction buffer was added and was vortexed for 5 min in 4 °C. followed by 15000g centrifuge for 10 min. The supernatant was collected as nuclear protein.

For western blot experiment, protein samples were resolved in SDS-PAGE and transferred onto nitrocellulose membranes using an iBlot 2 dry bloting system (Thermo Fisher Scientific). 5% non-fat dry milk in TBST (100 mmol/L Tris, pH7.5, 0.9%NaCl, 0.1%Tween-20) was used as blocking buffer for 2h. Then the membrane was incubated

with primary antibodies at 4 ℃ for 12h, followed by corresponding horseradish-peroxidase-labled secondary antibody at room temperature for 1h after 3 times of TBST washing. For visualization of the protein band, enhanced chemiluminescent substrate (no. WBKLS500, Millipore) was used. AlphaView Software (ProteinSimple) was used for analysis of the gray intensity.

Supplemental Figures

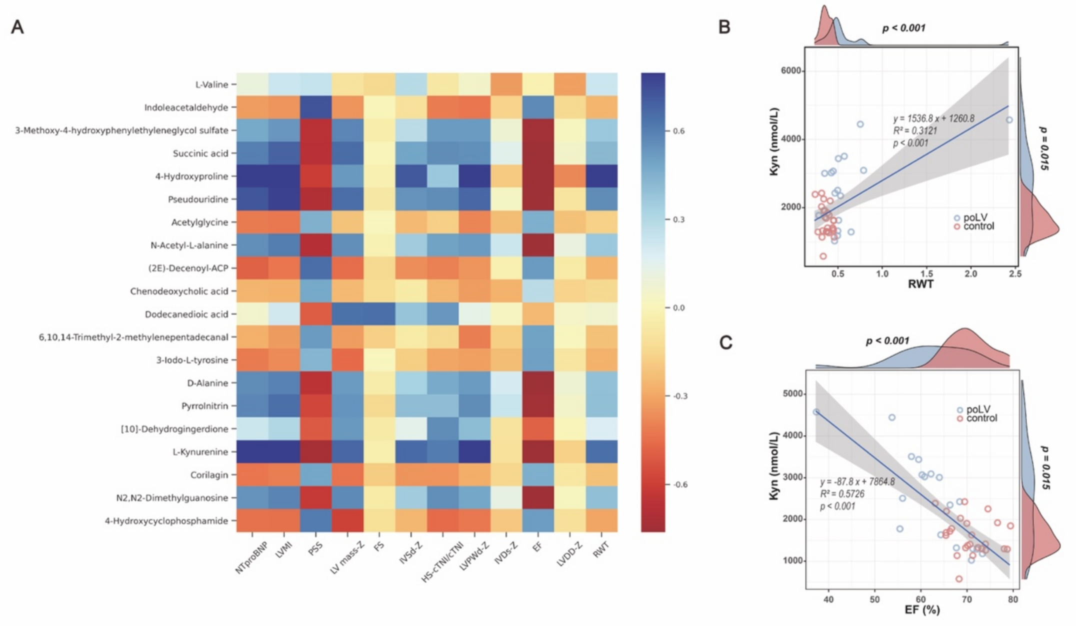

Fig. S1. Kyn is associated with impaired cardiac functions and remodeling degree in poLV children.

(**A**) Primary correlation analysis between plasma metabolites in untargeted metabolomics and patients' clinical data. Notable correlation was shown between Kyn and LVPWd-Z score, LVMI, RWT, EF, FS, PSS. (**B**) Linear regression showed correlation between Kyn and RWT. (**C**) Linear regression showed correlation between Kyn and EF. EF=ejection fracture; FS= fraction shortening; LVIDd=left ventricular internal diameter at end-diastole; LVIDs=left ventricular internal diameter at end-systole; IVSd= internal diameter of septum at end-diastole; LVPWd= left ventricular posterior wall thickness at end-diastole; LVPWs= left ventricular posterior wall thickness at end-systole; RWT= relative wall thickness; LVMI= left ventricular mass index; PSS= peak systolic stress at the midwall. The measurement and calculation of

above parameters were described in Supplementary Methods. Each data point represents the mean of an individual patient.

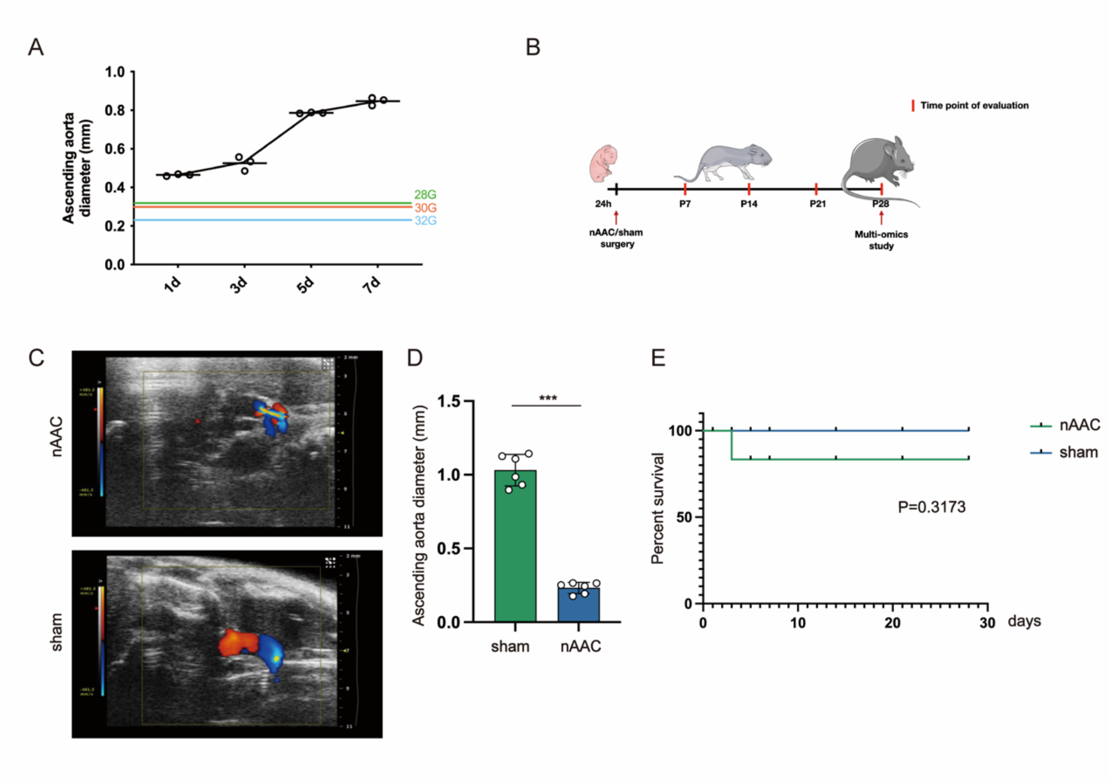

Figure S2. Construction of the nAAC mice model.

(**A**) A pilot echocardiography was conducted to measure the diameters of the ascending aortae in postnatal 1d, 3d, 5d, 7d C57/BL6 mice. 28G, 30G, and 32G needles could induce 50–70% constriction within 1–3 d after birth. The 28G needle constriction was selected to increase survival, prolong lifespan, and study chronic remodeling from birth to pre-adult. (**B**) The animal fate flowchart. The ascending aortae was surgically constricted in newborn mice within 24 h after birth and the model was evaluated at at different time points. (**C**) Representative Color Doppler mode images of ascending aortic blood flow in sham and TAC mice. (**D**) The diameter of ascending aorta in sham and TAC mice. (**E**) Survival curve of nAAC and sham mice. Unpaired Student t-test was used in **D**. Log-rank test was used in **E**. Each data point represents the mean of an individual animal. Data are presented as mean±SD. ***P<0.001.

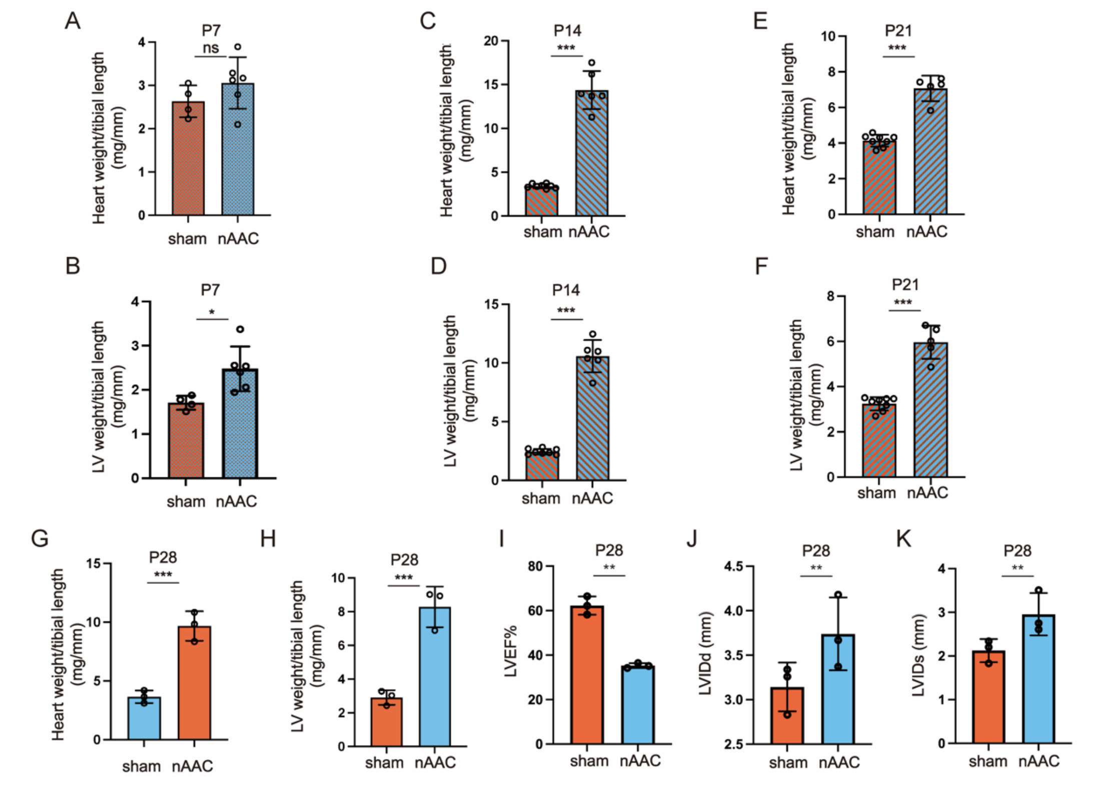

Figure S3. Gross and echo evaluation of the nAAC mice model.

(**A-H**) Gross evaluation of heart weight/tibial length of sham and nAAC mice at postnatal day 7 (**A**), day 14 (**C**), day 21 (**E**), day 28 (**G**) and LV weight/ tibial length at postnatal day 7 (**B**), day 14 (**D**), day 21 (**F**), day 28 (**H**). (**I**) LVEF% was measured by echocardiography at postnatal day 28 between sham and nAAC mice. (**J** and **K**) LV diameter was measured by echocardiography at postnatal day 28, including LVIDd (**J**) and LVIDs (**K**). LVIDd=left ventricular internal diameter at end-diastole; LVIDs=left ventricular internal diameter at end-systole. 2-tailed Mann-Whitney U test was used. Each data point represents the mean of an individual animal. Data are presented as mean ±SD. Ns=not significant; *P<0.05; **P<0.01; ***P<0.001.

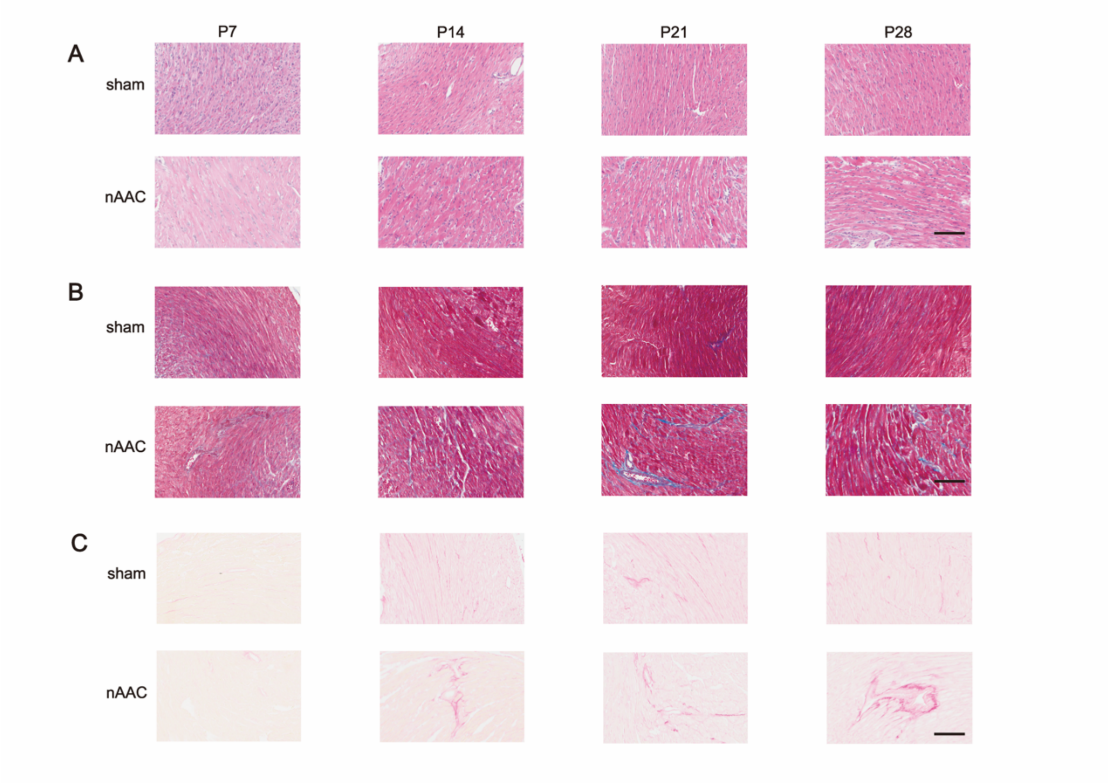

Fig. S4. Histological evaluation of nAAC mice model.

(**A**) Representative Hematoxylin-Eosin (HE) staining of left ventricle samples of sham and nAAC mice at postnatal day 7 (P7), day 14 (P14), day 21 (P21), day 28 (P28). n=5 per group. (**B**) Representative Masson staining showing the degree of fibrosis in sham and nAAC mice at different time points. n=5 per group. (**C**) Representative Sirius red staining indicating collagen deposition in sham and nAAC mice at different time points. Bar=100um. Representative images from individual animal.

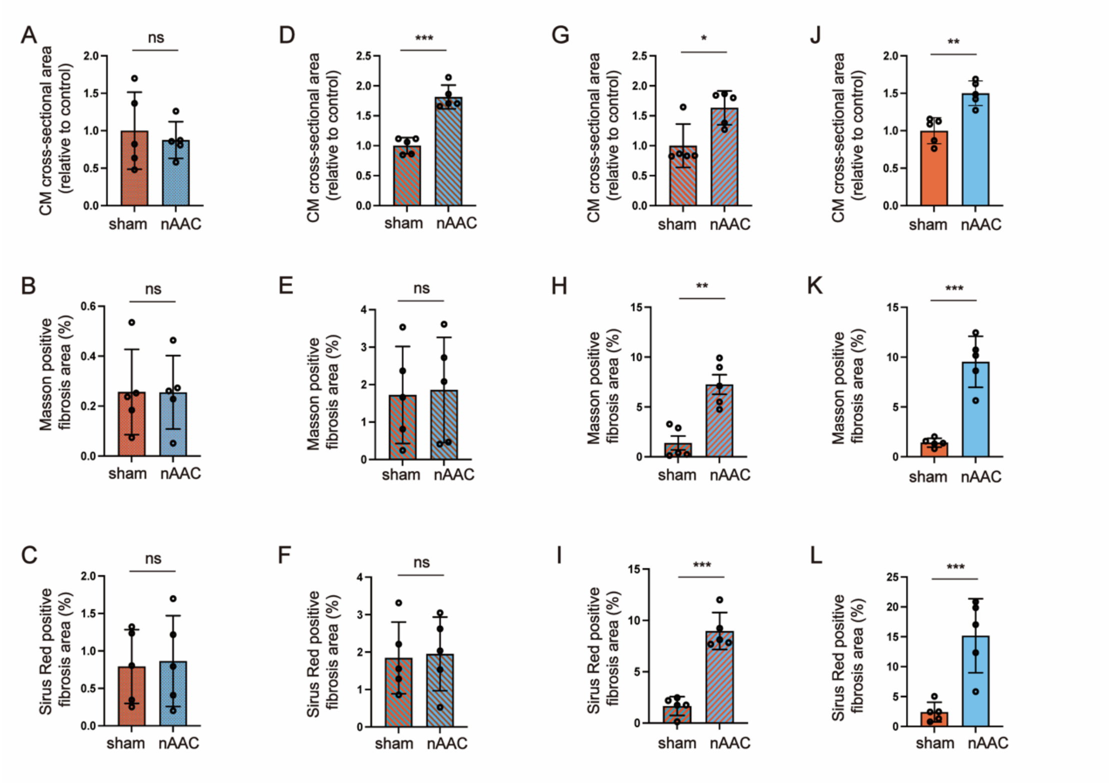

Fig. S5. Quantification of the histological staining of nAAC mice model.

(**A-C**) CM cross-sectional area (**A**), Masson fibrosis area (**B**) and Sirius red fibrosis area (**C**) of sham and nAAC mice at P7. n=5 per group. (**D-F**) CM cross-sectional area (**D**), Masson fibrosis area (**E**) and Sirius red fibrosis area (**F**) of sham and nAAC mice at P14. n=5 per group. (**G-H**) CM cross-sectional area (**G**), Masson fibrosis area (**H**) and Sirius red fibrosis area (**I**) of sham and nAAC mice at P21. n=5 per group. (**J-L**) CM cross-sectional area (**J**), Masson fibrosis area (**K**) and Sirius red fibrosis area (**L**) of sham and nAAC mice at P28. n=5 per group. 2-tailed Mann-Whitney U test was used. Each data point represents the mean of an individual animal. Data are presented as mean±SD. Ns=not significant; *P<0.05; **P<0.01; ***P<0.001.

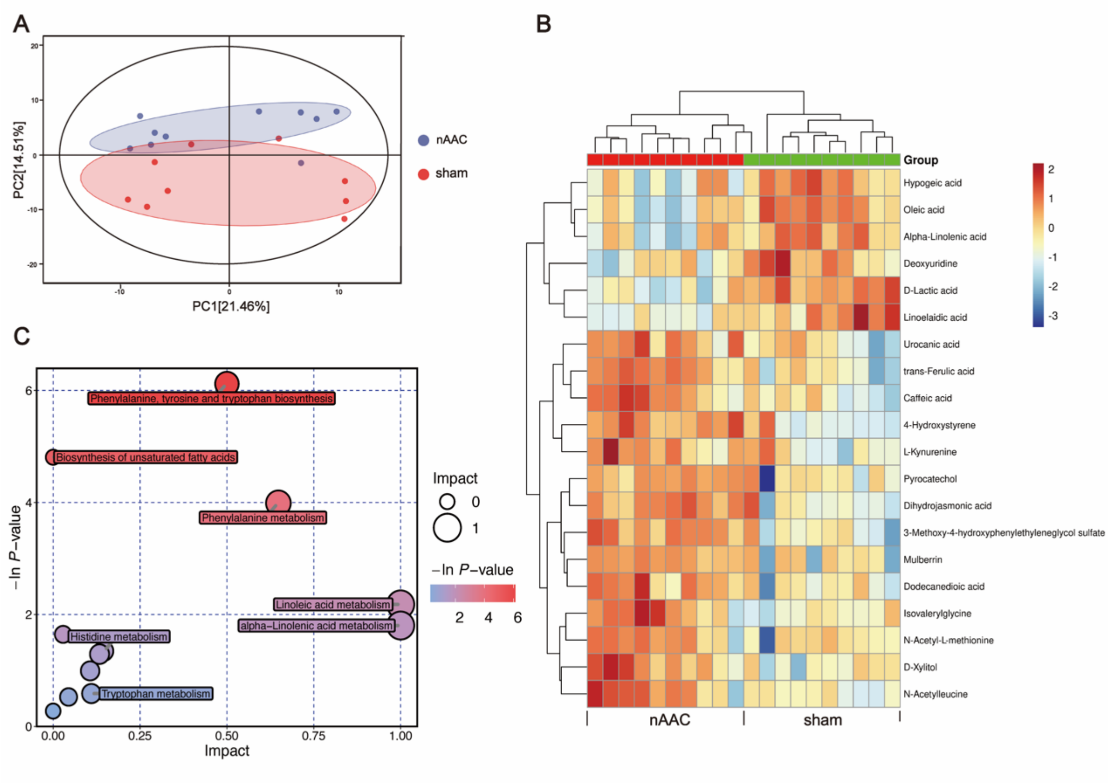

Fig. S6. Plasma metabolomics of nAAC mice and sham mice

(**A**) Untargeted metabolomics was conducted between the plasma of nAAC mice and sham mice (n=10 in each group). PCA illustrated substantial difference in the metabolites composition. (**B**) The heat map of differential plasma metabolites with top significance. (**C**) The metabolic pathway analysis of the differential metabolites. Phenylalanine, tyrosine and tryptophan biosynthesis pathways and tryptophan metabolism were enriched. Other metabolic pathway related to tryptophan, including serotonin pathway and indole pathway, and their intermediate metabolites were not significant.

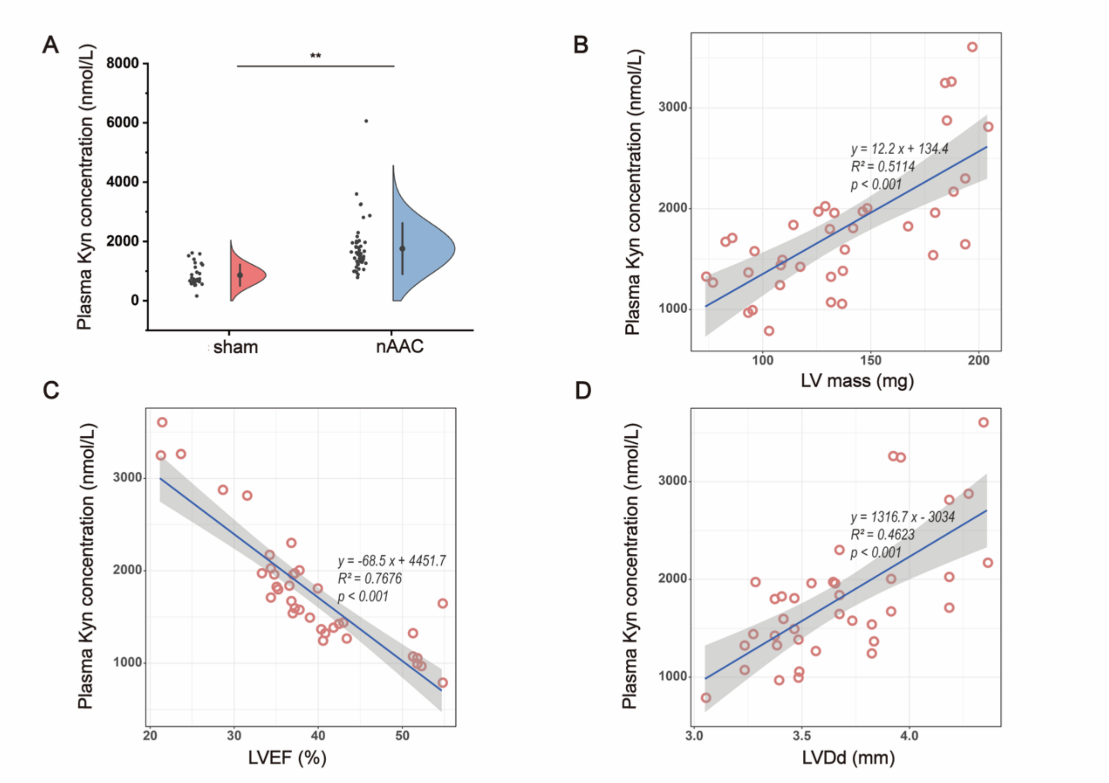

Fig. S7. Kyn is validated to be the key metabolites increased in nAAC mice

(**A**) UHPC-MS/MS was performed to quantify plasma Kyn between sham mice (n=29) and nAAC mice (n=51). (**B**) Linear regression showed correlation between Kyn and LV mass. (**C**) Linear regression showed correlation between Kyn and LVEF%. (**D**) Linear regression showed correlation between Kyn and LVDd. Each data point represents the mean of an individual animal. Student t-test was used in **A**. **P<0.01.

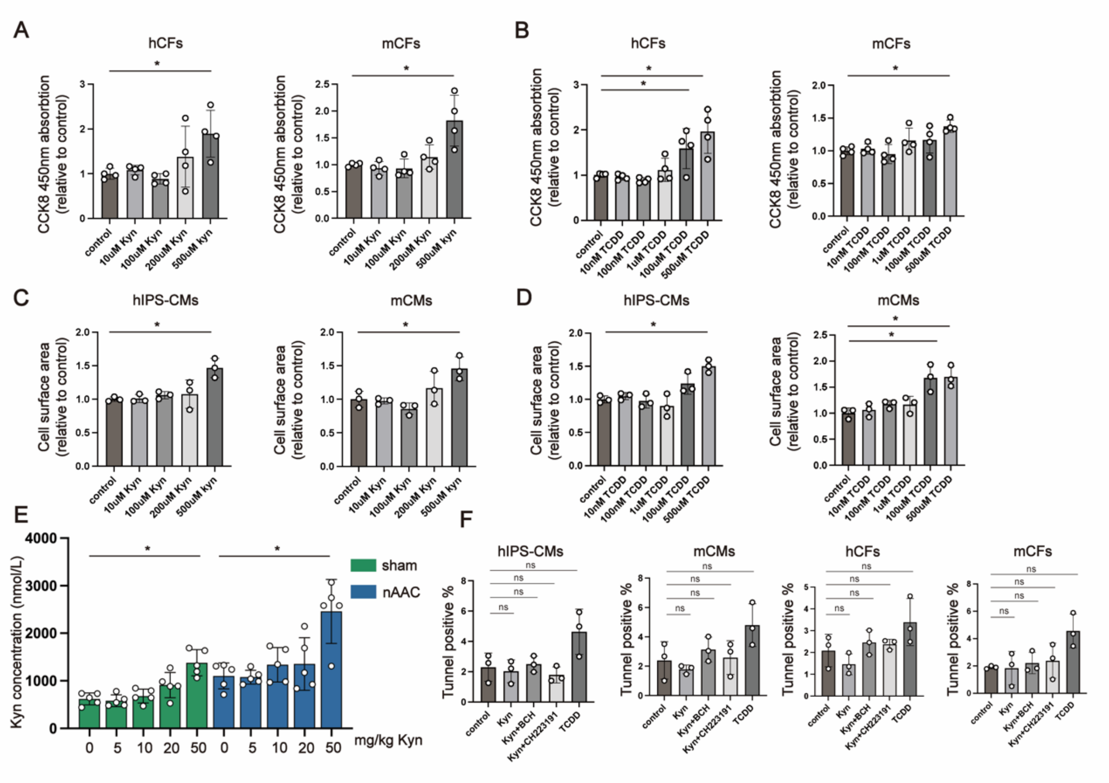

Fig. S8. Preliminary dose exploration for cell and mice studies.

(**A-B**) CCK8 activity in hCFs and mCFs treated with different doses of Kyn (**A**) and TCDD (**B**). 500μM Kyn and TCDD would induce stable increase of activity in both cells. n=3 per group. (**C-D**) Cell surface area of hIPS-CMs and mCMs treated with different doses of Kyn (**C**) and TCDD (**D**). 500μM Kyn and TCDD caused significant hypertrophy in both cells. n=3 per group. (**E**) Plasma Kyn concentration of WT sham and WT nAAC mice after daily peritoneal injections of different doses of Kyn for 4 wks. n=5 per group. (**F**) Tunnel assay to detect cell apoptosis in hIPS-CMs, mCMs, hCFs and mCFs treated with DMSO, 500 μM Kyn, 500 μM Kyn and 500 μM BCH, 500 μM Kyn and 500 μM CH223191, 500 μM TCDD. The above treatments induced cell death in acceptable ranges. n=3 per group. Each data point represents the mean of an individual animal. Kruskal-Wallis test and post hoc Dunn test were used. Ns=not significant; *$P<0.05$.

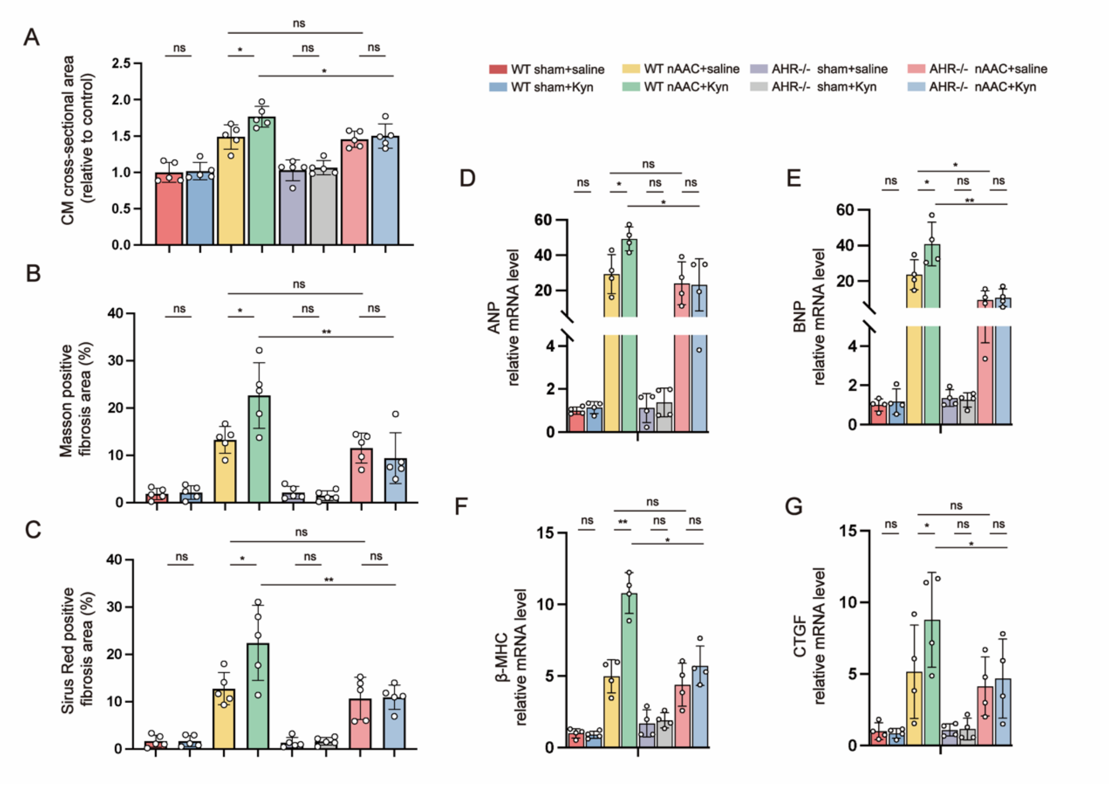

Fig. S9. Increased Kyn and AHR activation aggravate histological and molecular remodeling

(**A-C**) CM cross-sectional area (**A**), Masson fibrosis area (**B**) and Sirius red fibrosis area (**C**) of LV samples in according groups in Fig.3H. n=5 per group. (**D-G**) qPCR analysis of the relative mRNA expression of cardiac function indicators *ANP* (**D**) and *BNP* (**E**), hypertrophy marker *b-MHC* (**F**) and fibrosis marker *CTGF* (**G**) of LV tissues in three groups. n=4 per group. Each data point represents the mean of an individual animal. Kruskal-Wallis test and post hoc Dunn test were used. ns= not significant; *P<0.05; **P<0.01

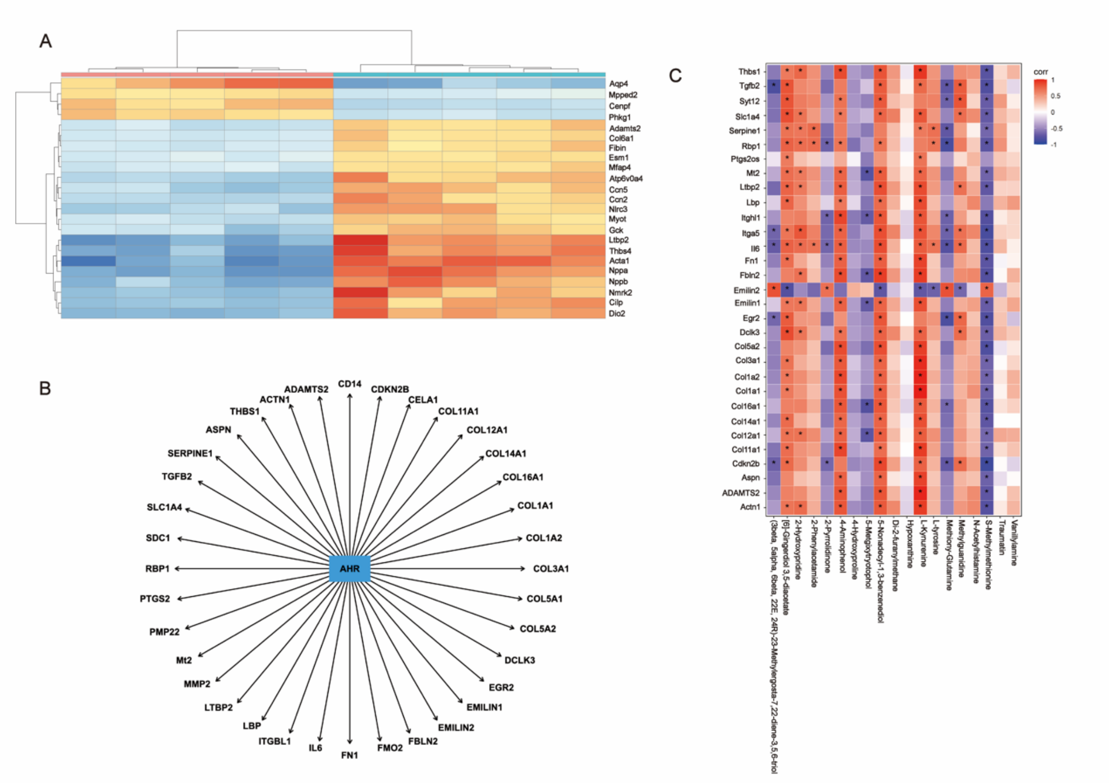

Fig. S10. RNA sequencing and bioinformatics analysis of LV samples between nAAC and sham mice identifies remodeling genes associated with Kyn-AHR axis. (**A**) Heat map of differential genes between LV samples of sham (Z1-Z5) and nAAC mice (X1-X5). n=5 per group. (**B**) Ingenuity Pathway analysis (IPA) filtered the differential genes that were regulated by AHR. (**C**) Correlation analysis between untargeted metabolomics of plasma and RNA sequencing of LV samples revealed strong correlation between Kyn level and *COL1A1, FN1, ADAMTS2*.

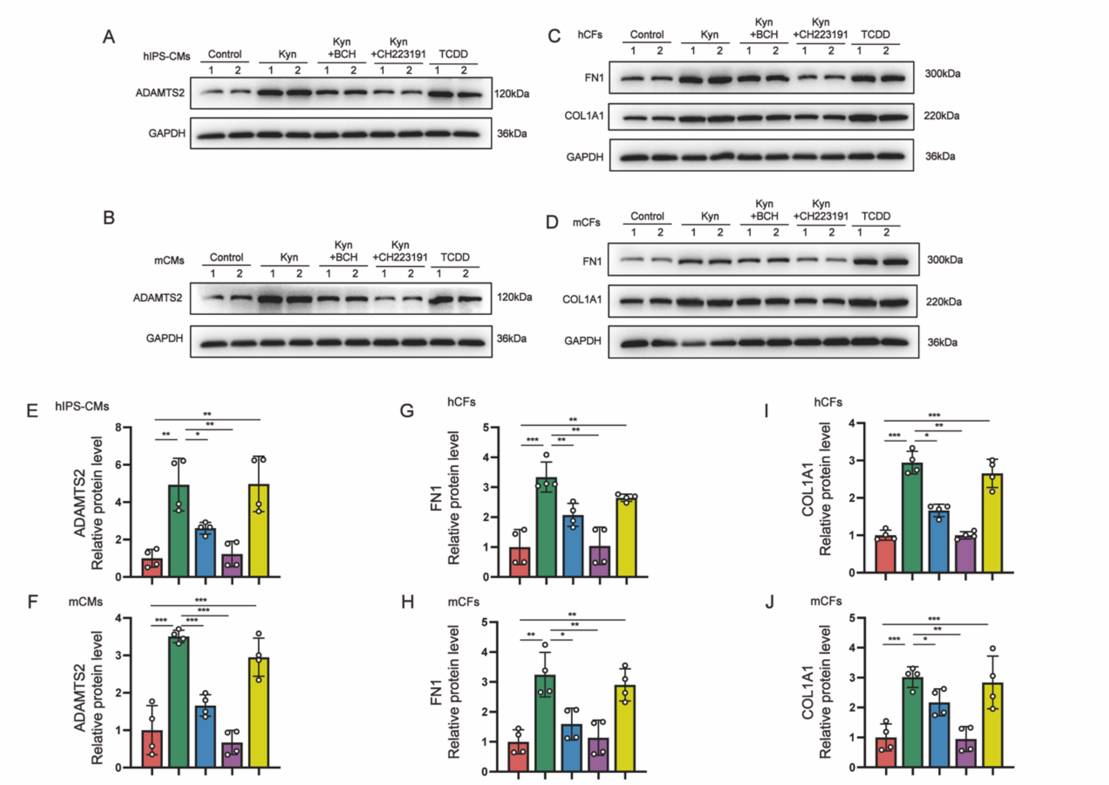

Fig. S11. Biological repetition and quantification of the western blotting in manuscript Figure 4H-K.

(**A**-**D**) Biological repetition of Fig.4H-K. Representative western blotting of the protein expression ADAMTS2 in hIPS-CMs (**A**) and mCMs (**B**), FN1 and COL1A1 in hCFs (**C**) and mCFs (**D**) treated with indicated compounds. Each lane represented individual experiment in vitro. (**E-J**) Quantification of the western blotting of ADAMTS2 in hIPS-CMs (**E**) and mCMs (**F**), FN1 in hCFs (**G**) and mCFs (**H**), COL1A1 in hCFs (**I**) and mCFs (**J**). n=4 per group. Kruskal-Wallis test and post hoc Dunn test were used. Each point represents an individual biological repeat. ns= not significant; *$P<0.05$; **$P<0.01$, ***$P<0.001$.

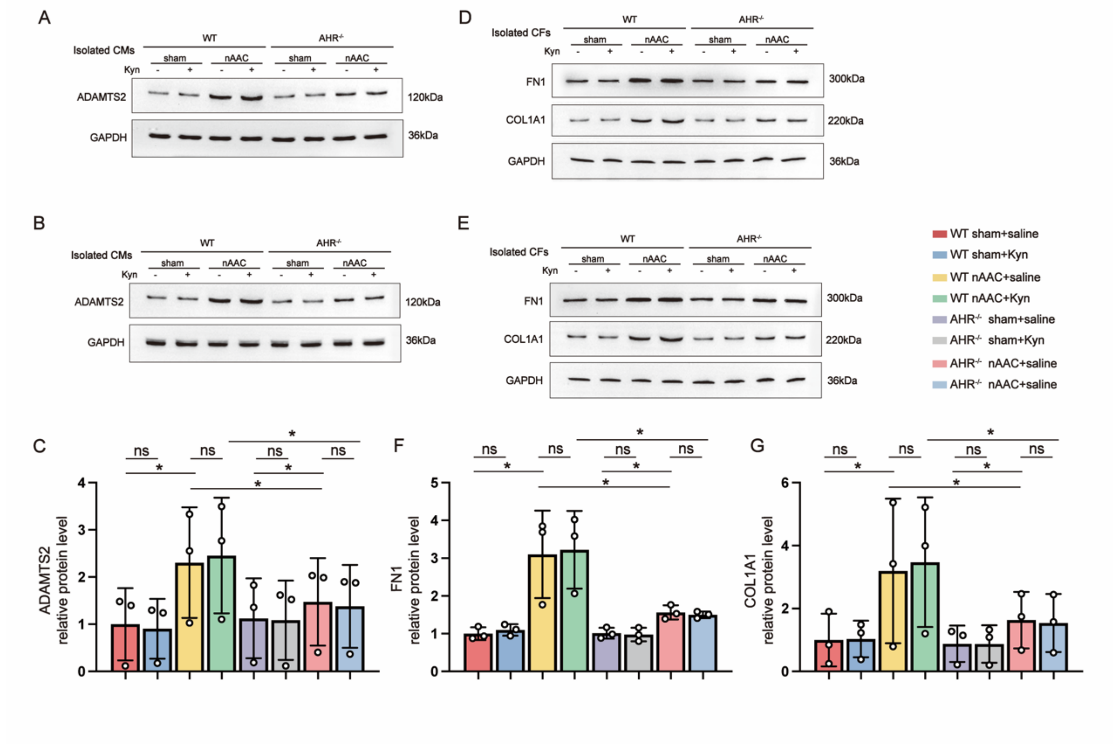

Fig. S12. Biological repetition and quantification of the western blotting in manuscript Figure 4N and 4O.

(**A**-**B**) Representative western blotting of ADAMTS2 in isolated mCMs from WT and *AHR*$^{-/-}$ mice 4 weeks after sham or nAAC surgery, with Kyn peritoneal injection or saline as control. (two biological repetition of **Figure 4N**). (**C**) Quantification of the western blotting of ADAMTS2 in isolated mCMs from indicated mice. (**D**-**E**) Representative western blotting of FN1 and COL1A1 in isolated mCFs from indicated mice (two biological repetition of **Figure 4O**). (**F**-**G**) Quantification of the western blotting of FN1 (**F**) and COL1A1 (**G**) in isolated mCFs from indicated mice. Kruskal-Wallis test and post hoc Dunn test were used. Each point represents the mean of an individual animal. ns= not significant; *P<0.05; **P<0.01.

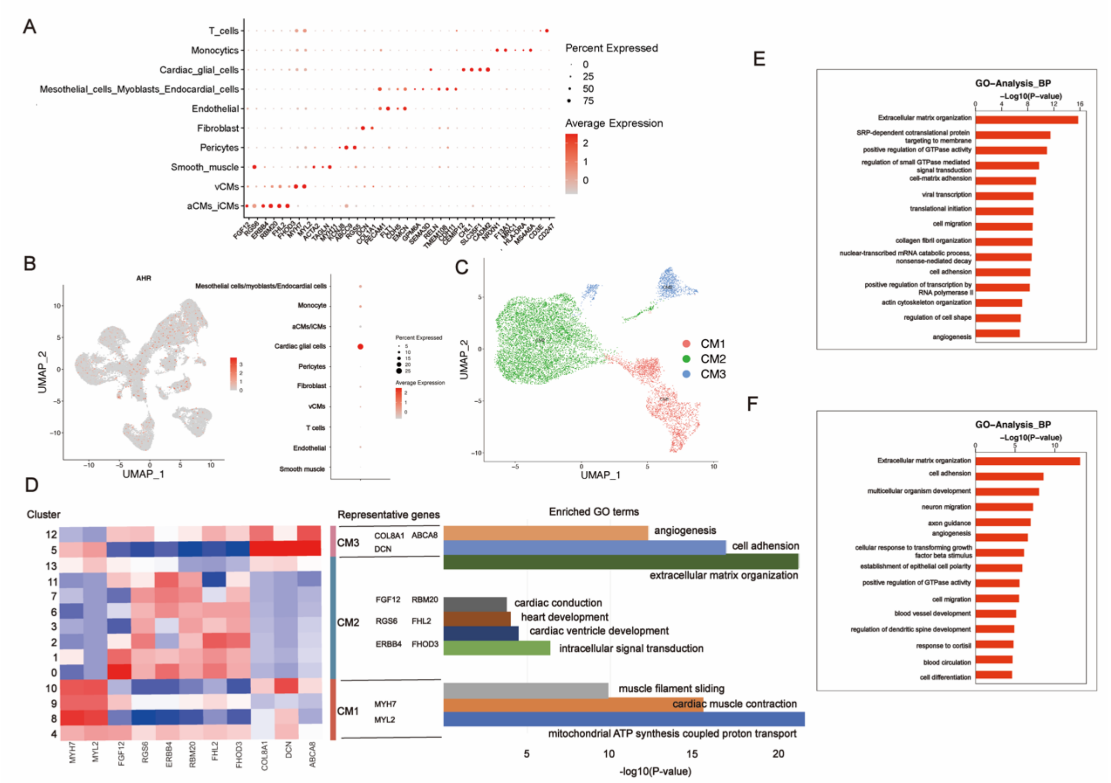

Fig. S13. Clustering of single-nucleus sequencing and cardiomyocytes subclusters.

(**A**) Bubble plot showing the normalized mean expression of marker genes in each cell types. (**B**) UMAP projection of *AHR* distribution and *AHR* expression in cell types. (**C**) UMAP projection of CMs types. (**D**) Heat map of the representative genes of each CMs subclusters. The subclusters were further determined into three functional types, CM1=cluster 4, 8, 9, 10; CM2=cluster 0, 1, 2, 3, 6, 7, 11, 13; CM3=5,12. Top enriched GO terms showed their biological process they involved in. (**E**) GO analysis of biological process of subcluster 5. (**F**) GO analysis of biological process of subcluster 12. BP=biological process.

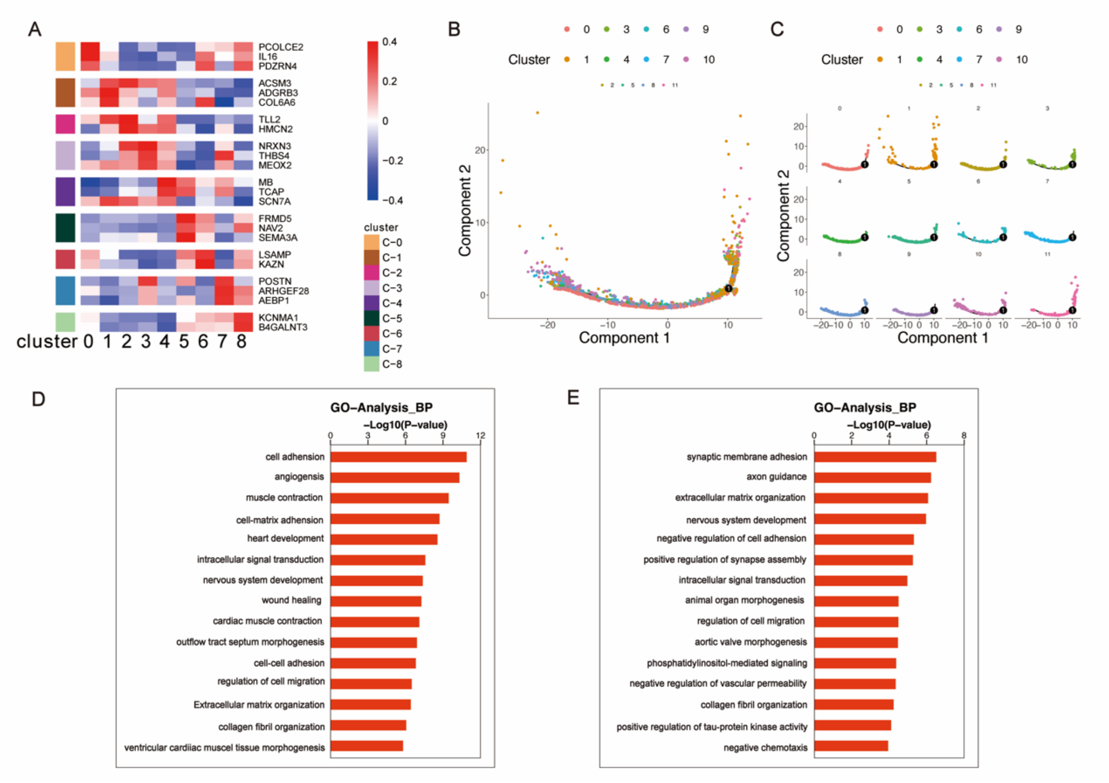

Fig. S14. CFs subclusters in single-nucleus sequencing.

(**A**) Heat map of the marker genes in the clustering of CFs. (**B-C**) pseudo-time analysis of CF subclusters, illustrated by the whole samples (**B**) and by each subcluster (**C**). (**D**-**E**) GO analysis of biological process of CFs subcluster 5 (**D**) and 6 (**E**).

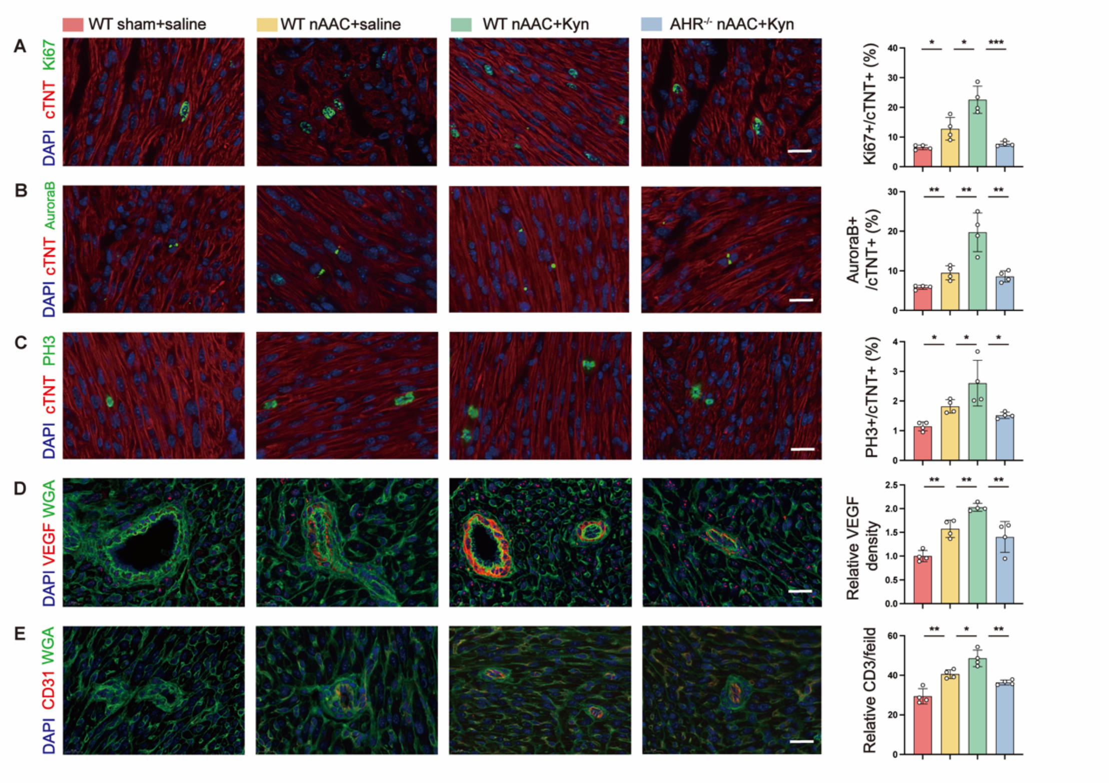

Fig. S15. nAAC and Kyn promote cardiac regeneration and angiogenesis in P7 mice

(**A-B**) Representative immunofluorescence staining and quantification of LV sections for Ki67 (**A**) or Aurora B (**B**) or PH3 (**C**), and cTNT in P7 indicated mice. n=4 per groups. (**D-E**) Representative immunofluorescence staining and quantification of LV sections for VEGF (**D**) or CD31 (**E**), and WGA in P7 indicated mice. n=4 per groups. Bar=20μm. Kruskal-Wallis test and post hoc Dunn test were used. Each point represents the mean of an individual animal. ns= not significant; *P<0.05; **P<0.01, ***P<0.001.

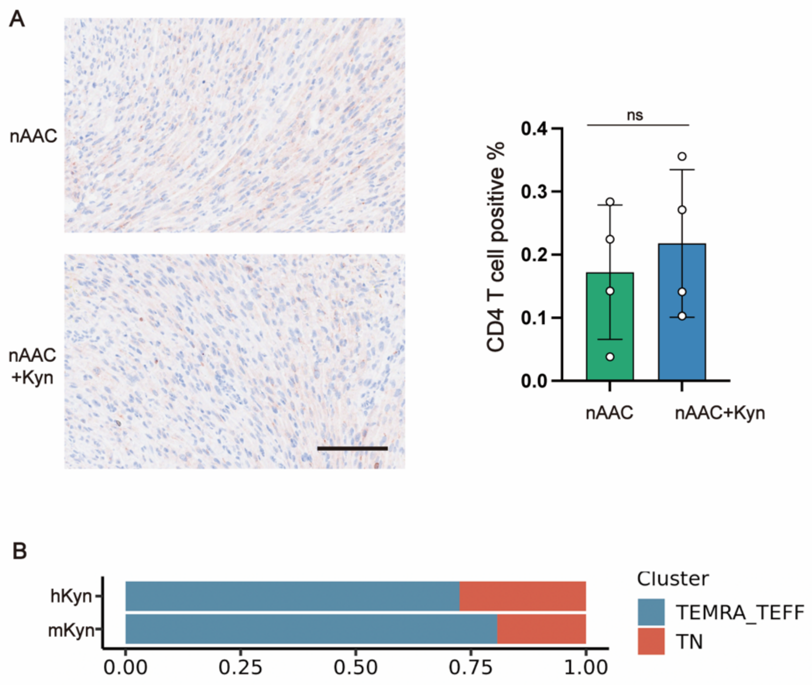

Fig. S16. Assessment of T cell in nAAC mice and snRNA-seq

(**A**) Representative immunostaining of $CD4^+$ T cell and quantification of LV sections from 4-wks age nAAC and nAAC mice with 50mg/kg Kyn injection. n=4 per group. (**B**) Cell proportion of T cell subclusters in snRNA seq between hKyn and mKyn groups. TN = Naive T cell; TEMRA/TEFF= Recently activated effector memory or effector T cells. 2-tailed Mann-Whitney U test was used in **A**. Each point represents the mean of an individual animal. ns= not significant.

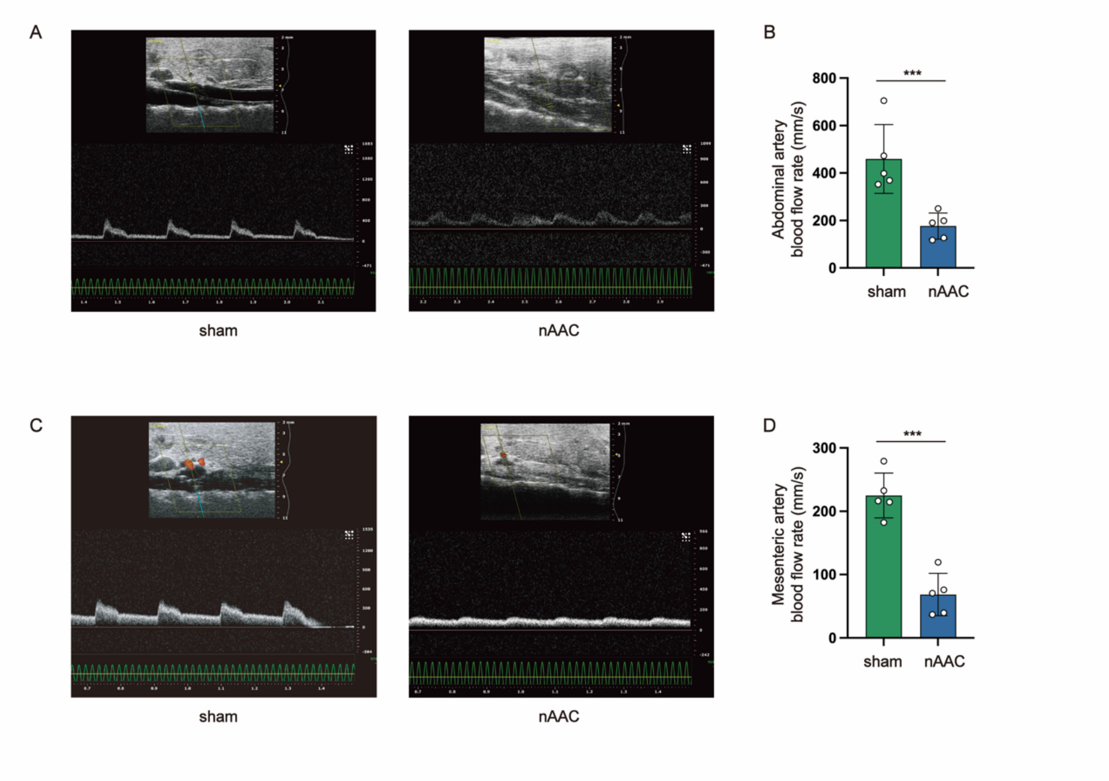

Fig. S17. Ultrasonography of abdominal aortae and mesentery artery

(**A**) Representative ultrasonography image and Doppler velocity waveform of abdominal aortae of sham mice and nAAC mice at 4-week age. (**B**) Quantification of abdominal artery blood flow rate in sham and nAAC mice at 4-week age. n=5 per group. (**C**) Representative ultrasonography image and Doppler velocity waveform of mesentery arteries of sham mice and nAAC mice at 4-week age. (**B**) Quantification of mesentery arteries blood flow rate in sham and nAAC mice at 4-week age. n=5 per group. Unpaired Student-t test were used. Each point represents the mean of an individual animal. ns= not significant; *P<0.05; **P<0.01, ***P<0.001.

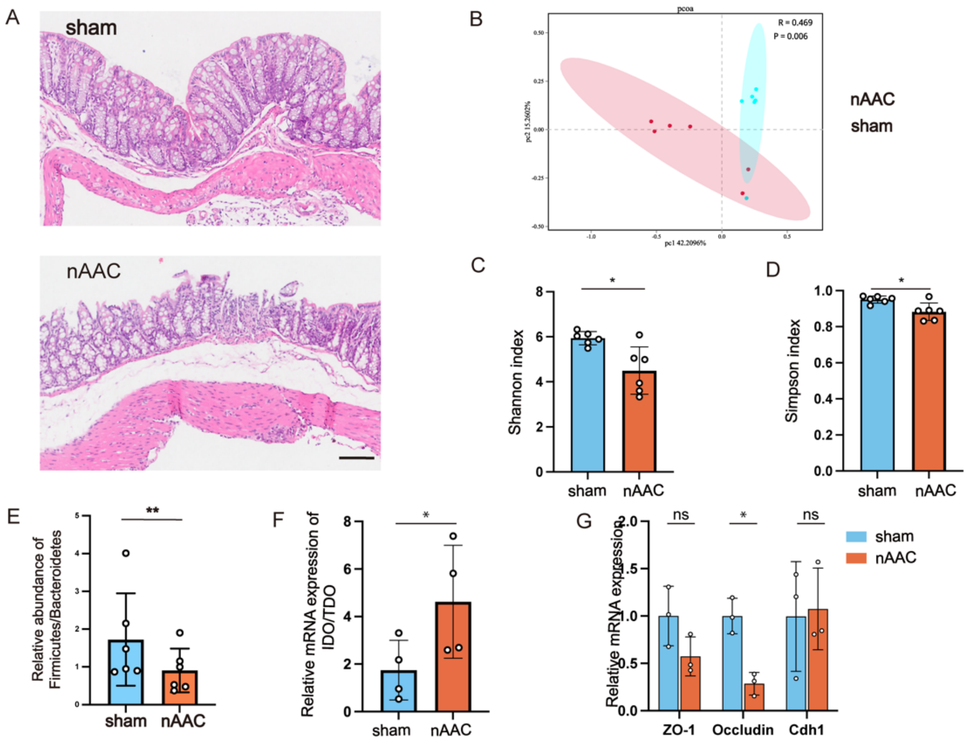

Fig. S18. Intestinal disruption and gut microbiota alteration in nAAC mice

(**A**) Representative HE staining of the colons from 4-week age sham and nAAC mice. Bar=100μm. (**B**) Principal coordinates analysis (PCoA) of 16S RNA sequencing of the feces from 4-week age sham and nAAC mice (n=6 in each group), indicating a great difference between their gut microbiota composition. The beta-diversity metric was Bray Curtis and PERMANOVA was used for statistical test (R^2=0.469, P=0.006). (**C-D**) Alpha diversity indicated by Shannon index (**C**) and Simpson index (**D**) between sham and nAAC group. n=6 per group. (**E**) The ratio of Firmicutes/Bacteroidetes abundance between sham and nAAC group. n=6 per group. (**F**) The relative mRNA ratio of *IDO*/*TDO* enzymes between sham and nAAC colon. n=4 per group. (**G**) The relative mRNA expression of intestinal integrity markers *ZO-1*, *Occludin*, *Cdh1* between 4-wks age sham and nAAC colon. n=3 per group. 2-tailed Mann-Whitney U

test was used in **E-G**. Data are presented as mean±SD. ns= not significant; *P<0.05; **P<0.01.

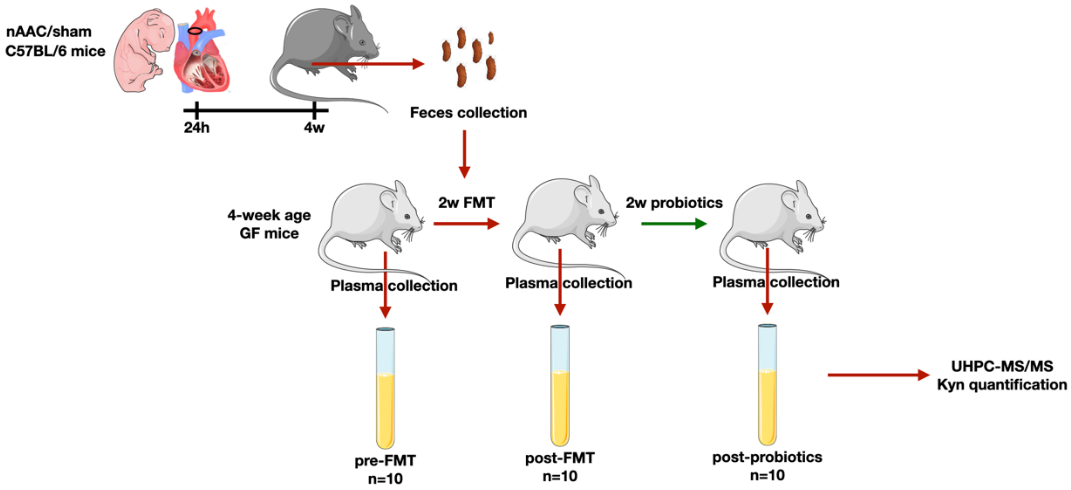

Fig. S19. Schematic of FMT strategies on GF mice.

Fresh fecal pellets were collected from donor nAAC or sham C57BL/6 mice (5 male and 5 female per group) at the 4th week after surgery in an empty, sterile plastic cage. Then fecal supernatant was prepared in a germ-free working hood. To examine the relationship between Kyn and nAAC gut microbiota (**Figure 6B)**, before first FMT, blood samples were firstly collected in 4-week age GF mice (pre-FMT, n=10). Then GF mice were gavaged with 0.015ml/g fecal solution daily for 2 weeks, followed by a second blood collection (post-FMT, n=10). For evaluation of the effect of probiotics (**Figure 6I**), the post-FMT GF mice were then gavaged daily with 0.015ml/g probiotics slurry for 2 weeks, and the third blood collection was conducted (post-probiotics, n=10). The blood samples were all centrifuged into plasma immediately and used for UHPC-MS/MS quantification of Kyn.

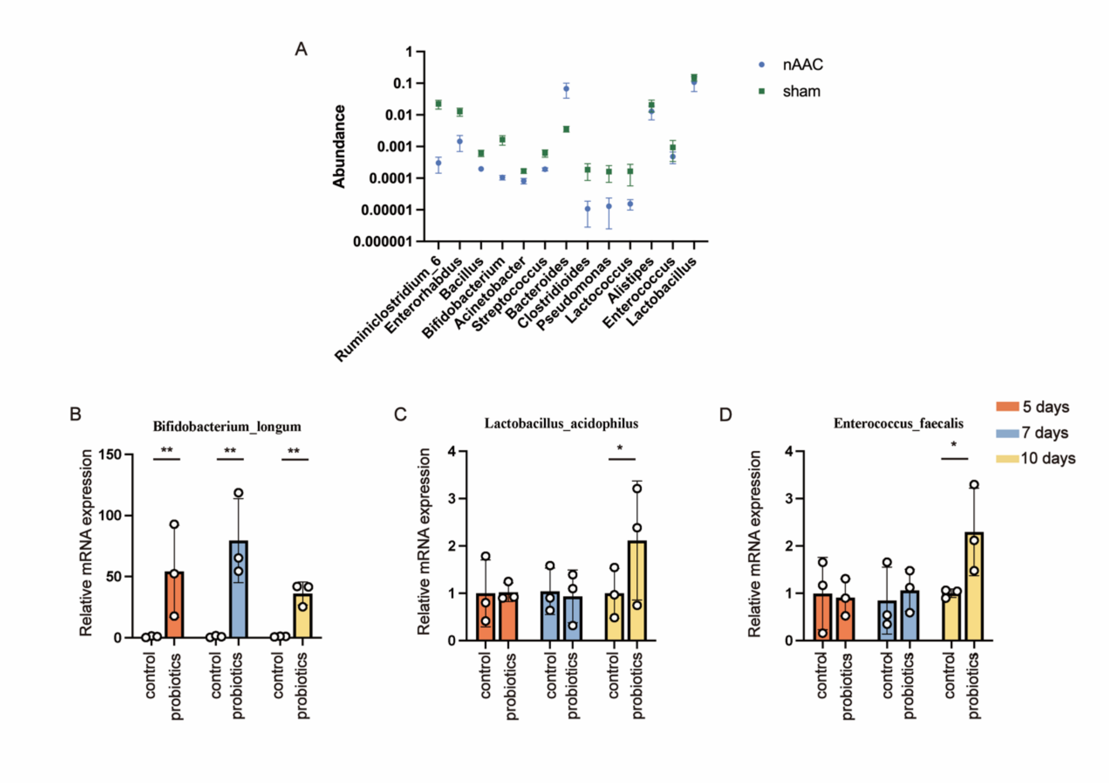

Fig. S20. Pilot studies to select probiotic species and evaluate the efficacy of their oral administration

(**A**) To choose the probiotics species for oral supplement, the top decreased microbes in nAAC gut were analyzed at genus level in 16S sequencing. *Bifidobacterium_longum, Lactobacillus_acidophilus,* and *Enterococcus_faecalis* were chosed for probiotics treatment of nAAC mice. (**B-D**) qPCR quantification of the mRNA level of *Bifidobacterium_longum* (**B**), *Lactobacillus_acidophilus* (**C**), *Enterococcus_faecalis* (**D**) in mice colon content after oral probiotics supplement for 5, 7, and 10 days. n=3 per group. 2-tailed Mann-Whitney U test was used. Each point represents the mean of an individual animal. *P<0.05; **P<0.01.

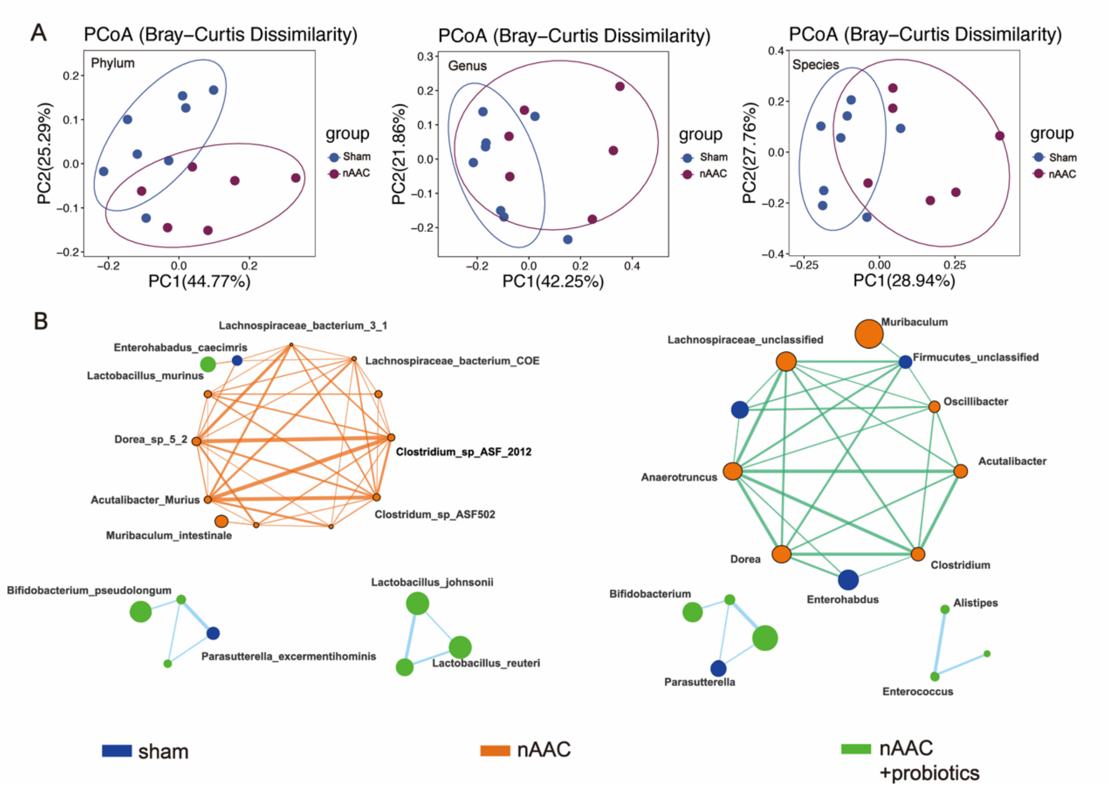

Fig. S21. Gut microbiota composition difference and interaction network.

(**A**) PCoA by Bray-Curtis distance at genus, phylum and species level in metagenomics sequencing between the feces of sham (n=8) and nAAC (n=6). PERMANOVA test was used for statistical analysis (phylum level $R^2 = 0.244$, $p = 0.008$; genus level $R^2 = 0.245$, $p = 0.009$; species level $R^2 = 0.206$, $p = 0.007$). (**B**) Microbes' interaction and correlation network at genus (left) and species (right) level in metagenomics sequencing among the feces of sham mice, nAAC mice, nAAC mice with probiotics. Noxious microbes in nAAC groups (shown in orange), such as *Anaerotruncus_sp_G3_2012*, *Clostridium_sp_ASF502*, *Acutalibacter_muris*, *Lachnospiraceae_bacterium_COE1/3_1* were strongly correlated and mutually promoted the growth of with each other. Meanwhile, the beneficial microbes were also correlated. Introducing *Enterorhabdus_caecimuris* and *Lactobacillus_murinus,* as

supplied in probiotics group, into nAAC dysbiosis environment might be a key point to suppress and break this correlation.

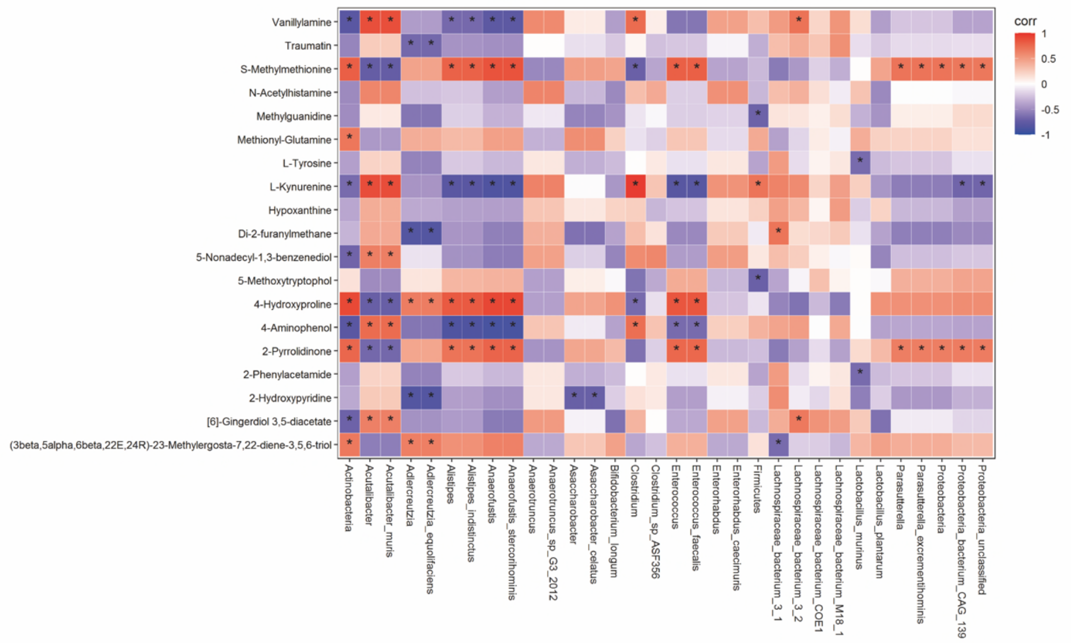

Fig. S22. Correlation analysis of two omics study: gut microbiota metagenomics and plasma metabolomics.

Conjoint analysis of between metagenomics and untargeted metabolomics in sham (n=8), nAAC (n=6) and nAAC mice with probiotics (n=8), illustrating positive correlations between plasma Kyn and *Acutalibacter_muris*, *Clostridium*, and *Firmicutes*. And negative correlations between Alistipis_indistinctus, *Anaerofustis_stercorihominis*, *Enterococcus_Faecalis* and Kyn. Plasma and feces were collected from the same mice. *P<0.05.

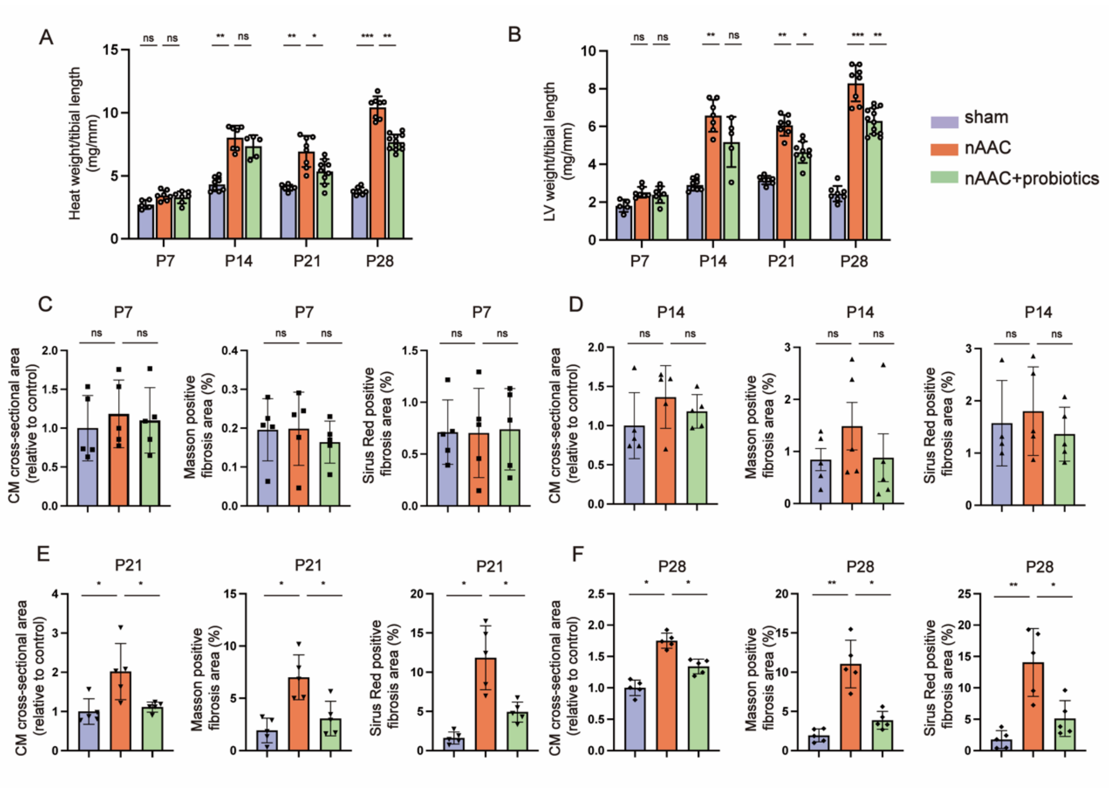

Fig. S23. Gross evaluation and histological quantification of heart remodeling to verify the protective effect of probiotics.

(**A**-**B**) At postnatal 7d, 14d, 21d, 4w, gross evaluation of heart weight/tibial length (**A**) and LV weight/tibial length (**B**) was measured in sham mice, nAAC mice and nAAC mice supplied with probiotics. (**C**-**F**) CM cross-sectional area, Masson fibrosis area and Sirius red fibrosis area of indicated mice at P7 (**C**), P14 (**D**), P21 (**E**), P28 (**F**). n=5 per group. Kruskal-Wallis test and post hoc Dunn test were used. Each point represents the mean of an individual animal. *P<0.05; **P<0.01.

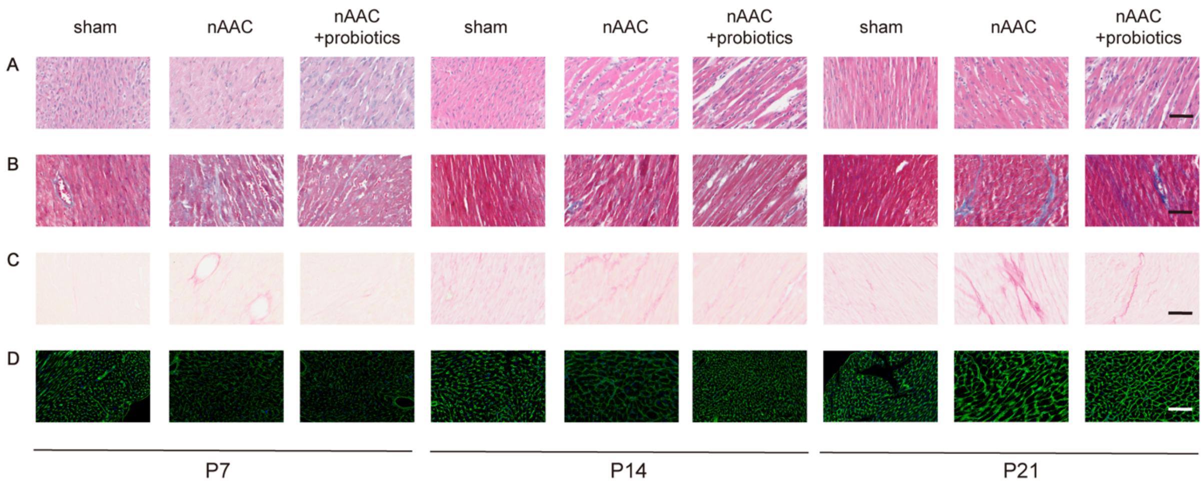

Fig. S24. Histological evaluation of heart remodeling to verify the protective effect of probiotics.

A, Representative histological staining of LV samples in sham mice, nAAC mice and nAAC mice at postnatal day 7 (P7), day 14 (P14) and day21 (P21), indicating the degree of myocardial hypertrophy. **B** and **C**, Representative Masson staining (**B**) and Sirius red staining (**C**) of LV samples in three groups of different time points, showing the degree of fibrosis. **D**, Representative WGA staining of LV samples in three groups, showing cross-sectional area. Bar=50μm.Representative images from individual animal (n=5 in each group).

Supplemental Tables

Table S1. Patients' characteristics of Discovery cohort for untargeted metabolomics.

	poLV group (n=10)	Control group (n=10)	P value
Age (month)	31.2±22.04	22.2±20.43	0.357
Male	5	4	1.000
Weight (kg)	12.55±6.63	10.77±5.63	0.526
EF (%)	56.16±9.48	73.34±2.73	**0.000**
FS (%)	30.88±7.52	41.41±2.95	**0.001**
Z scores			
LVIDd	-0.46±1.54	-0.24±1.25	0.734
LVIDs	-0.43±1.55	0.10±1.17	0.399
IVSd	2.81±1.22	1.09±0.72	**0.001**
LVPWd	1.92±1.37	0.77±0.85	**0.000**
LVPWs	0.41±4.72	-0.93±1.73	0.744
RWT	0.52±0.11	0.34±0.05	**0.000**
LVMI (g/m^2)	129.82±74.78	63.48±24.45	**0.016**
PSS (g/cm^2)	96.27±15.39	144.74±46.08	**0.005**
Diagnosis	AS (2)	Small ASD (6)	
	CoA (3)	Small VSD (4)	
	LVOTO-SAS (5)		

AS=aortic stenosis; SAS= subvalvular aortic stenosis; CoA=coarctation of aorta; LVOTO=left ventricular outflow tract obstruction; ASD=atrial septum defect; VSD=ventricular septum defect; EF=ejection fracture; FS= fraction shortening; LVIDd=left ventricular internal diameter at end-diastole; LVIDs=left ventricular internal diameter at end-systole; IVSd= internal diameter of septum at end-diastole; LVPWd= left ventricular posterior wall thickness at end-diastole; LVPWs= left ventricular posterior wall thickness at end-systole; RWT= relative wall thickness;

LVMI= left ventricular mass index; PSS= peak systolic stress at the midwall. Statistical significance was determined by the Student t test. P<0.05 was shown as bold.

Table S2. Top significantly different metabolites in untargeted metabolomics in plasma of patients' discovery cohort.

Metabolites	Adjusted P value	VIP
Pseudouridine	0.000239105	2.273422969
N-Acetyl-L-alanine	0.000656017	2.259865542
L-Kynurenine	0.0000252023	2.252261761
3-Methoxy-4-hydroxyphenylethyleneglycol	0.000855865	2.216673142
sulfate	0.007535893	2.145205115
[10]-Dehydrogingerdione	0.000543988	2.18305129
Succinic acid	0.001969572	2.156951241
D-Alanine	0.007590575	2.145668149
Dodecanedioic acid	0.003540494	2.075095289
4-Hydroxyproline	0.003375561	2.062096831
N2,N2-Dimethylguanosine	0.000799532	2.0293532
Trehalose	0.002680842	2.009495443
1H-Indole-3-carboxaldehyde	0.039663595	1.965120806
Aminoadipic acid	0.013623378	1.943705117
9-Decenoic acid	0.04389078	1.939504233
Paliperidone	0.001087805	1.935052232
N4-Acetylcytidine	0.042417191	1.930177444
(R)-3-Hydroxy-tetradecanoic acid	0.039663595	1.965120806
Aminoadipic acid	0.013623378	1.943705117
9-Decenoic acid	0.04389078	1.939504233
Paliperidone	0.001087805	1.935052232
N4-Acetylcytidine	0.042417191	1.930177444
(R)-3-Hydroxy-tetradecanoic acid	0.022182653	1.90199135
4-Hydroxy tolbutamide	0.017646113	1.877889208
Tetradecanedioic acid	0.008452836	1.869432406
Aldehydo-D-xylose	0.009030136	1.859902444
2-Indolecarboxylic acid	0.00169077	1.848516482
Hexylresorcinol		

Significantly difference was determined as adjusted P value<0.05, VIP>1. Adjusted P values were calculated from P values corrected by false discovery rate (FDR) based on Benjamini-Hochberg method. Other kynurenine pathway metabolites were not significant, including kynurenic acid (KynA), Kyn/KynA ratio, quinolinic acid and 3-hydroxykynurenine.

Table S3. Patients' characteristics of Validation cohort for HPLC-MS/MS quantification of Kyn.

	poLV group (n=19)	Control group (n=25)	P value
Age (month)	41.64±56.98	20.12±25.75	0.100
Male	9	13	1.000
Weight (kg)	16.19±18.17	11.51±11.94	0.309
EF (%)	64.47±9.99	69.59±4.87	**0.031**
FS (%)	33.81±7.23	37.94±3.82	**0.019**
Z scores			
LVIDd	-0.29±2.61	-0.25±1.26	0.950
LVIDs	-0.10±2.48	0.15±1.22	0.666
IVSd	2.52±1.39	1.24±0.64	**0.000**
LVPWd	2.43±2.04	0.41±1.01	**0.000**
LVPWs	0.08±3.69	-1.10±1.35	0.147
RWT	0.60±0.46	0.36±0.06	**0.011**
LVMI (g/m^2)	138.02±84.41	67.57±20.95	**0.000**
PSS (g/cm^2)	92.66±16.91	147.72±43.26	**0.000**
Diagnosis	AS (6)	Small ASD (11)	
	CoA (8)	Small VSD (14)	
	LVOTO-SAS (5)		

AS=aortic stenosis; SAS= subvalvular aortic stenosis; CoA=coarctation of aorta; LVOTO=left ventricular outflow tract obstruction; ASD=atrial septum defect; VSD=ventricular septum defect; EF=ejection fracture; FS= fraction shortening; LVIDd=left ventricular internal diameter at end-diastole; LVIDs=left ventricular internal diameter at end-systole; IVSd= internal diameter of septum at end-diastole; LVPWd= left ventricular posterior wall thickness at end-diastole; LVPWs= left ventricular posterior wall thickness at end-systole; RWT= relative wall thickness; LVMI= left ventricular mass index; PSS= peak systolic stress at the midwall. Statistical significance was determined by the Student t test. $P<0.05$ was shown as bold.

Table S4. Top significantly different metabolites in untargeted metabolomics in plasma of nAAC mice vs sham mice.

Metabolites	Adjusted P value	VIP
Choline	0.003324282	2.163668897
Daidzein	0.029913955	1.813829904
1-Methyladenine	0.000970463	2.208707923
Genistein	0.02577832	1.49034821
Methylguanidine	0.001436018	2.216673142
Thiamine	0.001063501	2.145205115
L-Phenylalanine	0.002389946	2.414604077
Glycitein	0.00874687	1.873524159
5-Methoxytryptophol	0.024340836	2.242519565
[6]-Gingerdiol 3,5-diacetate	0.000249435	2.560226939
5-Heneicosyl-1,3-benzenediol	0.006584779	2.67260552
Propionylcarnitine	0.011848335	1.866288622
4-Guanidinobutanoic acid	0.000296431	2.14416416
2-Hydroxypyridine	0.023600495	2.19215422
5-Nonadecyl-1,3-benzenediol	0.001517178	2.874543352
Ethyl 3-methyl-9H-carbazole-9-carboxylate	0.003349151	2.112100901
Trimethylaminoacetone	0.025676562	2.008469227
4-Aminophenol	0.014223729	1.377127373
DL-2-Aminooctanoic acid	0.000221273	2.119462072
Campesterol	0.001251706	2.273358701
Phenylacetylglycine	0.006882984	2.038572239
SM(d18:1/16:0)	0.002599564	2.245905541
L-Kynurenine	0.006937335	2.374734458
Kynurenic acid	0.028246787	1.415333073
Octadecylamine	0.015105075	2.051918224
Glycerophosphocholine	0.027505365	1.573495075
Butyrylcarnitine	0.005206319	2.467314072
Nonivamide	0.048644974	1.85094423
4-Methylbenzaldehyde	0.025866602	1.729023334
4,6-Dihydroxyquinoline	0.010371118	2.431777479
Methylimidazoleacetic acid	0.003600515	2.097871366
Armillaramide	0.007195361	2.11899606
Mangiferdesmethylursanone	0.02052541	2.14522004
3-Dehydroteasterone	0.020183518	1.651542071
N-Ornithyl-L-taurine	0.006984676	1.920422456
L-Allothreonine	0.045001304	1.740586109

Significant difference was calculated as adjusted P value<0.05, VIP>1. Other kynurenine pathway metabolites were less (KynA) or not significant (quinolinic acid and 3-hydroxykynurenine). The Kyn/KynA ratio was not significant between groups (P=0.997).

Table S5. Patients and cells information of single-nucleus sequencing.

Groups	mKyn group(n=2)		hKyn group (n=2)	
Patient number	No.1	No.2	No.3	No.4
Age (year)	2	5	4	9
Weight (kg)	4	15.5	8.5	9.5
Gender	male	female	female	female
Kyn (nmol/L)	1283.31	1175.43	3064.18	3086.56
LVPWd-Z score	1.71	-0.32	3.19	3
LVMI	53.5	71.6	127.3	131.6
EF%	73.19	71.99	62.05	60.1
Diagnosis	SAS/LVOTO	SAS/LVOTO	AS/SAS/LVOTO	SAS/LVOTO
Total cell collected	14665	5505	11803	9013
Mean reads per cell	43028	91206	31504	64339
Median genes per cell	1692	1928	1672	1852
Cells after filtering	14581	3947	11797	8983

mKyn= mild elevated Kyn; hKyn= highly elevated Kyn; AS=aortic stenosis; SAS= subvalvular aortic stenosis; LVOTO=left ventricular outflow tract obstruction.

Table S6. Mice heat rate in each echocardiography evaluations

Figure and groups	Heart rates	P value
Fig.S3		0.811
sham (n=3)	501±34	
nAAC (n=3)	494±32	
Fig.3		0.967
WT sham+saline (n=6)	443±30	
WT sham+Kyn (n=6)	451±30	
WT nAAC+saline (n=8)	451±30	
WT nAAC+Kyn (n=10)	456±46	
AHR$^{-/-}$ sham+saline (n=6)	463±45	
AHR$^{-/-}$ sham+Kyn (n=6)	453±48	
AHR$^{-/-}$ nAAC+saline (n=8)	464±30	
AHR$^{-/-}$ nAAC+Kyn (n=8)	448±28	
Fig.7		0.317
sham (n=7)	478±65	
nAAC (n=12)	488±66	
nAAC+probiotics (n=36)	462±46	

Student t test was used in Fig.S3. 1-way ANOVA followed by the Bonferroni post hoc analysis were used for Fig.3 and Fig.7.

Table S7. Mice gender information and Kyn concentration of genders in each evaluation at P21 and P28

Figure and groups	Male	Female	P value
Figure S3E-F			0.928
Sham (n=8)	3	5	
nAAC (n=5)	2	3	
Figure S3G-K			
Each group (n=3)	2	1	1
Figure S4			
Each group (n=5)	2	3	1
Figure S7			0.849
sham (n=29)	12 (941.3±379.3)	17 (803.0±339.0)	(0.206)
nAAC (n=51)	20 (1809.5±1100.7)	31(1708.6±693.5)	(0.629)
Figure 3H			0.816
WT sham+saline (n=6)	4	2	
WT sham+Kyn (n=6)	3	3	
WT nAAC+saline (n=8)	5	3	
WT nAAC+Kyn (n=10)	7	3	
AHR$^{-/-}$ sham+saline (n=6)	2	4	
AHR$^{-/-}$ sham+Kyn (n=6)	3	3	
AHR$^{-/-}$ nAAC+saline (n=8)	3	5	
AHR$^{-/-}$ nAAC+Kyn (n=8)	4	4	
Figure S9			
Each group (n=5)	3	2	1
Figure 6A			0.846
GF mice (n=10)	5 (545.8±85.3)	5 (553.9±106.3)	(0.896)
normal mice (n=28)	13 (916.9±327.6)	15 (828.5±403.3)	(0.534)
Figure 6B			
nAAC donor			1
Pre-FMT (n=10)	5 (532.74±106.27)	5 (542.22±109.64)	(0.963)
Post-FMT (n=10)	5 (1591.07±655.29)	5 (1285.53±557.50)	(0.142)
sham donor			1
Pre-FMT (n=10)	5 (506.03±93.01)	5 (481.30±54.73)	(0.904)
Post-FMT (n=10)	5 (670.09±151.52)	5 (792.94±154.29)	(0.549)
Figure 6H			(0.406)
sham (n=15)	9 (770.2±267.0)	6 (898.4±128.5)	(0.298)
nAAC (n=19)	11 (1575.7±804.6)	8 (1288.7±227.1)	(0.344)
nAAC+probiotics (n=20)	8 (1103.7±119.1)	12 (1109.2±342.3)	(0.966)
Figure 6I			1
Post-FMT (n=10)	5 (1386.61±373.1)	5 (1395.0±432.3)	(0.975)
Post-probiotics (n=10)	5 (1164.4±265.1)	5 (957.9±269.7)	(0.257)
Figure S23A-B			
P21 sham (n=7)	3	4	0.997
P21 nAAC (n=7)	3	4	
P21 nAAC+probiotics(n=9)	4	5	
P28 sham (n=8)	3	5	0.836
P28 nAAC (n=8)	4	4	
P28 nAAC+probiotics (n=12)	6	6	
Figure S23E-F			1
Each group (n=5)	3	2	
Figure 7A-F			0.985
sham (n=7)	3	4	
nAAC (n=12)	5	7	
nAAC+probiotics (n=36)	16	20	

The Kyn concentration of each gender was presented by mean ± SD in brackets and tested by Student t-test. Chi-squared Test were used for determine gender difference.

Table S8. Primer sequences used in real time quantitative RT-PCR

Gene	Species	Forward	Reverse
SLC7A5	mouse	CATTGTCACACTGGTCTATGTG	GAACAGAGACCCATTGACAGAG
AHR	mouse	CATCGACATAACGGACGAAATC	CTGTTGCTGTTGCTCTAGTTG
FN1	mouse	CTATAGGATTGGAGACACGTGG	CTGAAGCACTTTGTAGAGCATG
FN1	human	AATAGATGCAACGATCAGGACA	GCAGGTTTCCTCGATTATCCTT
COL1A1	mouse	TGAACGTGGTGTACAAGGTC	CCATCTTTACCAGGAGAACCAT
COL1A1	human	AAAGATGGACTCAACGGTCTC	CATCGTGAGCCTTCTCTTGAG
ADAMTS2	mouse	ACTGTGATGTGGAACTGGCAAGC	AGTGGAAGGTGGTGTCTGGAGAAG
ADAMTS2	human	TCTCCATCTCCTGACCTTGTGATCC	GACTCTGAACTTCTGCCTCCATCTTC
IDO1	mouse	TGGGACAGATGGGTAAAGGACAGAG	GGAGCATAGACAGCACTTGGAAGC
TDO2	mouse	CTGGCTGTCCTGGAACTCACTTTG	TTGAAGATGACCACCACACGATGC
ANP	mouse	GGTCTAGTGGGGTCTTGCCTCTC	GCGTCTGTCCTTGGTGCTGAAG
BNP	mouse	TGCTGGAGCTGATAAGAGAAAA	GAAGGACTCTTTTTGGGTGTTC
CTGF	mouse	AAAGCAGCTGCAAATACCAATG	AAATGTGTCTTCCAGTCGGTAG
β-MHC	mouse	TTCGCCCTTTGTTCCCATTGTCTC	GGTTGACGGTGACGCAGAAGAG
ZO-1	mouse	GAGCAGGCACAGGTAGGATGAG	GAGGCGGAGGCAGGAGGAG
Occludin	mouse	CTGATGCCCTCTTCTGCCCTTC	CCTGCCTCTGCCTTCCAAGTG
Cdh1	mouse	ATCCCAGCCTTGCCGATTTCC	CTGCCTGCCTCTGCCTTCTG
UniF340	mouse	ACTCCTACGGGAGGCAGCAGT	ATTACCGCGGCTGCTGGC
KY038178.1	mouse	GGAAGAACACCAGTGGCGAAGG	GGCGGAAACCCTCCAACACTTAG
NR_145535.1	mouse	GTGAGTAATGCGTGACCGACCTG	TTTCCCAGAAGACCATGCGATCAAC
NR_040789.1	mouse	AGGCGGCTCTCTGGTCTGTAAC	GGAAACCCTCCAACACTTAGCACTC

KY038178.1 refers to *Lactobacillus acidophilus 145* strain 16S ribosomal RNA gene, partial sequence; *NR_145535.1* refers to *Bifidobacterium longum* 913 strain Su 851 16S ribosomal RNA, partial sequence; *NR_040789.1* refers to *Enterococcus faecalis* strain ATCC19433 16S ribosomal RNA, partial sequence; *UniF340* is the reference gene for above gut microbiota species.

Table S9. Antibodies used in western blot

Target antigen	Vendor	Catalog#	Concentration
Rabbit anti-AHR antibody	Novus	NB100-2289	1:1000
Rabbit anti-ADAMTS2 antibody	Absin	abs146651	1:500
Rabbit anti-FN1 antibody	Abcam	ab2413	1:1000
Rabbit anti-COL1A1 antibody	Cell Signaling Technology	72026S	1:1000
Rabbit anti-Aurora B antibody	Abcam	ab287960	1:300
Rabbit anti-Ki67 antibody	Abcam	ab15580	1:300
Rabbit anti-PH3 antibody	Abcam	ab80612	1:300
Rabbit anti-VEGF antibody	Abcam	ab52917	1:100
Rabbit anti-CD31 antibody	Abcam	ab182981	1:500
Rabbit anti-Tubulin antibody	Cell Signaling Technology	5568	1:1000
Rabbit anti-Histone H3 antibody	Cell Signaling Technology	4499	1:1000
Rabbit anti-GAPDH antibody	Cell Signaling Technology	5174	1:1000

Movie S1.

Surgical procedure of neonatal ascending aorta constriction.

REFERENCES AND NOTES

1. G. H. Kim, N. Uriel, D. Burkhoff, Reverse remodelling and myocardial recovery in heart failure. *Nat. Rev. Cardiol.* **15**, 83–96 (2018).

2. R. B. Hinton, S. M. Ware, Heart failure in pediatric patients with congenital heart disease. *Circ. Res.* **120**, 978–994 (2017).

3. C. M. Otto, Aortic stenosis: Even mild disease is significant. *Eur. Heart J.* **25**, 185–187 (2004).

4. P. A. Pellikka, M. E. Sarano, R. A. Nishimura, J. F. Malouf, K. R. Bailey, C. G. Scott, M. E. Barnes, A. J. Tajik, Outcome of 622 adults with asymptomatic, hemodynamically significant aortic stenosis during prolonged follow-up. *Circulation* **111**, 3290–3295 (2005).

5. C. F. Azevedo, M. Nigri, M. L. Higuchi, P. M. Pomerantzeff, G. S. Spina, R. O. Sampaio, F. Tarasoutchi, M. Grinberg, C. E. Rochitte, Prognostic significance of myocardial fibrosis quantification by histopathology and magnetic resonance imaging in patients with severe aortic valve disease. *J. Am. Coll. Cardiol.* **56**, 278–287 (2010).

6. M. G. Del Buono, R. Arena, B. A. Borlaug, S. Carbone, J. M. Canada, D. L. Kirkman, R. Garten, P. Rodriguez-Miguelez, M. Guazzi, C. J. Lavie, A. Abbate, Exercise intolerance in patients with heart failure. *J. Am. Coll. Cardiol.* **73**, 2209–2225 (2019).

7. J. A. Hill, E. N. Olson, Cardiac plasticity. *N. Engl. J. Med.* **358**, 1370–1380 (2008).

8. C. Andersson, C. Liu, S. Cheng, T. J. Wang, R. E. Gerszten, M. G. Larson, R. S. Vasan, Metabolomic signatures of cardiac remodelling and heart failure risk in the community. *ESC Heart Fail.* **7**, 3707–3715 (2020).

9. Z. Wang, E. Klipfell, B. J. Bennett, R. Koeth, B. S. Levison, B. Dugar, A. E. Feldstein, E. B. Britt, X. Fu, Y. M. Chung, Y. Wu, P. Schauer, J. D. Smith, H. Allayee, W. H. Tang, J. A. DiDonato, A. J. Lusis, S. L.

Hazen, Gut flora metabolism of phosphatidylcholine promotes cardiovascular disease. *Nature* **472**, 57–63 (2011).

10. W. H. Tang, Z. Wang, B. S. Levison, R. A. Koeth, E. B. Britt, X. Fu, Y. Wu, S. L. Hazen, Intestinal microbial metabolism of phosphatidylcholine and cardiovascular risk. *N. Engl. J. Med.* **368**, 1575–1584 (2013).

11. I. Nemet, P. P. Saha, N. Gupta, W. Zhu, K. A. Romano, S. M. Skye, T. Cajka, M. L. Mohan, L. Li, Y. Wu, M. Funabashi, A. E. Ramer-Tait, S. V. Naga Prasad, O. Fiehn, F. E. Rey, W. H. W. Tang, M. A. Fischbach, J. A. DiDonato, S. L. Hazen, A cardiovascular disease-linked gut microbial metabolite acts via adrenergic receptors. *Cell* **180**, 862–877.e22 (2020).

12. M. Schwenk, U. Gundert-Remy, G. Heinemeyer, K. Olejniczak, R. Stahlmann, W. Kaufmann, H. M. Bolt, H. Greim, E. von Keutz, H. P. Gelbke, Children as a sensitive subgroup and their role in regulatory toxicology: DGPT workshop report. *Arch. Toxicol.* **77**, 2–6 (2003).

13. A. Sandek, J. Bauditz, A. Swidsinski, S. Buhner, J. Weber-Eibel, S. von Haehling, W. Schroedl, T. Karhausen, W. Doehner, M. Rauchhaus, P. Poole-Wilson, H. D. Volk, H. Lochs, S. D. Anker, Altered intestinal function in patients with chronic heart failure. *J. Am. Coll. Cardiol.* **50**, 1561–1569 (2007).

14. A. Sandek, A. Swidsinski, W. Schroedl, A. Watson, M. Valentova, R. Herrmann, N. Scherbakov, L. Cramer, M. Rauchhaus, A. Grosse-Herrenthey, M. Krueger, S. von Haehling, W. Doehner, S. D. Anker, J. Bauditz, Intestinal blood flow in patients with chronic heart failure: A link with bacterial growth, gastrointestinal symptoms, and cachexia. *J. Am. Coll. Cardiol.* **64**, 1092–1102 (2014).

15. N. Boccella, R. Paolillo, L. Coretti, S. D'Apice, A. Lama, G. Giugliano, G. G. Schiattarella, M. Cuomo, I. d'Aquino, G. Cavaliere, O. Paciello, M. P. Mollica, G. Mattace Raso, G. Esposito, F. Lembo, C. Perrino, Transverse aortic constriction induces gut barrier alterations, microbiota remodeling and systemic inflammation. *Sci. Rep.* **11**, 7404 (2021).

16. B. O. Schroeder, F. Bäckhed, Signals from the gut microbiota to distant organs in physiology and disease. *Nat. Med.* **22**, 1079–1089 (2016).

17. W. H. Tang, T. Kitai, S. L. Hazen, Gut microbiota in cardiovascular health and disease. *Circ. Res.* **120**, 1183–1196 (2017).

18. J. E. Cheong, L. Sun, Targeting the IDO1/TDO2-KYN-AhR pathway for cancer immunotherapy—Challenges and opportunities. *Trends Pharmacol. Sci.* **39**, 307–325 (2018).

19. M. C. Lafita-Navarro, M. Kim, N. Borenstein-Auerbach, N. Venkateswaran, Y. H. Hao, R. Ray, T. Brabletz, P. P. Scaglioni, J. W. Shay, M. Conacci-Sorrell, The aryl hydrocarbon receptor regulates nucleolar activity and protein synthesis in MYC-expressing cells. *Genes Dev.* **32**, 1303–1308 (2018).

20. Y. Liu, X. Liang, W. Dong, Y. Fang, J. Lv, T. Zhang, R. Fiskesund, J. Xie, J. Liu, X. Yin, X. Jin, D. Chen, K. Tang, J. Ma, H. Zhang, J. Yu, J. Yan, H. Liang, S. Mo, F. Cheng, Y. Zhou, H. Zhang, J. Wang, J. Li, Y. Chen, B. Cui, Z. W. Hu, X. Cao, F. Xiao-Feng Qin, B. Huang, Tumor-repopulating cells induce PD-1 expression in CD8(+) T cells by transferring kynurenine and AhR activation. *Cancer Cell* **33**, 480–494.e7 (2018).

21. K. Kawajiri, Y. Kobayashi, F. Ohtake, T. Ikuta, Y. Matsushima, J. Mimura, S. Pettersson, R. S. Pollenz, T. Sakaki, T. Hirokawa, T. Akiyama, M. Kurosumi, L. Poellinger, S. Kato, Y. Fujii-Kuriyama, Aryl hydrocarbon receptor suppresses intestinal carcinogenesis in ApcMin/+ mice with natural ligands. *Proc. Natl. Acad. Sci. U.S.A.* **106**, 13481–13486 (2009).

22. T. Ikuta, Y. Kobayashi, M. Kitazawa, K. Shiizaki, N. Itano, T. Noda, S. Pettersson, L. Poellinger, Y. Fujii-Kuriyama, S. Taniguchi, K. Kawajiri, ASC-associated inflammation promotes cecal tumorigenesis in aryl hydrocarbon receptor-deficient mice. *Carcinogenesis* **34**, 1620–1627 (2013).

23. J. Yang, K. Savvatis, J. S. Kang, P. Fan, H. Zhong, K. Schwartz, V. Barry, A. Mikels-Vigdal, S. Karpinski, D. Kornyeyev, J. Adamkewicz, X. Feng, Q. Zhou, C. Shang, P. Kumar, D. Phan, M. Kasner,

B. López, J. Diez, K. C. Wright, R. L. Kovacs, P. S. Chen, T. Quertermous, V. Smith, L. Yao, C. Tschöpe, C. P. Chang, Targeting LOXL2 for cardiac interstitial fibrosis and heart failure treatment. *Nat. Commun.* **7**, 13710 (2016).

24. C. A. Opitz, U. M. Litzenburger, F. Sahm, M. Ott, I. Tritschler, S. Trump, T. Schumacher, L. Jestaedt, D. Schrenk, M. Weller, M. Jugold, G. J. Guillemin, C. L. Miller, C. Lutz, B. Radlwimmer, I. Lehmann, A. von Deimling, W. Wick, M. Platten, An endogenous tumour-promoting ligand of the human aryl hydrocarbon receptor. *Nature* **478**, 197–203 (2011).

25. C. Koentges, M. E. Pepin, C. Müsse, K. Pfeil, S. V. V. Alvarez, N. Hoppe, M. M. Hoffmann, K. E. Odening, S. Sossalla, A. Zirlik, L. Hein, C. Bode, A. R. Wende, H. Bugger, Gene expression analysis to identify mechanisms underlying heart failure susceptibility in mice and humans. *Basic Res. Cardiol.* **113**, 8 (2018).

26. X. Hua, Y. Y. Wang, P. Jia, Q. Xiong, Y. Hu, Y. Chang, S. Lai, Y. Xu, Z. Zhao, J. Song, Multi-level transcriptome sequencing identifies COL1A1 as a candidate marker in human heart failure progression. *BMC Med.* **18**, 2 (2020).

27. C. D. Rau, M. C. Romay, M. Tuteryan, J. J. Wang, M. Santolini, S. Ren, A. Karma, J. N. Weiss, Y. Wang, A. J. Lusis, Systems genetics approach identifies gene pathways and Adamts2 as drivers of isoproterenol-induced cardiac hypertrophy and cardiomyopathy in mice. *Cell Syst.* **4**, 121–128.e4 (2017).

28. X. Wang, W. Chen, J. Zhang, A. Khan, L. Li, F. Huang, Z. Qiu, L. Wang, X. Chen, Critical role of ADAMTS2 (a disintegrin and metalloproteinase with thrombospondin motifs 2) in cardiac hypertrophy induced by pressure overload. *Hypertension* **69**, 1060–1069 (2017).

29. T. Zelante, R. G. Iannitti, C. Cunha, A. De Luca, G. Giovannini, G. Pieraccini, R. Zecchi, C. D'Angelo, C. Massi-Benedetti, F. Fallarino, A. Carvalho, P. Puccetti, L. Romani, Tryptophan catabolites from

microbiota engage aryl hydrocarbon receptor and balance mucosal reactivity via interleukin-22. *Immunity* **39**, 372–385 (2013).

30. M. C. Simon, K. Strassburger, B. Nowotny, H. Kolb, P. Nowotny, V. Burkart, F. Zivehe, J. H. Hwang, P. Stehle, G. Pacini, B. Hartmann, J. J. Holst, C. MacKenzie, L. B. Bindels, I. Martinez, J. Walter, B. Henrich, N. C. Schloot, M. Roden, Intake of Lactobacillus reuteri improves incretin and insulin secretion in glucose-tolerant humans: a proof of concept. *Diabetes Care* **38**, 1827–1834 (2015).

31. S. Khalesi, J. Sun, N. Buys, R. Jayasinghe, Effect of probiotics on blood pressure. *Hypertension* **64**, 897–903 (2014).

32. R. M. Thushara, S. Gangadaran, Z. Solati, M. H. Moghadasian, Cardiovascular benefits of probiotics: A review of experimental and clinical studies. *Food Funct.* **7**, 632–642 (2016).

33. M. M. Rinschen, J. Ivanisevic, M. Giera, G. Siuzdak, Identification of bioactive metabolites using activity metabolomics. *Nat. Rev. Mol. Cell Biol.* **20**, 353–367 (2019).

34. P. Song, T. Ramprasath, H. Wang, M. H. Zou, Abnormal kynurenine pathway of tryptophan catabolism in cardiovascular diseases. *Cell. Mol. Life Sci.* **74**, 2899–2916 (2017).

35. S. Adams, N. Braidy, A. Bessede, B. J. Brew, R. Grant, C. Teo, G. J. Guillemin, The kynurenine pathway in brain tumor pathogenesis. *Cancer Res.* **72**, 5649–5657 (2012).

36. Y. Wang, H. Liu, G. McKenzie, P. K. Witting, J. P. Stasch, M. Hahn, D. Changsirivathanathamrong, B. J. Wu, H. J. Ball, S. R. Thomas, V. Kapoor, D. S. Celermajer, A. L. Mellor, J. F. Keaney, Jr., N. H. Hunt, R. Stocker, Kynurenine is an endothelium-derived relaxing factor produced during inflammation. *Nat. Med.* **16**, 279–285 (2010).

37. T. B. Dschietzig, K. H. Kellner, K. Sasse, F. Boschann, R. Klüsener, J. Ruppert, F. P. Armbruster, D. Bankovic, A. Meinitzer, V. Mitrovic, C. Melzer, Plasma kynurenine predicts severity and complications

of heart failure and associates with established biochemical and clinical markers of disease. *Kidney Blood Press. Res.* **44**, 765–776 (2019).

38. M. Ala, S. P. Eftekhar, The footprint of kynurenine pathway in cardiovascular diseases. *Int. J. Tryptophan. Res.* **15**, 117864692210966 (2022).

39. T. Bekfani, M. Bekhite, S. Neugebauer, S. Derlien, A. Hamadanchi, J. Nisser, M. S. Hilse, D. Haase, T. Kretzschmar, M. F. Wu, M. Lichtenauer, M. Kiehntopf, S. von Haehling, P. Schlattmann, G. Lehmann, M. Franz, S. Möbius-Winkler, C. Schulze, Metabolomic profiling in patients with heart failure and exercise intolerance: Kynurenine as a potential biomarker. *Cell* **11**, (2022).

40. P. M. Fernandez-Salguero, J. M. Ward, J. P. Sundberg, F. J. Gonzalez, Lesions of aryl-hydrocarbon receptor-deficient mice. *Vet. Pathol.* **34**, 605–614 (1997).

41. D. Zhang, J. Ning, T. Ramprasath, C. Yu, X. Zheng, P. Song, Z. Xie, M. H. Zou, Kynurenine promotes neonatal heart regeneration by stimulating cardiomyocyte proliferation and cardiac angiogenesis. *Nat. Commun.* **13**, 6371 (2022s).

42. L. Laurans, N. Venteclef, Y. Haddad, M. Chajadine, F. Alzaid, S. Metghalchi, B. Sovran, R. G. P. Denis, J. Dairou, M. Cardellini, J. M. Moreno-Navarrete, M. Straub, S. Jegou, C. McQuitty, T. Viel, B. Esposito, B. Tavitian, J. Callebert, S. H. Luquet, M. Federici, J. M. Fernandez-Real, R. Burcelin, J. M. Launay, A. Tedgui, Z. Mallat, H. Sokol, S. Taleb, Genetic deficiency of indoleamine 2,3-dioxygenase promotes gut microbiota-mediated metabolic health. *Nat. Med.* **24**, 1113–1120 (2018).

43. B. Lamas, M. L. Richard, V. Leducq, H. P. Pham, M. L. Michel, G. Da Costa, C. Bridonneau, S. Jegou, T. W. Hoffmann, J. M. Natividad, L. Brot, S. Taleb, A. Couturier-Maillard, I. Nion-Larmurier, F. Merabtene, P. Seksik, A. Bourrier, J. Cosnes, B. Ryffel, L. Beaugerie, J. M. Launay, P. Langella, R. J. Xavier, H. Sokol, CARD9 impacts colitis by altering gut microbiota metabolism of tryptophan into aryl hydrocarbon receptor ligands. *Nat. Med.* **22**, 598–605 (2016).

44. L. O'Rourke, G. Clarke, A. Nolan, C. Watkins, T. G. Dinan, C. Stanton, R. P. Ross, C. A. Ryan, Tryptophan metabolic profile in term and preterm breast milk: Implications for health. *J. Nutr. Sci.* **7**, e13 (2018).

45. M. F. Laursen, M. Sakanaka, N. von Burg, U. Mörbe, D. Andersen, J. M. Moll, C. T. Pekmez, A. Rivollier, K. F. Michaelsen, C. Mølgaard, M. V. Lind, L. O. Dragsted, T. Katayama, H. L. Frandsen, A. M. Vinggaard, M. I. Bahl, S. Brix, W. Agace, T. R. Licht, H. M. Roager, Bifidobacterium species associated with breastfeeding produce aromatic lactic acids in the infant gut. *Nat. Microbiol.* **6**, 1367–1382 (2021).

46. M. Platten, E. A. A. Nollen, U. F. Röhrig, F. Fallarino, C. A. Opitz, Tryptophan metabolism as a common therapeutic target in cancer, neurodegeneration and beyond. *Nat. Rev. Drug Discov.* **18**, 379–401 (2019).

47. G. C. Prendergast, W. P. Malachowski, J. B. DuHadaway, A. J. Muller, Discovery of IDO1 inhibitors: From bench to bedside. *Cancer Res.* **77**, 6795–6811 (2017).

48. T. A. Triplett, K. C. Garrison, N. Marshall, M. Donkor, J. Blazeck, C. Lamb, A. Qerqez, J. D. Dekker, Y. Tanno, W. C. Lu, C. S. Karamitros, K. Ford, B. Tan, X. M. Zhang, K. McGovern, S. Coma, Y. Kumada, M. S. Yamany, E. Sentandreu, G. Fromm, S. Tiziani, T. H. Schreiber, M. Manfredi, L. I. R. Ehrlich, E. Stone, G. Georgiou, Reversal of indoleamine 2,3-dioxygenase-mediated cancer immune suppression by systemic kynurenine depletion with a therapeutic enzyme. *Nat. Biotechnol.* **36**, 758–764 (2018).

49. P. G. Rusconi, D. A. Ludwig, C. Ratnasamy, R. Mas, W. G. Harmon, S. D. Colan, S. E. Lipshultz. Serial measurements of serum NT-proBNP as markers of left ventricular systolic function and remodeling in children with heart failure. *Am. Heart J.* **160**, 776–83 (2010).

50. R. B. Devereux, D. R. Alonso, E. M. Lutas, G. J. Gottlieb, E. Campo, I. Sachs, N. Reichek. Echocardiographic assessment of left ventricular hypertrophy: Comparison to necropsy findings. *Am. J. Cardiol.* **57**,450–458 (1986).

51. Y. Gong, Z. Chen, L. Yang, X. Ai, B. Yan, H. Wang, L. Qiu, Y. Tan, N. Witman, W. Wang, Y. Zhao, W. Fu, Intrinsic color sensing system allows for real-time observable functional changes on human induced pluripotent stem cell-derived cardiomyocytes. *ACS Nano* **14**, 8232–8246 (2020).

52. Y. Liu, Q. Luo, Z. Su, J. Xing, J. Wu, L. Xiang, Y. Huang, H. Pan, X. Wu, X. Zhang, J. Li, F. Yan, H. Zhang, Suppression of myocardial hypoxia-inducible factor-1α compromises metabolic adaptation and impairs cardiac function in patients with cyanotic congenital heart disease during puberty. *Circulation* **143**, 2254–2272 (2021).
